# Co-Developed Community-Based Health Interventions with Children Under 18 and Families Experiencing Homelessness in High-Income Countries: A Systematic Review

**DOI:** 10.3390/healthcare14040492

**Published:** 2026-02-14

**Authors:** Diana Margot Rosenthal, Jasia Kubik, Sabrina Loureiro, Kate Guastaferro, Melody Goodman

**Affiliations:** 1 Department of Social and Behavioral Sciences, School of Global Public Health, New York University, New York, NY 10003, USA; 2 Robert F. Wagner School of Public Service, New York University, New York, NY 10003, USA; ak8369@nyu.edu; 3 Silver School of Social Work, New York University, New York, NY 10003, USA; 4 Department of Biostatistics, School of Global Public Health, New York University, New York, NY 10003, USA

**Keywords:** homelessness, shelter, inclusion health, health equity, health disparities, community engagement, co-development, co-creation, co-production, co-design, reciprocity, health promotion, community-based participatory research, participatory methods, community-based intervention, community health, children, families

## Abstract

Background: Despite the implementation of numerous evidence-based interventions, the 2024 Point-in-Time count in the United States (U.S.) reported that 259,473 people in families with children under 18 years old were experiencing homelessness, a record high since the count began in 2007. Recent findings suggest that co-developed interventions may increase engagement with vulnerable populations and, in turn, the effectiveness of health-based programs among them. Objective: In this review, we sought to systematically search and assess the current evidence on co-developed community-based interventions with and for children under age 18 and families experiencing homelessness (CFEH) in high-income countries and their impact on health and well-being outcomes. Methods: Seven databases (e.g., Medline, CINAHL, Embase) and four additional scholarly sources (e.g., Health CASCADE) were searched (publication dates between January 2000 and February 2025). In our analysis, methodological “quality” was assessed through two primary criteria: internal validity and the extent of CFEH involvement. Results: A total of 1617 studies were screened for eligibility, and nine studies were found to have co-developed interventions with CFEH in the U.S. (*n* = 6) and the United Kingdom (*n* = 3). These were categorized thematically by socio-structural, behavioral, and combined intervention types. Five studies reported positive engagement among families and staff, whereas three reported improved mental health outcomes. Conclusions: This review highlights the potential impact of co-developed interventions on CFEH’s mental and physical well-being as well as process-based outcomes. Limitations include different definitions of “co-” terminology and homelessness across studies, as well as a lack of transparency about the extent of CFEH’s involvement in these studies. The dearth of evidence indicates that future research should employ community-based participatory research while striking a balance of working with CFEH and other partners and ensuring the data are reliable and reproducible.

## 1. Introduction

### 1.1. Background

The 2024 United States (U.S.) Housing and Urban Development (HUD) Point-in-Time (PIT) count revealed 259,473 people in families with children under 18 years of age (under-18s) experienced homelessness, a record high since the count began in 2007 [1]. Notably, this was a 39% increase from the previous 2023 PIT count, reflecting a nationwide housing crisis [1]. Although there is no universal definition of *homelessness*, in the U.S., the McKinney-Vento Homelessness Assistance Act [2] provides standard benchmarks upon which federal and state governments rely to provide and evaluate services. Accordingly, individuals experiencing homelessness are those who “lack a fixed, regular, and adequate nighttime residence.” [2]. Coincidentally, while rental costs rose significantly between 2001 and 2021, wages have remained mostly stagnant [3]. This disproportionate increase in costs has been especially difficult for families with children under 18. Along with higher rents, childcare costs have far exceeded the rate of inflation [3], posing economic hardships on families across the U.S. and putting them at greater risk for housing insecurity and homelessness.

### 1.2. Health Disparities Among Homeless Populations

Although people experiencing homelessness have higher rates of poor mental and physical health outcomes and are more likely to report co-occurring illnesses, they also report the highest barriers to accessing care [4]. Without secure housing, people experiencing homelessness are more vulnerable than the general population to communicable diseases (e.g., tuberculosis [TB], HIV, hepatitis, and COVID-19) and to non-communicable diseases, such as diabetes, cardiovascular disease, and mental illness (e.g., anxiety, depression, post-traumatic stress disorder, substance use disorder, etc.) [5,6]. For example, rates of TB in people experiencing homelessness were reported to be 40 times higher than the general U.S. population [7]; people experiencing homelessness also had more than two times the odds of dying from TB [8]. Compared with the general population, individuals experiencing homelessness faced a mortality risk that was 2 to 5 times higher [8]. Additionally, 65% were more frequently evaluated in the emergency department for COVID-19 and hospitalized [8,9]. Moreover, young children within families experiencing homelessness experience higher rates of developmental issues [10] as well as the other aforementioned health issues. Beyond the environmental factors at play, various social determinants and reported barriers to health care access may contribute to poorer health outcomes among families of such children experiencing homelessness—barriers such as safety concerns, competing priorities (i.e., housing, food, childcare, etc.), lack of health insurance, lack of transportation, and financial difficulties [11,12]. Given the unique barriers these children and families face, tailored interventions are necessary to address these challenges and health disparities.

### 1.3. Co-Developing Interventions with Vulnerable Populations

Co-developing interventions, especially for vulnerable populations such as people experiencing homelessness, has the potential to improve service delivery efficiency and effectiveness. Integrating principles of community-based participatory research (CBPR) into this process further strengthens intervention development by ensuring equitable involvement of community members throughout all stages of a project [13,14]. Active collaborations and partnerships among researchers, programmatic staff, clinicians, and the population of interest are central to co-developed interventions [15]. CBPR provides a structured framework to authentically share power and decision-making for such collaborative partnerships [13,14]. These partnerships are thoughtfully and intentionally developed to co-create the intervention’s objectives, proposed outcomes, and methodology; they seamlessly integrate with the population’s current facilitators and effectively address barriers to care [13,16]. Previous studies have shown positive outcomes associated with interventions co-developed among diverse populations [17]. Specifically, among vulnerable populations, interventions co-developed through CBPR and people-centered approaches serve as a mechanism for community and academic engagement; this engagement produces interventions that are more reflective of community needs and grounded in their lived experiences. For example, an evaluation of a co-designed handbook for caregivers of youth with opioid use disorder showed that caregivers saw their needs reflected in the intervention, felt supported, and believed in the intervention’s potential impact on their community [18]. However, the interventions in the abovementioned studies were designed and tested with older adults and with adults experiencing homelessness and substance misuse rather than with children under 18 years old or families of such children experiencing homelessness (CFEH), specifically.

Although studies have shown positive outcomes of co-developed interventions in some populations, little is known about the potential health outcomes of such interventions with CFEH. In this review (PROSPERO ID: CRD42024599973), we aimed to systematically search for and assess the existing literature and evidence on community-based interventions co-developed with and for CFEH in high-income countries (HICs) and their impact on health and well-being outcomes.

## 2. Methods

This review followed the PRISMA (Preferred Reporting Items for Systematic Reviews and Meta-Analyses) reporting guidelines [19] to ensure the review was conducted transparently and comprehensively (Table S1: PRISMA Checklists). The PICO (population, intervention, comparison, and outcome) framework helped define the inclusion/exclusion criteria and guided the search strategy [20]. Table 1 shows the inclusion and exclusion criteria for the review.

In this review, the World Bank’s definition of *HICs* [21] and the McKinney-Vento definition [2] for *homelessness* were used. The World Health Organization broadly defines *health* as “…a state of complete physical, mental and social well-being and not merely the absence of disease or infirmity.” [22]. In this review, health and well-being outcomes were defined as health impacts stemming from a condition, event, or intervention [23]. A community-based intervention was generally defined as a program or service to improve and/or maintain the human health and well-being of specific population groups while targeting the individual, family, community, and systems levels, or any combination of these levels [24,25]. In this review, an intervention that has been co-developed or co-produced actively involves researchers and all relevant community partners working together in the assessment, design, implementation, evaluation, AND dissemination stages in a continuous reciprocal and equal relationship [17,26]. Many “co-” terms (e.g., “co-create,” “co-construct,” “co-design,” “co-produce,” “co-develop,” “co-facilitate”) are often used interchangeably. We decided that one of these “co-” terms or the use of CBPR should be included in the text for this review to maintain consistency in our approach [27]. When the terms “collaboration,” “collaborative,” or “consultative” were used independently and exclusively, they were approached cautiously and often excluded from our review because of their ambiguous usage and the unclear extent to which they were applied. On the other hand, CBPR is a distinct research approach that is co-created with researchers and community members (e.g., CFEH) at all stages and involves a step-by-step process [28] to specifically address community issues, even if “co-” terms are not explicitly used. We considered this selection method straightforward, which helped ensure that all reviewers were consistent in their evaluations. We also believed this method reduced the risk of personal bias or interpretation differences, especially given the limited agreement on the specific terminology used.

### 2.1. Search Strategy

Because of the likelihood of broad, multidisciplinary interest in this topic, multiple databases were searched across the health and social sciences: Medline (via PubMed), Cumulative Index to Nursing and Allied Health Literature (CINAHL), Embase (via Ovid), PsycInfo (via Ovid), Global Health (via Ovid), SocIndex (via Ebsco), and ERIC/Education Source (via Ebsco). A search strategy was initially developed in PubMed with assistance from a university-based public health librarian and was subsequently adapted to other databases and platforms [29]. The core strategy comprised three to four concepts, depending on the subject area of the database being searched. For health science databases, the concepts were *homelessness*, *co-development*, and *children/families*. An additional concept related to health outcomes was included in databases focused outside of health (ERIC/Education Source and SocIndex). We included databases with a relevant subject focus that were indexed using subject headings. Broad, multidisciplinary databases without subject headings, such as Scopus and the Web of Science Core Collection, were not feasible to include due to very low precision in pilot searches. For each concept, a combination of keywords and subject headings was combined with “OR,” and the concepts were subsequently combined with “AND.” The searches were broad by design to capture records that explicitly mentioned “homelessness” and “co-development,” as well as those that may have included the population of interest within a broader population or that used other terminology.

In order to limit the search results to HICs, a filter was used to remove records centered on low- or middle-income countries (LMICs) unless they also mentioned HICs: NOT (“LMICs” NOT “HICs”). This should have returned records that included location information indicating an HIC, as well as records without location information included. Additional filters for publication dates (2000-February 2025), language (English), and study type were used in some databases. We began with literature published since 2000 to ensure that our current study, Co-developing SHELTER [30,31], was informed by recent research in this evolving field. By the 2000s, homelessness among CFEH had increased significantly in HICs, and CBPR was widely acknowledged and supported by dedicated government and foundation funding (e.g., the W.K. Kellogg Foundation, National Institutes of Health), conferences, and consensus on tenets linking scientific work to community practice [32,33,34]. Details of each strategy are included in the Supplementary Table S2: Search Strategies.

Because of the heterogeneity of terms and definitions within the subject area of co-development, additional sources were reviewed to complement the database searches. The Collaborative Indigenous Research Digital Garden [35] and the Health CASCADE Co-Creation Database [36] are databases of reports related to co-development and, more broadly, participatory research. The Homelessness Impact Evidence Finder [37] is a similar collection focused on research evaluating homelessness interventions. Finally, a narrow Google Scholar search was conducted, using the most central terminology related to the research question. Table S3: Additional Records provides details of how records were retrieved from these sources.

### 2.2. Data Management and Screening Process

Database searches were performed between 22 November and 13 December 2024. Additional sources were searched, and 292 were downloaded for screening between 21 January and 17 February 2025 (Table S3: Additional Records). The retrieved records were collected in EndNote [38]. Duplicates were removed along with reviews, editorials, commentaries, and replies. The remaining records were then uploaded to Covidence [39]. The title and abstract of each record were screened independently by two reviewers at a time (JK, SL, and DMR). The records not excluded during the screening step were then independently reviewed in full by two reviewers (SL and JK) to assess eligibility based on the inclusion/exclusion criteria. Any conflicts identified during each step were resolved by consensus with a third reviewer (DMR). In order to collect a complete set of reasons for excluding each full-text document, individual spreadsheets were created for each reviewer to select from a set of exclusion reasons [40], which allowed for more than one reason to be selected. When complete, the spreadsheets were compiled by a third party, and conflicts were resolved by consensus with a third reviewer (DMR).

Studies that met the inclusion criteria were selected, and subsequent data extraction was carried out (Table S4: Data Extraction Sheet Example). Data extracted included study and participant characteristics, intervention type, and main outcomes. The initial inter-rater reliability (IRR) was 75.0%, indicating substantial agreement among reviewers [41]. Through consensus meetings for both the screening and data extraction stages, the reviewers referred back to the inclusion criteria to ensure consistency. Due to inconsistent use of “co-” terms in the literature, the third reviewer conducted an additional supplementary screening and full-text review to enhance the rigor of the review process. By using filter tools in Covidence and Excel to search for different variants of “co-develop,” “co-design,” “co-produce,” “co-create,” “co-construct,” and CBPR terms, this approach ensured that our searches and article selections adhered to the inclusion criteria and addressed any inconsistencies. With this thorough process, the reviewers ultimately achieved an IRR of 83.3% for the final included articles in this review, which is considered perfect agreement by Covidence standards [41].

### 2.3. Analysis

In our analysis, methodological “quality” was assessed through two primary criteria: internal validity and the extent of CFEH involvement.

The National Heart, Lung, and Blood Institute (NHLBI) study quality assessment tools were used to critically appraise the internal validity of the included studies [42]. These tools were selected to help identify potential flaws in the study methods or their implementation, including sources of bias, confounding, power, and strength of causality between co-developed interventions and health and well-being outcomes [42]. Three reviewers (SL, JK, and DMR) independently conducted the quality assessments and compared their results. The initial IRR was 82.0%. On the assessment tools, reviewers could select “yes,” “no,” or “cannot determine/not reported/not applicable” for each criterion. “No” indicated a possible risk of bias in the study design or implementation, and “cannot determine/not reported” was noted as a potential study flaw [42]. Lastly, reviewers had to determine whether each study was of “good,” “fair,” or “poor” quality by assessing the prior criteria. A “good” quality rating signified that the study exhibited the least risk of bias, and the results were deemed valid; however, a “fair” rating suggested vulnerability to some bias, but not necessarily enough to undermine the validity of its results. Conversely, a “poor” rating indicated a significant risk of bias [42]. Any disagreements were discussed in depth until a consensus was reached (IRR = 100.0%). Due to the heterogeneity among study outcomes, a meta-analysis was not possible.

Studies were also assessed (DMR and JK) on the basis of increasing levels of CFEH participation (i.e., engage, co-design, co-produce), using a secondary adaptation of Arnstein’s Ladder of Citizen Participation by Homeless Link and Expert Link [26]. This framework was specifically selected because it was co-produced with people with lived experience of homelessness. Since studies were at different points in the research cycle, we also used the stages outlined by Hacker (e.g., community needs assessment, design and methods, analysis and interpretation, dissemination) [28] to indicate which points in the process CFEH were involved in each study. Regardless of the key terminology used in the included texts, this assessment enabled us to benchmark against a set of criteria to distinguish between studies that were genuinely co-produced and those that allotted CFEH to consultative roles (e.g., filling out a survey without further influence).

Results were summarized using a PRISMA flow diagram [19] and tables detailing study characteristics and key findings. Additionally, the role of “co-” terms and the definitions of homelessness across all included studies were analyzed to clarify collaborative methodologies and contextualize research approaches. Results were then grouped thematically and reported based on intervention type: behavioral, socio-structural, or combination [24,43].

## 3. Results

### 3.1. Study Selection

**Figure 1:**
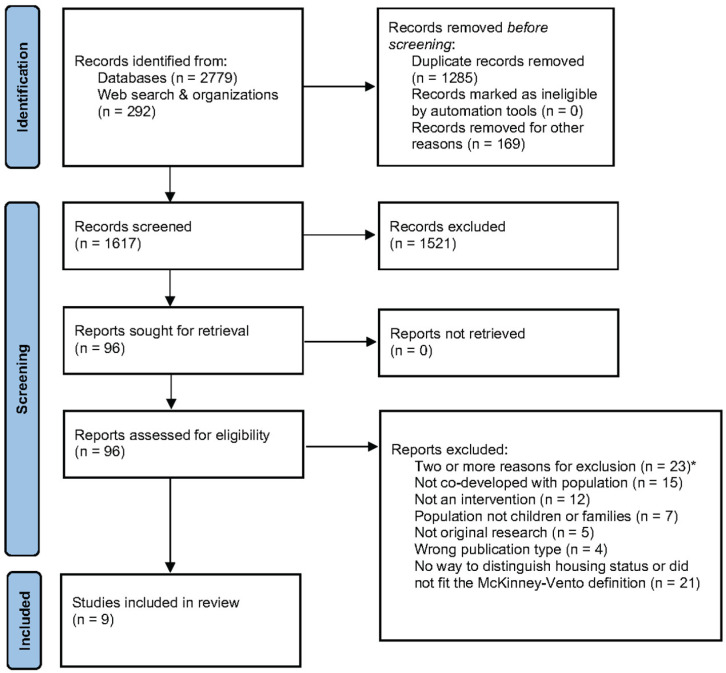
PRISMA diagram for new systematic reviews, which included searches of databases, registers and other sources [19]. Note: Please see Table S3: Additional Records for a detailed breakdown * of the two or more pre-specified exclusion reasons.

A total of 3071 articles published after 2000 were retrieved from databases (*n* = 2779) and other sources (*n* = 292; i.e., Google Scholar, Cascade, and CRCL). After the removal of duplicates, 1617 articles were systematically assessed for eligibility using Covidence [39]. Of these screened articles, 96 studies were reviewed in full, and 87 were deemed ineligible (Figure 1; PRISMA flow diagram [19]). In total, 23 articles were ineligible because of two or more pre-specified exclusion reasons, such as the population of interest being indistinguishable from the broader population. Moreover, 15 articles were excluded for lacking co-development (including other “co-” or CBPR terminology) with the population of interest, and 12 were excluded for a non-intervention study design. Also, the remaining articles were excluded because the studies did not include children under 18 years of age (*n* = 7), were not original research (*n* = 5), or were incorrect publication types (i.e., meeting abstracts, dissertations; *n* = 4); additionally, a study was excluded if it did not describe the population’s housing status or the population was not experiencing homelessness according to the McKinney-Vento definition (*n* = 21). Nine studies were selected for inclusion in this review.

#### Excluded Studies

Several studies initially appeared eligible but were subsequently excluded upon closer review. Some studies were methodologically sound in the co-development area; however, these were not specifically tailored to CFEH. Often, people experiencing homelessness were indistinguishable from people who were “housing insecure”, “socially disadvantaged”, “migrants,” or “marginalized” due to various factors (e.g., mental illness, substance use, domestic violence, former incarceration, and exchanging sex for money or drugs) [44,45]. Some studies only examined the feasibility or acceptability of interventions through partner engagement and feedback [46] without necessarily being co-developed with CFEH. Osman et al. engaged youth experiencing homelessness (YEH) partners of unknown ages (i.e., possibly > 18 years) but also did not involve them in the cross-sector co-development process (e.g., project planning, design, implementation), and at times they were indistinguishable from other partners (e.g., organization staff), leading to exclusion [46]. Another example of a reason for exclusion was when the population informed a minimal component of the study, but more importantly, when the iterative process (i.e., co-development) was conducted only with staff [47]. An adolescent sexual health and pregnancy prevention program used Participatory Action Research (PAR) to co-create knowledge with YEH post-intervention using PhotoVoice; participants were not involved at every stage of the project and had been primarily relegated to a data-collection role, so it was not an equitable/reciprocal relationship [48,49]. In most cases, vague study descriptions and a lack of details on partnerships or outcomes made it impossible to ascertain whether these cases were genuinely co-developed or included CBPR in their methodology unless explicitly stated, ultimately leading to their exclusion (Figure 1).

### 3.2. Study Characteristics

All included studies were conducted in HICs [50,51,52,53,54,55,56,57,58]: Six studies were conducted in the U.S., and three studies in the United Kingdom (U.K.). Interventions were designed for CFEH residing in shelters (*n* = 6) or in long-term supportive housing within the community (*n* = 3) (Table 2).

#### 3.2.1. Participant Characteristics

Most articles focused on families with children (*n* = 7), specifically children ranging from “babies” to 14-year-olds (*n* = 4) [50,54,55,57]. Moreover, two studies did not specify an age group of the children within their inclusion criteria but suggested anecdotally that children under age 5 (under-5s) were included in the child population [53,58]. Two articles, authored by the same investigators, focused solely on youth ages 16 to 24 years [51,52]. The ages of caregivers were not always reported. Although only five articles clearly reported their study population’s gender, women comprised most of the population [51,52,54,56,58]. (Table 2) Sampling bias may have been possible; however, it cannot be definitively determined because of the lack of or ambiguity in socio-demographic data, including ethnicity. Not including sociodemographic data can demonstrate bias because it ignores the fact that some groups are more likely to experience family homelessness; even more so, this affects the generalizability of the findings. In one article, ethnicity was reported without proper clarification of whether it belonged to the sample or to the general population of family homeless shelters [58]. Surprisingly, a case study did not report any sample size [50]. Although Cumming et al. (2022) did not report any demographic information on the 195 participants (i.e., CFEH) in their first study [51], except for other community partner demographics, their second study published demographics for the 15 participants [52]. Lastly, Gewirtz O’Brien et al.’s (2022) target population of pregnant youth was reported, but evidence of pregnancy among the sample was not detailed in the study [53].

#### 3.2.2. Definitions of Homelessness

A diverse range of populations experiencing homelessness was included in the studies, encompassing individuals residing in shelters as well as those in supportive or transitional housing. However, several studies did not provide a clear definition of homelessness at the outset (Table 2 and Table S5: Study Definitions of Homelessness). Notably, one U.K.-based study adopted the U.S. legal definition of homelessness—the McKinney-Vento Act—“the lack of a fixed, regular, and adequate nighttime residence,” [2,52], while another U.K. policy- and arts-focused study neither defined homelessness nor reviewed relevant national policies on temporary accommodation nor thoroughly explained “no recourse to public funds” (NRPF), potentially limiting clarity for an international audience [54]. In the U.K., TA is a provision for homeless households provided by the local council while they wait for an assessment decision or longer-term housing [59]. Refer to Table S5 for the specific definitions of homelessness employed or the lack thereof in each study.

#### 3.2.3. Study Designs

Some articles did not clearly report a study design. Still, all included or informed interventions were without controls, so they were assessed as a before-after (pre-post) design with no control [42] in the design and/or testing of their interventions. Four articles were classified as qualitative (one case study), three used mixed methods, one was a randomized clinical trial, and one did not report a study design. The studies reported an *n* ranging from 15 to 208 (Table 2).

### 3.3. Quality Assessments

#### 3.3.1. Internal Validity

In the assessment stage, four articles were classified as poor quality, indicating a substantial risk of bias, and three as fair quality, suggesting susceptibility to some bias, although not enough to invalidate their findings [42]. Two articles were rated good/fair, given that they reported some strong qualities but still had some risk of bias. Although the studies included reported positive health and well-being outcomes, they also had several limitations. Three cited improvement in mental health symptoms [51,55,57]. Only one study [51] included validated measures that matched the primary outcome. Moreover, in two non-qualitative studies, only superficial findings were reported, without detailed statistical evidence or standardized measures, to substantiate their conclusions, though such data may be available beyond our search [51,55]. Two non-quantitative studies did not include formal evaluation plans to highlight the potential impacts of the intervention [50,54], although some collected strong anecdotal feedback. Studies used pre- and post-intervention designs without control groups, limiting the ability to directly attribute observed outcomes to the interventions rather than to external factors.

Many of the articles did not discuss their methodologies in detail, complicating the quality assessment. Reporting was often insufficient: details on study design, sample size, and demographic information were frequently omitted or unclear, which limited the generalizability of the results in some cases. Methodological shortcomings included the use of non-representative samples for non-qualitative studies, a lack of subgroup analyses, and an absence of clear eligibility criteria. Participant-related challenges further affected study quality, including small sample sizes and low retention rates, thereby increasing the risk of selection bias. Some studies reportedly conducted asset- and needs-based assessments, but one was without documented methodologies or findings [50]. The mixed-methods studies evidently leaned more on their qualitative findings to demonstrate the impact of their interventions [51,52,57]. Regardless of study design, each study had methodological shortcomings; see Supplementary Table S6 for individual study quality assessment ratings and limitations; see Supplementary Table S7 for the detailed quality assessments.

#### 3.3.2. Assessment of Engagement

Although most studies included had lower internal validity, they were recognized for the quality of their participatory methods. Community-based interventions co-developed with and for CFEH frequently utilized CBPR; five out of nine studies explicitly reported using CBPR during the development and/or implementation of their interventions [51,52,53,56,58]. However, the practical application of “co-” terms varied. Some studies integrated CBPR and co-development throughout the research process (e.g., Gewirtz, Cawley), while others used these approaches only at certain stages, such as in curriculum co-development [53,56] or during feedback sessions [51,52,58]. Reliance on the study terminology alone was insufficient—evaluating both the stage and the depth of CFEH involvement was essential. Therefore, to address any nuances and inconsistencies in language and documentation, we compared studies against benchmark criteria [26,28] as shown in Table 3.

Using a dual-analytic approach, we assessed participation by the stage at which families were involved and the depth of their involvement in the research and intervention development, ranging from “Engage” to “Co-produce.” All studies involved CFEH at least in “defining and engaging the community,” but only some demonstrated sustained collaboration, consensus-building, and shared decision-making through to the dissemination stage. Although only one study [50] used the term “reciprocity,” other studies exemplified elements of reciprocity that extended beyond consultation. These included having influence over decisions, engaging in iterative feedback loops that shared roles, and actively shaping the intervention. Gewirtz O’Brien et al. [53] and Williams-Arya et al. [58] provided evidence of family participation across all major stages, including data analysis and interpretation of community needs assessments.

Operationalizing participatory approaches presented several challenges. Some studies noted difficulties concerning logistics, sample representativeness [53,56,58], participant retention [51,57], possible competing priorities among partners [50,56,57], and the measurement of group-level outcomes [52,54,55,58]. External factors, such as the pandemic, also hindered engagement [50,57]. Some interventions self-labeled as “co-produced” lacked clear documentation of roles and power-sharing, risking overstatement of collaboration and complicating potential reproducibility. For example, some studies involved CFEH in dissemination activities, but it was unclear whether they had decision-making authority to determine them.

The range of CFEH involvement—from consultation to co-production—illustrated both strengths and challenges: engagement ensured interventions benefited from lived experience and promoted transparency, while full co-production (seen in only two studies [53,55]) required significant time, trust, and resources. The most effective interventions included both CFEH and service providers, clearly defined partner roles, emphasized lived experience, and fostered consensus, highlighting the value of ongoing, meaningful CFEH participation in developing homelessness solutions. More specific examples are in the following Section 3.4.

### 3.4. Intervention Types

Interventions described in the extracted articles were organized thematically by intervention type: socio-structural, behavioral, or a combination (i.e., behavioral and socio-structural). The final articles included were multi-disciplinary, ranging from public health to social work, policy, the arts, and more. These interventions often had secondary objectives (e.g., housing stability, public awareness) that extended beyond the traditional boundaries of the public health sector. Most studies reported improved overall well-being, resilience, mental skills development, and identification of support systems, as well as process-based outcomes [47,50,51,54,55]. See Table 4 for a summary of the study intervention types and main outcomes.

#### 3.4.1. Socio-Structural Interventions

Socio-structural interventions aimed to improve health outcomes by changing the underlying environmental contexts (i.e., political, educational, social, or economic). Two included articles shared a focus on combating stigma, increasing public awareness and engagement, and advocating for policy change [47,51]. These interventions consequently enhanced confidence, social inclusion, and equity among CFEH.

Science Together was designed for families with babies and “K-5-aged children” (~5–11 yrs old) to combat stigma; this education program was co-created by a science center, a temporary homeless shelter, and CFEH [50]. The intervention’s iterative co-development process and power-sharing, involving museum staff, shelter staff, and community partners, including parents, sought to create programs focused on interests and engagement rather than just educational content. The authors thoughtfully considered their positionality of privilege and valued lived experience, which required active listening. Such considerations helped successfully engage the participants while also working to address social stigma surrounding homelessness and its subsequent toll on mental health and well-being [50]. Cawley et al. (2022) [50] was similar to the following combination interventions [51,57] in utilizing iterative feedback mechanisms and reflective practices to adapt programs as needed. This approach helped maintain continued relevance for a highly mobile, transient population.

Likewise, Marziale et al. (2024) described positive engagement and several participatory collaborative arts and educational projects with and for migrant women in the U.K., which resulted in increased self-esteem and well-being [54]. The #NoticeUs campaign used art to highlight the challenges and needs of migrant women and CFEH living in temporary accommodation in the U.K. Such art depicted their requests to the local council (i.e., fair eviction notices and Wi-Fi). As a result, the group co-designed a survey exploring Wi-Fi needs among migrants living in TA, and a subsequent pilot was launched, with SIM cards provided to those residing in TA to enable Wi-Fi access [54]. To share their experiences of poor housing conditions and hostile environments in TA, the group also co-created an online theater performance, “The Shoe Shop,” to reach other migrant women experiencing homelessness, local policymakers, health professionals, teachers, artists, academics, and the public [54]. During an online session, participants visually mapped key issues they experienced in TA (e.g., poor mental health, inconvenient/distant relocation, and social isolation) [54], which provided rich qualitative data.

#### 3.4.2. Behavioral Interventions

Behavioral interventions, as described by Gewirtz O’Brien et al. (2022) and McKay et al. (2010) [53,55], on mental well-being and mental skill development among CFEH. Such interventions employed in-person, workshop-based programs led by family advocates to deliver mental health services for CFEH, as well as preventative sexual health support [53,55].

Gewirtz O’Brien et al. (2022) aimed to develop an evidence-informed intervention for pregnant women and parenting YEH in a transitional housing program using a trauma-informed approach [53]. Drawing on CBPR and implementation frameworks, the study involved youth, staff, and community experts at all stages, including guiding coding, triangulating needs assessment findings, and tailoring intervention components through consensus-building. YEH were involved in the implementation team and served on the “youth advisory board.” The selected intervention elements focused on an adaptable youth-driven program design to best engage youth and assist with on-site health care and access to primary care. As in Kerker et al. [57] and Cumming et al. [51], real-time adaptability was a key feature—partners stressed the importance of flexible program delivery to meet the dynamic needs of participants. Additionally, the vital role of housing staff and group leaders was emphasized. The Hexagon Exploration Tool aided in selecting and planning the “health empowerment program,” which prioritized a strengths-based approach similar to the My Strengths Training for Life^TM^ (MST4Life^TM^) program [51,52].

Similarly, McKay et al. had a “community collaborative board,” tasked with co-delivering the intervention program in family homeless shelters. McKay et al.’s Homeless Outreach for Parents and Early Adolescents (HOPE) program integrated evidence-based mental health services for pre- and early adolescents and their parents living in temporary shelters [55]. Parents served on the board to co-develop the program curriculum, which eventually engaged participants through games, visual aids, role-play, and other activities to help families discuss sensitive topics such as peer pressure, domestic violence, self-respect, and puberty [55]. Although the article included enough characteristics to be considered co-produced, it was limited by a lack of detailed documentation, including a step-by-step process for co-developing the curriculum and the representativeness of the board. This intervention has since been published in the literature [60], but it did not arise from our search strategy.

#### 3.4.3. Combination Interventions

Combination interventions, which constituted most of the studies, blended socio-structural and behavioral elements. These aimed to enhance resilience, educational continuity, and social support [51,52,56,57,58]. Four of the five clearly stated drawing on CBPR principles, and while one did not [57], it shared many key characteristics in following CBPR approaches [28].

Both Holtrop and Williams-Arya utilized CBPR and actively engaged CFEH in identifying key components for a parenting intervention [56,58]. However, in Holtrop, there was no indication of co-design with service staff or volunteers, a vital component of co-production. They incorporated CBPR into their study design by creating a “participatory experience” in which parents and caregivers voiced their needs to shape future interventions, rather than simply providing feedback after an intervention was delivered. However, the project was affected by CFEH’s competing priorities, and not all eligible families in the community wished to participate in the study. More specifically, Holtrop et al. [56] noted that families in transitional housing face external barriers to participation that may limit their ability to contribute during every stage of the research process, a challenge also experienced in other included studies [50,57].

Williams-Arya et al. created partnerships with the Family Housing Project (FHP) in Hamilton County, Ohio, which consisted of four Cincinnati family homeless shelters [58]. Participants (*n* = 53) were parents residing in the FHP. Williams-Arya et al. conducted five Group-Level Assessment (GLA) sessions, a seven-step, “collaborative and interactive” process, to collect qualitative data about the needs of CFEH. The GLA process incorporated consensus-building into action steps, as did Cawley and Gewirtz [50,53]. These led to tangible successes in the local community, as reflected in Marziale’s socio-structural outputs [54], such as the creation of the Cincinnati Solutions for Family Homelessness Children’s Task Force, which provided a foundation for their intervention strategies. Children’s programming was expanded in the shelters, including a Play & Learn early childhood development program for children ages 0–5, and improved school transportation was coordinated to ensure educational continuity. Lastly, parents were assisted in attending job and housing interviews through streamlining childcare referrals, thereby easing parents’ emotional burden related to childcare coordination. These subsequent interventions resulted in positive socio-emotional and educational outcomes for CFEH [58].

Kerker et al.’s iterative and complex Dynamic Adaptation Process (DAP) with the Exploration, Preparation, Implementation, and Sustainment (EPIS) model to tailor an intervention called Reach Out and Stay Strong, Essentials for New Mothers (ROSE) [57]. Across four DAP cycles, participant feedback was solicited at multiple stages and used to continually adapt the intervention to their needs [57]. Eligible participants were residents of a family shelter aged 18 years or older, did not have an untreated mental illness (including substance abuse), and were pregnant during the study; the criteria were later expanded to include mothers of children under 1 year of age. During the first DAP cycle, ROSE was adapted to Strong in Shelter (SIS) to relate more directly to the study population. In this case, the goal was to prevent postpartum depression through increasing social support and effective communication while decreasing stress for new or expectant mothers [57]. During DAP 1, participants reported high satisfaction with effectively requesting assistance and identifying a support system; they specifically stated the intervention helped address the stress of a new baby [1]. Notably, 58% or more of participants completed all sessions in each cycle, except for DAP 3, which occurred during the onset of the COVID-19 pandemic, when the intervention had to pivot to an online format [57]. During this unprecedented time, the research team did not account for technology literacy when shifting to the virtual format, which could have alienated some participants [57]. Furthermore, all materials were only available in English. Cawley et al. also adapted their approach during the pandemic, emphasizing the adoption of Universal Design Guidelines to ensure inclusivity [50]. Nevertheless, both studies valued community input and ensured that participants were engaged at every step in tailoring the interventions [57].

Cumming et al. reported on the MST4Life^TM^ program [51], which used youth development and basic psychological needs theory to reduce the risk of recurrent homelessness among youth (aged 16–24 years) residing in long-term supportive housing. Based on prior research demonstrating that YEH may have a more difficult time navigating hardship due to having faced adverse childhood experiences (ACEs), the researchers focused on developing a “strengths-based intervention based on *mental skills training (MST)*.” [51]. The pilot tested high overall feasibility and acceptability [52]. In the main study, facilitators used the Competence supportive, Autonomy supportive, Relatedness and interpersonal involvement, Engagement through communication, and Structure and group management (CARES) model for program delivery with fidelity measured by a rating scale tool consisting of 27 iteratively developed assessment items. Authors stated that findings demonstrated “high levels of needs supportive behaviors and low levels of needs thwarting behaviors,” but this was presented as a singular statement without further detailed, mixed-methods data to substantiate the interpretation [51]. According to Cumming et al., university staff with backgrounds in psychology and more extensive experience in program delivery demonstrated greater adherence to the preferred delivery style than frontline service staff from various backgrounds and with less program-delivery experience. Facilitators encountered barriers to delivering the intervention with fidelity, including high support needs, language barriers, participant drug use, and navigating issues related to communication with staff and the availability of suitable training spaces [51,52].

## 4. Discussion

To the best of our knowledge, this is the first review to assess the current literature on co-developed, community-based interventions with and for CFEH in HICs, as well as their impact on health and well-being outcomes. Prior to this systematic review, most systematic reviews on co-developed interventions have focused on adults experiencing homelessness or other extremely marginalized groups in HICs. After an exhaustive search, only nine articles met the inclusion criteria, which may help explain the scarcity of evidence. Despite these limitations, the findings offer valuable insights for future research, policy, and practice.

### 4.1. Limitations of the Evidence

#### 4.1.1. Study Quality and Characteristics

The existing literature is partially consistent with our evaluation of the nine studies [61,62,63]. These studies reflected limited geographical diversity and generally lacked rigor, as key quality assessment criteria could not be determined. All interventions were conducted in the U.S. and U.K. and also reflected the trend that limited research on parenting interventions has been primarily orchestrated in the U.S. Interestingly, these two HICs notably share the commonality of experiencing a growing housing crisis with elevated risk in these populations [11,34,64]. Notably, only six studies conducted needs assessments [50,51,53,56,57,58]—a key component of the intervention co-development process [65]. The nine studies included children across various age ranges, but only two specifically focused on under-5s [50,57]. Under-5s who experience homelessness are at higher risk of poor outcomes because the first 5 years of life are critical for optimal growth and cognitive development [66,67]. Therefore, the lack of focused evidence and emphasis on early childhood remains a substantial gap in the literature to be addressed.

Previous research has highlighted a deficiency of methodological rigor in evaluating the effectiveness and outcomes of co-produced interventions used to deliver acute health care services at the service and systems levels [61,62,68]. In this review, a common limitation across studies was the lack of comprehensive evaluation strategies and a reliance on anecdotal feedback, even in mixed-methods research, making it difficult to assess overall effectiveness and generalize outcomes. Even those relevant qualitative studies lacked a formal evaluation to demonstrate the intervention’s impact, which would still be expected of a co-produced intervention [69]. Non-qualitative studies often reported findings without statistical analyses that could have established internal and external validity [63], which, in turn, prevented both measurable impact and subsequent policy change. Most studies could not attribute observed improvements directly to the interventions because of the absence of control groups, pre-post measures, or adequate sample sizes, hindering causal inference. Analysis of the studies revealed that the timing and depth of family participation varied but often included early involvement in community engagement and needs assessment, followed by collaborative roles in program design, implementation, and, in some cases, dissemination.

#### 4.1.2. Definitional Differences

Differences in definitions across the literature posed considerable challenges and a lack of transparency. The interchangeable use of “co-” terms created ambiguity in distinguishing the methodologies actually employed and the level of CFEH’s involvement [17]. Similar challenges were identified in a recent scoping review, which found that 49 methods were derived from “co-” approaches and 11 from “community-based health promotion and action research” [68]. During screenings, we identified instances of lax terminology use, such as “peer-led,” “collaborative,” or “tailored”; however, the articles included no details regarding the depth of these approaches. Challenges arose further when studies were multidisciplinary, interdisciplinary, or transdisciplinary, and terms became convoluted, making it unclear which were correct or should be used.

Homelessness was defined differently across studies, complicating the assessment of their relevance to the inclusion criteria and comparability. No single universal definition of homelessness exists, and definitions often differ across settings, borders, and time periods, thus limiting generalizability and comparability [70]. For example, TA in each U.K. region [71] (e.g., England, Wales) differs from provisions in the U.S., where policies vary by local and state levels; for instance, New York City has the Right to Shelter, which does not apply statewide [72]. Some articles described participants as “vulnerable families,” “socially disadvantaged,” or “housing insecure,” without further defining these terms. In reality, these terms characterize populations in distinct ways. For example, the labels “houseless,” “unhoused,” and “housing insecure” specifically emphasize housing as the primary issue [73]. These terms can suggest that anyone with a roof over their head is *not homeless*. However, homelessness encompasses a complex, multifaceted array of experiences, involving numerous contributing factors and diverse forms of instability that extend beyond issues related to housing affordability. According to School House Connection, “housing insecurity” can downplay the harsh realities faced by families and youth living temporarily with others or in motels [73].

### 4.2. Strengths and Limitations

A key strength of this review was the rigor of the methods, including the use of databases across a range of disciplines to achieve broad disciplinary coverage. The search strategies were specifically designed to search for each concept broadly. In particular, the “co-development” concept was searched using terms related to the broader concepts of “participatory” and “action research.” Likewise, the strategy for the “homelessness” concept included terms for populations potentially “vulnerable” to “homelessness.” Notably, two out of the nine articles did not include the term “homeless” in their titles or abstracts but used categorical terms of homelessness instead (e.g., “temporary accommodation”) [54]. Additional studies focusing on important concepts in the research question (i.e., co-development and homelessness) were also searched as an extra way to mitigate issues related to the heterogeneity of terminology in the literature and the limitations of database searching. All texts were double reviewed in Covidence, with a third reviewer to check for conflicts and reach consensus [39]. Quality assessments were conducted in a similar fashion. The review was also registered on PROSPERO before the searches were conducted, and the review was guided by the PRISMA-Checklist for reporting [19].

This review had several limitations. Whereas some methods were employed to complement the database searches (e.g., web searches and consulting focused collections), supplementary retrieval methods, such as a full citation check or expert consultation, were not used. Overall, the research question concepts were challenging to search for because of the literature’s low consensus on terminology and the lack of relevant population details in abstracts. Some articles may have been unintentionally excluded (i.e., omission bias) because of these definitional differences and reliance on authors to include “co-” terminology or CBPR. A meta-analysis [74] could not be conducted because of the heterogeneity in study designs and outcomes, as well as the lack of reported information, including sample sizes, eligibility criteria, and statistical analyses.

There were also limitations with our assessment tools. Although the NHLBI study quality assessment tool [42] was initially selected for our systematic review for its emphasis on evidence-based interventions, it introduced additional limitations. The tool may have been too traditional or rigid in its intervention assessments and not sufficiently inclusive of all study designs, thereby not fully capturing the complexity and nuances inherent to co-developed, community-based research. The applicability of the NHLBI tool [42] was further restricted by the fact that some studies did not explicitly report their designs, had unique methodologies, and many were at various stages of intervention (i.e., development, implementation, or evaluation). This heterogeneity limited the relevance of specific assessment criteria. For instance, the tool may have overlooked qualitative insights or the unique strengths of participatory approaches, potentially underestimating their value. Additionally, several articles described multiphase studies at different stages of execution, making it impossible to apply specific criteria or to judge them on validated metrics without available results. The Mixed Methods Appraisal Tool (MMAT) [75] was also considered but had limitations similar to those of other tools and would have excluded three studies at the screening stage. It also does not fully address the stage of intervention development and levels of participant engagement. Thus, no single tool was ideal, so we selected NHLBI for its intervention focus and relevance to our research aim, while incorporating select MMAT criteria such as substantiated results and integration of study components by study design. Supplementary Table S6 indicate that using the MMAT would likely yield comparable assessment ratings.

Studies were not solely assessed using a checklist tool; we also mapped the methodologies against additional frameworks [26,28] to provide a more balanced assessment. However, these were also imperfect due to discrepancies or superficial reporting. For instance, CFEH may have been involved at a certain stage of a project but did not have equal power-sharing at that point, despite having such parity at other stages—this subtle distinction may not have been captured in the assessment and could be prone to bias. The study stages were compared with a CBPR-specific process; nevertheless, these approaches still exemplified standard good practices for collaborative work with communities and could be applied to any study, regardless of whether CBPR was used [28].

### 4.3. Implications for Future Research, Policy, and Practice

Co-production is essential to the inclusion health field, which seeks to address social and public health inequities [76] through effective, accessible services and policies designed to meet the needs of the most vulnerable and marginalized groups [77]. Co-production and co-development, although not novel, continue to evolve. The increasing prevalence of “co-” terms—such as “co-create,” “co-design,” “co-produce,” “co-deliver,” and “co-facilitate”—reflected a shift towards participatory and collaborative research methodologies that address critical issues affecting CFEH. Notably, seven out of nine studies were published within the past five years. These terms signified an effort to engage community members as equal partners throughout the research and intervention development process.

CBPR involved multi-step shared decision-making, mutual respect, and the acknowledgment of community expertise, thereby fostering interventions better aligned with lived experiences and community needs. However, dissimilar usage of “co-” terminology across the texts also created a significant barrier to accurately discerning both the authors’ intended meaning and their actual actions, hindering cross-study comparison and potentially misrepresenting CFEH involvement. A recent scoping review of “co”-terminology in chronic disease prevention found no substantive distinction in how the terms were used to describe interventions [17]. Developing a framework for “co-” terminology, in conjunction with enhanced search strategies that group related terms, would improve the visibility, comprehension, and impact of published studies. The Homeless Link’s principles can serve as a guide for co-producing a discipline-specific framework for “co-” terminology, emphasizing that *everyone* must be actively included (both CFEH and providers), particularly by prioritizing clear communication and by investigating all avenues of co-development [26].

#### 4.3.1. Key Takeaways

A notable and recurring strength identified across the studies was adaptability and strengths-based approaches, which were particularly pertinent for transient populations like CFEH. Despite differences in terminology, some approaches often drew on pragmatism, rooted in iterative, flexible processes, enabling interventions to be contextualized and continuously refined to meet the evolving needs of participants. CFEH participation spanned activities ranging from consultation to co-production, each associated with distinct advantages and challenges. The most effective projects demonstrated ongoing engagement and equal reciprocity with CFEH as co-researchers or decision-makers, often using consensus-building. Including CFEH in data analysis and interpretation likely improved the accuracy, relevance, and equity of the research findings. The greater level of CFEH involvement appeared to require significant time, trust, and resources. For instance, needs assessments and active listening were essential for understanding and empathizing with the community, accounting for logistical and external barriers to participation, and avoiding assumptions, especially regarding technological access and literacy levels. In turn, those who considered such barriers, as well as their own positionality, could improve buy-in and the effectiveness of an intervention.

#### 4.3.2. Challenges and Structural Constraints

Co-developed projects aimed at supporting CFEH often faced challenges due to differing partner priorities and varying definitions of success, underscoring the need to balance meaningful collaboration with reliable data collection and reproducibility. These studies also suggested that the current evidence base for CFEH remains limited by methodological weaknesses and unclear causality. Quality assessments indicated the need for a hybrid tool specifically tailored to this type of participatory literature, assessing both internal validity and the extent of CFEH involvement. This can help to better inform future practices and effective strategies for co-development studies in public health. Assessment criteria could be co-developed to incorporate additional research methodological categories that emphasize people-centered considerations, such as participant mobility, language digital literacy, resource constraints, cultural sensitivity, and timelines, ensuring evaluations are more inclusive and reflective of diverse participant needs and experiences. Regarding evaluations, applying the SMART (Specific, Measurable, Attainable, Relevant, Time-based) framework can help guide projects and define clear, measurable, and realistic goals, along with how to measure progress toward them [78]. Although a commonly used framework across sectors and in participatory research, its explicit application was noticeably absent in the reviewed literature.

#### 4.3.3. Future Research

Future research should prioritize the use of CBPR research tools documented in the scholarly literature and intended to facilitate community–academic research partnerships, strengthen research processes, and ultimately improve research outcomes [28,78,79]. To advance the field, future mixed-methods studies should also prioritize robust evaluations that balance both qualitative and quantitative data to assess the effectiveness and potential scalability of interventions. CBPR has been argued to improve the validity of research methods by potentially decreasing attrition and selection bias through including enhanced recruitment among marginalized and “hard-to-reach” populations [80,81]. However, this was not demonstrated in this review with the absence or ambiguity of sociodemographic data, particularly ethnicity.

Family homelessness disproportionately affects marginalized minority groups, and co-developed interventions are intended to be culturally relevant and grounded in lived experiences; therefore, in certain studies, the primary failure to adequately describe who they were serving was a significant ethical and methodological concern. This omission introduced bias and restricted the generalizability of the findings. Future research should carefully report sociodemographic data in culturally sensitive ways to demonstrate that affected minority groups have been both reached *and* included.

Although evidence specific to CFEH is still emerging, successful examples of well-designed, co-developed interventions have demonstrated impact among other extremely marginalized and vulnerable populations across different disciplines [17,18]. Fully “co-produced” services in the homeless and health sector are still rare or in their infancy [26]. Nevertheless, these examples can serve as exemplars for future projects. Investing time, building trust, and leveraging existing strengths—alongside creating opportunities, ongoing collaboration, and shared decision-making with CFEH—are essential for addressing systemic gaps and delivering high-impact interventions and services. Stepping towards full and more meaningful participation is not achieved by seeking feedback and reflection at a single stage, but by continuously working together and sharing power with CFEH as equal partners and relevant partners throughout the research cycle.

## 5. Conclusions

This review focused on co-developed interventions and their impact on the health and well-being of CFEH. Whereas some interventions showed promise, especially with process-based outcomes, limited details and varying stages of development made it difficult to comprehensively evaluate their overall impact. Inconsistent terminology and unclear levels of CFEH involvement in the studies posed additional challenges. To ensure genuine and effective interventions, future health initiatives should incorporate the lived experiences of CFEH *throughout* the process, since they are the experts on their own needs and situations. In multiple fields, co-developed research using CBPR approaches has previously shown success in fostering equitable partnerships and shared decision-making among vulnerable groups. Many studies included in the review were at different stages of implementation, providing insights into good practice at various points and suggesting the potential for substantial future evidence on the impact on health and well-being among CFEH.

## Figures and Tables

**Table 1:** Inclusion and Exclusion Criteria.

	Inclusion	Exclusion
**P**opulation	Children under 18 years old or families of such children	No children under 18 years old or families of such children Unable to distinguish this group from the broader population
	Experiencing homelessness: a lack of stable, safe, and adequate housing	Have not experienced homelessness Did not fit the McKinney-Vento definition [2] Unable to distinguish this group from the broader population
**I**ntervention	Intervention with health and community-based outcomes Intervention was co-developed (or other specified “co-” terms) with the population of interest or used community-based participatory research In high-income countries (HICs) [21]	No intervention Intervention is not health- or community-based Intervention was neither co-developed (or no other specified “co-” terms) with the population of interest nor employed community-based participatory research Not in HICs
**C**omparison	• No specific comparisons were made for this review	
**O**utcome	Any health or well-being outcome for children under 18 years old or families of such children Co-developed intervention	Any outcome that did not include health or well-being No co-developed intervention
Publication and study type	Peer-reviewed, original research, any study design	Not original research or peer reviewed (e.g., any review-type, editorials, comments) Conference abstracts and policy memos

**Table 2:** Study Characteristics.

Author	Year	Study Type	Country	Sample Size *n*	Setting	Population	Gender	Ethnicity
Cawley [50]	2022	Case Study (Qualitative)	U.S.	NR	Shelters	Babies and “K-5-aged children” (~5-11 years old) and their caregivers living in temporary shelter	NR	NR
Cumming [51]	2022	Mixed Methods	U.K.	195	Community	Current/former youth experiencing homelessness aged 16–24 years old in long-term supportive housing	NR	NR
Cumming [52]	2022	Mixed Methods	U.K.	15	Residential facility the during course	Current youth experiencing homelessness aged 16–24 years old in long-term supportive housing	Male 60% Female 40%	Black (e.g., African, Caribbean, or Black British) 46.7% White (e.g., British, Irish, Gypsy, Traveler, or other White backgrounds) 33.3% Mixed (i.e., multiple ethnic groups) 20%
Gewirtz O’Brien [53]	2022	Qualitative	U.S.	17 (31.2%: ages 16–17 yr.; 68.8%: 18–21 yr)	Shelters	Pregnant and parenting youth aged 16–21 years old experiencing homelessness	Female 76.5% Male 17.6% Trans/non-binary 5.9%	NR
Marziale [54]	2024	NR	U.K.	124	Shelters	Migrant women who have been in/are currently in temporary accommodation	Women 100%	NR
McKay [55]	2010	Randomized Clinical Trial	U.S.	208	Shelters	Early adolescents aged 11–14 years old and their families living in shelter	NR	NR
Holtrop [56]	2015	Qualitative	U.S.	40	Community	Parents and primary caregivers residing in transitional housing “typically” with their children	Woman 78% Male 22%	White 50.0% African American 47.5% Other 2.5%
Kerker [57]	2024	Mixed Methods	U.S.	88 total, but different n-values per cycle (DAP 1: 21 DAP 2: 31 DAP 3: 10 DAP 4: 26)	Shelters	Pregnant women and mothers with child(ren) age 1 year or younger residing in family shelter; one-third of participants were between the ages of 18 and 24, and half were between 25 and 34.	NR with socio-demographics, but all women by researchers’ identification	Black 47% Hispanic/Latina 41% Not specified 11%
Williams-Arya [58]	2021	Qualitative	U.S.	53	Shelters	Parents with children experiencing homelessness and residing in 1/4 shelters	Female 96% Not specified 4%	Consistent with demographic pool of Cincinnati family homeless shelter: African American 66% White 30% Multiracial 3% Other 1%

*Note. NR refers to data not reported.*

**Table 3:** Study Stages, Assessed Participation Levels, and Key Terminology Used.

Author	Study Stages *	Assessed Participation Levels †	Key Terminology Used ^∆^
Cawley [50]	Defining and Engaging the Community Community Needs Assessment Refine the Research Question Design and Methods Roles and Responsibilities Conduct Research Implementation Dissemination	Co-design: Longstanding partnership between shelter and community partners, including years of listening sessions with families Discussions and check-ins between educators and families related to “timing, format, content, and other material needs” for programming Iterative conception of central tenants and frameworks to account for transientness of shelter residents	“Co-creating” “Co-creation” “Co-developing” “Collaborative reflection” “Share power” “Reciprocity” “Consensus-building”
Cumming [51]	Defining and Engaging the Community Community Needs Assessment Design and Methods Roles and Responsibilities Conduct Research Dissemination	Co-design: Initial stakeholder consultation informed the need for an adaptable program; reflective practice (service users and facilitators) Focus groups and pilot sessions conducted with youth to assess needs for intervention Feedback and input collected through attendance records, field notes, and qualitative methods involving a semi-structured video diary, later replaced by an iteratively developed questionnaire and focus groups Ongoing needs assessment	“Co-developed” “Co-facilitated” “Community-based participatory research” “Collaborative and mutually beneficial process” “Competence supportive, Autonomy supportive, Relatedness and interpersonal involvement, Engagement through communication, and Structure and group management (CARES)”
Cumming [52]	Defining and Engaging the Community Design and Methods Conduct Research	Engage: Youth provided input and feedback through qualitative methods, including focus groups and diary-room entries	“Co-developed” “Co-produced” “Co-design” “Community-based participatory research” “Collaborative reflexivity” “Collaborative knowledge translation research” “Pragmatic stance”
Gewirtz O’Brien [53]	Defining and Engaging the Community Community Needs Assessment Design and Methods Roles and Responsibilities Conduct Research Analysis and Interpretation Dissemination	Co-produce: Community Needs Assessment included “essential elements of program content and design, drawing on constructs from the Consolidated Framework for Implementation Research” Youth were involved in processing needs assessment findings and tailoring intervention through consensus-building Youth served on community-engaged implementation team “Perspectives on Program Design Elements and Approach from Youth, Staff, and Community Experts” Drawing on the tenets of CBPR, they engaged youth, staff and community experts in every step of the process	“Co-facilitate” “Community-based participatory research” “Community-engaged process” “Consensus-building process” “Youth advisory board”
Marziale [54]	Defining and Engaging the Community Design and Methods Conduct Research Dissemination	Co-design: Families shared campaign and art advocacy efforts with the local council; co-designed a survey about Wi-Fi in TA, which led to action via pilot Qualitative data on issues experienced by families in temporary accommodation (TA) was shown via mapping Co-designed and co-delivered approximately twelve training sessions; however, no methodology was provided to elucidate the approach employed	“Co-production” “Co-produce” “Co-created” “Codesigned” “Co-delivered” “Participatory methods” “Collaborative approach” “Non-hierarchical and consensual decision-making” “Action-oriented framework” “Anti-racist intersectionality”
McKay [55]	Defining and Engaging the Community Design and Methods Roles and Responsibilities Conduct Research	Co-produce: Parents served on a collaborative board that worked to co-develop program curriculum; however, methodology was not reported in detail, describing the co-development of the intervention or representativeness of the board Community board members received training and support from clinically trained research staff to conduct the program’s family workshops	“Co-designed” “Co-delivered” “Collaboration” “Community collaborative board” “Collaborative planning group”
Holtrop [56]	Defining and Engaging the Community Community Needs Assessment Design and Methods Conduct Research	Co-design: Parents identified relevant components to include in the intervention based on their own needs and experiences in transitional housing; however, there is no indication that co-design was also with service staff or volunteers	“Community-based participatory research” “Participatory experience” “Collaboration”
Kerker [57]	Defining and Engaging the Community Community Needs Assessment Design and Methods Conduct Research Analysis and Interpretation Dissemination	Engage: Needs assessment included in Exploration stage Families provided input and feedback in four Dynamic Adaptation Process (DAP) cycles, tailoring the intervention at each stage based on their needs and shifting dynamics; During the first DAP, research staff held focus groups to determine acceptability of the intervention as well as readiness among participants	“Community engagement” “Engaging partners early and continually” “Iterative and flexible processes” “Acceptability” “Involve deep community participants’ input” “Facilitator” “Co-lead”
Williams-Arya [58]	Defining and Engaging the Community Community Needs Assessment Design and Methods Conduct Research Analysis and Interpretation Dissemination	Co-design: Participatory methodology—families performed a Group-Level Assessment (GLA) of their needs within shelter Families analyzed qualitative data and distilled themes Families’ recommendations led to action via local interventions	“Community-based participatory research” “Action-based participatory needs assessment” “Collaborative approach” “Interactive and collaborative process” “Participatory methodology” “Builds consensus toward action steps”

* “Study Stages” were determined using stages from CBPR scholar Karen Hacker [28]. These stages were used as indicators to determine when families were involved during the study. † “Assessed Participation Level” was defined by Stepping Stones, co-produced by Homeless Link and Expert Link [26]; these were used to assess how much CFEH were involved in the study (in order of increasing level of involvement of CFEH): inform, consult, engage, co-design, and co-produce. **^∆^** “Key Terminology Used” refers to the terms employed within the original manuscripts authored by the writers.

**Table 4:** Summary of Study Interventions.

Author	Year	Intervention Type	Main Outcomes
Cawley [50]	2022	Socio-Structural	Combated social stigma to minimize the subsequent toll on mental health and well-being Engaged CFEH
Cumming [51]	2022	Combination	Youth showed improved resilience, well-being, and mental skill development (e.g., self-regulation and interpersonal skills, autonomy-supportive strategies) Twice as likely to transition into education, employment, and/or training (EET) as compared to standard care Facilitators delivered the intervention with high fidelity Improved quality of life High levels of engagement (mean = 8.24; SD = 1.16) Participants’ positive reactions to the program; Interest/Enjoyment average score was 4.34 (SD = 0.82) based on range: 1 (not true at all) to 5 (very true)
Cumming [52]	2022	Combination	High levels of attendance ○ 75% for Phase 1: one session per week over 8 weeks ○ 100% for Phase 2: 4-day/3-night residential course Twice as likely to transition into education, employment, and/or training (EET) as compared to standard care High engagement with participants and positive reactions Facilitators delivered intervention with high fidelity Feasibility and acceptability of a positive youth development program
Gewirtz O’Brien [53]	2022	Behavioral	Community-engaged needs assessment Successful sexual and mental health-focused intervention development consisting of (1) a weekly health empowerment group, (2) health training and support for shelter staff, and (3) shelter-based health care services. ○ Five program content areas identified for group-based youth curriculum and for staff training: sexual health and relationships, mental health, child health and development, nutrition and exercise, and independent living skills.
Marziale [54]	2024	Socio-Structural	Positive engagement with participants Increased self-esteem and improved well-being with increased confidence, empowerment, and social inclusion Developed community-organizing and leadership skills, including better communication skills
McKay [55]	2010	Behavioral	Reduction in youth mental health symptoms Three out of 8 family sessions per HOPE (homeless outreach parents and early adolescents) met attendance goal of 60%
Holtrop [56]	2015	Combination	No intervention delivered yet Generated 15 components describing specific content areas (e.g., fostering parent–child communication) that could inform the curriculum of a parenting intervention for parents in transitional housing
Kerker [57]	2024	Combination	High-rated satisfaction surveys from participants in 3 of 4 Dynamic Adaptation Process (DAP) cycles Positive feedback on value and format of intervention (pre-COVID-19 shift to virtual format) Participants reported that the intervention helped with coping with the stress of a new baby, asking for help, and identifying support systems
Williams-Arya [58]	2021	Combination	Group-Level Assessment (GLA) informed later interventions Helped children maintain educational continuity Supported academic and socio-emotional skills development Promoted positive family interactions Facilitated childcare access

## Data Availability

No new data were created or analyzed in this study.

## Supplementary Materials

The following supporting information can be downloaded at: https://www.mdpi.com/article/10.3390/healthcare14040492/s1, Table S1: PRISMA Checklists; Table S2: Search Strategies; Table S3: Additional Records; Table S4: Data Extraction Sheets; Table S5: Study Definitions of Homelessness; Table S6: Study Quality Assessment Ratings and Study Limitations; Table S7: Quality Assessments.
