# Supplementary material for: Co-Developed Community-Based Health Interventions with Children Under 18 and Families Experiencing Homelessness in High-Income Countries: A Systematic Review
Source: Healthcare (Basel). 2026 Feb 14;14(4):492. doi: 10.3390/healthcare14040492 (PMC12940253; doi:10.3390/healthcare14040492)
Supplement: Supplementary file 1 [file healthcare-14-00492-s001.zip › healthcare-4070260-supplementary.pdf]

Supplementary Materials

Table S1. PRISMA Checklists

PRISMA 2020 Checklist

Section and Topic	Item #	Checklist item	Location where item is reported
**TITLE**			
Title	1	Identify the report as a systematic review.	p.1
**ABSTRACT**			
Abstract	2	See the PRISMA 2020 for Abstracts checklist.	p. 1 and See Abstract checklist below
**INTRODUCTION**			
Rationale	3	Describe the rationale for the review in the context of existing knowledge.	pp.2-3
Objectives	4	Provide an explicit statement of the objective(s) or question(s) the review addresses.	p. 3
**METHODS**			
Eligibility criteria	5	Specify the inclusion and exclusion criteria for the review and how studies were grouped for the syntheses.	pp.3-6
Information sources	6	Specify all databases, registers, websites, organisations, reference lists and other sources searched or consulted to identify studies. Specify the date when each source was last searched or consulted.	pp.4-5
Search strategy	7	Present the full search strategies for all databases, registers and websites, including any filters and limits used.	pp.4-5; **S2. Search Strategies, S3. Additional Records**
Selection process	8	Specify the methods used to decide whether a study met the inclusion criteria of the review, including how many reviewers screened each record and each report retrieved, whether they worked independently, and if applicable, details of automation tools used in the process.	pp. 5-6
Data collection process	9	Specify the methods used to collect data from reports, including how many reviewers collected data from each report, whether they worked independently, any processes for obtaining or confirming data from study investigators, and if applicable, details of automation tools used in the process.	p. 5-6
Data items	10a	List and define all outcomes for which data were sought. Specify whether all results that were compatible with each outcome domain in each study were sought (e.g. for all measures, time points, analyses), and if not, the methods used to decide which results to collect.	p. 5-6

Section and Topic	Item #	Checklist item	Location where item is reported
	10b	List and define all other variables for which data were sought (e.g. participant and intervention characteristics, funding sources). Describe any assumptions made about any missing or unclear information.	p. 5-6; **S4. Data Extraction Sheets**
Study risk of bias assessment	11	Specify the methods used to assess risk of bias in the included studies, including details of the tool(s) used, how many reviewers assessed each study and whether they worked independently, and if applicable, details of automation tools used in the process.	pp. 5-6
Effect measures	12	Specify for each outcome the effect measure(s) (e.g. risk ratio, mean difference) used in the synthesis or presentation of results.	p. 6
Synthesis methods	13a	Describe the processes used to decide which studies were eligible for each synthesis (e.g. tabulating the study intervention characteristics and comparing against the planned groups for each synthesis (item #5)).	pp. 3-6; **Table 1**
	13b	Describe any methods required to prepare the data for presentation or synthesis, such as handling of missing summary statistics, or data conversions.	NA
	13c	Describe any methods used to tabulate or visually display results of individual studies and syntheses.	p. 6
	13d	Describe any methods used to synthesize results and provide a rationale for the choice(s). If meta-analysis was performed, describe the model(s), method(s) to identify the presence and extent of statistical heterogeneity, and software package(s) used.	NA
	13e	Describe any methods used to explore possible causes of heterogeneity among study results (e.g. subgroup analysis, meta-regression).	NA
	13f	Describe any sensitivity analyses conducted to assess robustness of the synthesized results.	NA
Reporting bias assessment	14	Describe any methods used to assess risk of bias due to missing results in a synthesis (arising from reporting biases).	p.6; **Supplementary Materials S6 and S7.**
Certainty assessment	15	Describe any methods used to assess certainty (or confidence) in the body of evidence for an outcome.	NA
**RESULTS**			
Study selection	16a	Describe the results of the search and selection process, from the number of records identified in the search to the number of studies included in the review, ideally using a flow diagram.	pp. 7-8
	16b	Cite studies that might appear to meet the inclusion criteria, but which were excluded, and explain why they were excluded.	p. 8
Study characteristics	17	Cite each included study and present its characteristics.	pp. 8-19; **Tables 2-4**
Risk of bias in studies	18	Present assessments of risk of bias for each included study.	p. 10-15; **S6 and S7.**

Section and Topic	Item #	Checklist item	Location where item is reported
Results of individual studies	19	For all outcomes, present, for each study: (a) summary statistics for each group (where appropriate) and (b) an effect estimate and its precision (e.g. confidence/credible interval), ideally using structured tables or plots.	pp.10-19; **Table 4**
Results of syntheses	20a	For each synthesis, briefly summarise the characteristics and risk of bias among contributing studies.	pp.10-15; **S6 and S7.** and Discussion section
	20b	Present results of all statistical syntheses conducted. If meta-analysis was done, present for each the summary estimate and its precision (e.g. confidence/credible interval) and measures of statistical heterogeneity. If comparing groups, describe the direction of the effect.	NA
	20c	Present results of all investigations of possible causes of heterogeneity among study results.	p. 8-21; **S5-S7**
	20d	Present results of all sensitivity analyses conducted to assess the robustness of the synthesized results.	NA
Reporting biases	21	Present assessments of risk of bias due to missing results (arising from reporting biases) for each synthesis assessed.	p. 11-15; **S6 and S7.**
Certainty of evidence	22	Present assessments of certainty (or confidence) in the body of evidence for each outcome assessed.	p. 11-15; **S6 and S7.**
**DISCUSSION**			
Discussion	23a	Provide a general interpretation of the results in the context of other evidence.	pp. 21-22
	23b	Discuss any limitations of the evidence included in the review.	pp. 21-22
	23c	Discuss any limitations of the review processes used.	pp. 22-23
	23d	Discuss implications of the results for practice, policy, and future research.	pp.23-25
**OTHER INFORMATION**			
Registration and protocol	24a	Provide registration information for the review, including register name and registration number, or state that the review was not registered.	p. 3
	24b	Indicate where the review protocol can be accessed, or state that a protocol was not prepared.	All in supplementary materials
	24c	Describe and explain any amendments to information provided at registration or in the protocol.	NA
Support	25	Describe sources of financial or non-financial support for the review, and the role of the funders or sponsors in the review.	p. 25-26
Competing interests	26	Declare any competing interests of review authors.	pp. 25-26
Availability of data, code and other materials	27	Report which of the following are publicly available and where they can be found: template data collection forms; data extracted from included studies; data used for all analyses; analytic code; any other materials used in the review.	pp. 25-26 and anywhere supplementary materials referenced

From: Page MJ, McKenzie JE, Bossuyt PM, Boutron I, Hoffmann TC, Mulrow CD, et al. The PRISMA 2020 statement: an updated guideline for reporting systematic reviews. BMJ 2021;372:n71. doi: 10.1136/bmj.n71. This work is licensed under CC BY 4.0. To view a copy of this license, visit https://creativecommons.org/licenses/by/4.0/

PRISMA

PRISMA 2020 for Abstracts Checklist

Section and Topic	Item #	Checklist item	Reported (Yes/No)
**TITLE**			
Title	1	Identify the report as a systematic review.	Yes
**BACKGROUND**			
Objectives	2	Provide an explicit statement of the main objective(s) or question(s) the review addresses.	Yes
**METHODS**			
Eligibility criteria	3	Specify the inclusion and exclusion criteria for the review.	Yes
Information sources	4	Specify the information sources (e.g. databases, registers) used to identify studies and the date when each was last searched.	Yes
Risk of bias	5	Specify the methods used to assess risk of bias in the included studies.	Yes
Synthesis of results	6	Specify the methods used to present and synthesise results.	Yes
**RESULTS**			
Included studies	7	Give the total number of included studies and participants and summarise relevant characteristics of studies.	Yes
Synthesis of results	8	Present results for main outcomes, preferably indicating the number of included studies and participants for each. If meta-analysis was done, report the summary estimate and confidence/credible interval. If comparing groups, indicate the direction of the effect (i.e. which group is favoured).	Yes
**DISCUSSION**			
Limitations of evidence	9	Provide a brief summary of the limitations of the evidence included in the review (e.g. study risk of bias, inconsistency and imprecision).	Yes
Interpretation	10	Provide a general interpretation of the results and important implications.	Yes
**OTHER**			

Section and Topic	Item #	Checklist item	Reported (Yes/No)
Funding	11	Specify the primary source of funding for the review.	NA
Registration	12	Provide the register name and registration number.	No, because in the manuscript

From: Page MJ, McKenzie JE, Bossuyt PM, Boutron I, Hoffmann TC, Mulrow CD, et al. The PRISMA 2020 statement: an updated guideline for reporting systematic reviews. BMJ 2021;372:n71. doi: 10.1136/bmj.n71. This work is licensed under CC BY 4.0. To view a copy of this license, visit https://creativecommons.org/licenses/by/4.0/

Table S2. Search Strategies

Database Searches

Medline via PubMed
**Line 1 - Homelessness**
("displace*"[tiab] OR "evict*"[tiab] OR "food pantr*"[tiab] OR "homeless*"[tiab] OR "houseless*"[tiab] OR "housing condition*"[tiab] OR "housing first"[tiab] OR "housing insecur*"[tiab] OR "housing instability"[tiab] OR "housing problem*"[tiab] OR "housing quality"[tiab] OR "housing secur*"[tiab] OR "housing stabil*"[tiab] OR "ill housed"[tiab] OR "insecure accommodation*"[tiab] OR "insecure housing"[tiab] OR "insecurely housed"[tiab] OR "insufficient housing"[tiab] OR "migrant*"[tiab] OR "refugee*"[tiab] OR "roofless*"[tiab] OR "rough sleep*"[tiab] OR "runaway*"[tiab] OR "running away*"[tiab] OR "settlement*"[tiab] OR "shelter*"[tiab] OR "short term accommodation*"[tiab] OR "short term housing"[tiab] OR "slum*"[tiab] OR "sofa surf*"[tiab] OR "soup kitchen*"[tiab] OR "squatter*"[tiab] OR "squatting"[tiab] OR "street child*"[tiab] OR "street connected"[tiab] OR "street dwell*"[tiab] OR "street involved"[tiab] OR "street people"[tiab] OR "street youth"[tiab] OR "temporary accommodation*"[tiab] OR "temporary camp*"[tiab] OR "temporary housing"[tiab] OR "unhoused"[tiab] OR "unsheltered"[tiab] OR "unstable housing"[tiab] OR "unstably housed"[tiab] OR "residential mobility"[tiab] OR "farmworker*"[tiab] OR "farm worker*"[tiab] OR "resettlement*"[tiab] OR "Housing Quality"[Mesh] OR "Home Environment"[Mesh] OR "Housing Instability"[Mesh] OR "Ill-Housed Persons"[Mesh] OR "Refugee Camps"[Mesh] OR "Transients and Migrants"[Mesh])
**Line 2 - Co-development**
("action research*"[tiab] OR "citizen scien*"[tiab] OR "co-construct*"[tiab] OR "co-creat*"[tiab] OR "co-design*"[tiab] OR "co-develop*"[tiab] OR "co-produc*"[tiab] OR "co-research*"[tiab] OR "coconstruct*"[tiab] OR "cocreat*"[tiab] OR "codesign*"[tiab] OR "codevelop*"[tiab] OR "community driven"[tiab] OR "community engage*"[tiab] OR "community informed"[tiab] OR "community involve*"[tiab] OR "community led"[tiab] OR "community scien*"[tiab] OR "coproduc*"[tiab] OR "coresearch*"[tiab] OR "inclusive research"[tiab] OR "participative"[tiab] OR "participatory"[tiab] OR "partnership research"[tiab] OR "peer research*"[tiab] OR "public engage*"[tiab] OR "public involve*"[tiab] OR "public participation"[tiab] OR "stakeholder driven"[tiab] OR "stakeholder engage*"[tiab] OR "stakeholder involve*"[tiab] OR "stakeholder

led"[tiab] OR "youth driven"[tiab] OR "youth led"[tiab] OR "Community-Based Participatory Research"[Mesh] OR "Patient Participation"[Mesh] OR "Stakeholder Participation"[Mesh])
**Line 3 - Children/familie**s
("adolescen*"[tiab] OR "babies"[tiab] OR "baby"[tiab] OR "boy"[tiab] OR "boys"[tiab] OR "caregiver*"[tiab] OR "child"[tiab] OR "childhood"[tiab] OR "children"[tiab] OR "families"[tiab] OR "family"[tiab] OR "father"[tiab] OR "fathers"[tiab] OR "girl*"[tiab] OR "infant*"[tiab] OR "minors"[tiab] OR "mother"[tiab] OR "mothers"[tiab] OR "neonat*"[tiab] OR "newborn*"[tiab] OR "parent"[tiab] OR "parents"[tiab] OR "pediatri*"[tiab] OR "teen*"[tiab] OR "toddler*"[tiab] OR "youth"[tiab] OR "Adolescent"[Mesh] OR "Child, Preschool"[Mesh] OR "Child"[Mesh] OR “Family”[Mesh] OR "Infant"[Mesh] OR "Minors"[Mesh])
**Line 4 - Combine concepts**
#1 AND #2 AND #3
**Line 5 - Date, language, publication type**
(("2000/01/01"[Date - Publication] : "3000"[Date - Publication]) AND english[Language])<br>NOT<br>("comment"[Publication Type] OR "editorial"[Publication Type] OR "letter"[Publication Type])
**Line 6 - Limit by date, language; remove certain publication types**
#4 AND #5
**Line 7 - Low and middle income countries**
"afghan"[tw] OR "afghanistan"[tw] OR "albania"[tw] OR "albanian"[tw] OR "algeria"[tw] OR "algerian"[tw] OR "angola"[tw] OR "angolan"[tw] OR "argentina"[tw] OR "argentinian"[tw] OR "armenia"[tw] OR "armenian"[tw] OR "azerbaijan"[tw] OR "azerbaijani"[tw] OR "bajan"[tw] OR "bangladesh"[tw] OR "bangladeshi"[tw] OR "belarus"[tw] OR "belarusian"[tw] OR "belize"[tw] OR "belizean"[tw] OR "benin"[tw] OR "beninese"[tw] OR "bhutan"[tw] OR "bhutanese"[tw] OR "bolivia"[tw] OR "bolivian"[tw] OR "bosnia"[tw] OR "bosnian"[tw] OR "botswana"[tw] OR "brazil"[tw] OR "brazilian"[tw] OR "burkina faso"[tw] OR "burkinabes"[tw] OR "burmese"[tw] OR "burundi"[tw] OR "burundian"[tw] OR "cambodia"[tw] OR "cambodian"[tw] OR "cameroon"[tw] OR "cameroonian"[tw] OR "cape verde"[tw] OR "cape verdeans"[tw] OR "central african"[tw] OR "central african republic"[tw] OR "chad"[tw] OR "chadian"[tw] OR "china"[tw] OR "chinese"[tw] OR "colombia"[tw] OR "colombian"[tw] OR "comorian"[tw] OR

"comoros"[tw] OR "congo"[tw] OR "congolese"[tw] OR "costa rica"[tw] OR "costa rican"[tw] OR "cote d'ivoire"[tw] OR "cuba"[tw] OR "cuban"[tw] OR "djibouti"[tw] OR "dominica"[tw] OR "dominican"[tw] OR "ecuador"[tw] OR "ecuadorian"[tw] OR "egypt"[tw] OR "egyptian"[tw] OR "el salvador"[tw] OR "el salvadoran"[tw] OR "eritrea"[tw] OR "eritrean"[tw] OR "eswatini"[tw] OR "ethiopia"[tw] OR "ethiopian"[tw] OR "fiji"[tw] OR "fijian"[tw] OR "filipino"[tw] OR "gabon"[tw] OR "gabonese"[tw] OR "gambia"[tw] OR "gambian"[tw] OR "gaza"[tw] OR "ghana"[tw] OR "ghanaian"[tw] OR "grenada"[tw] OR "grenadian"[tw] OR "guatemala"[tw] OR "guatemalan"[tw] OR "guinea"[tw] OR "guinean"[tw] OR "haiti"[tw] OR "haitian"[tw] OR "honduran"[tw] OR "honduras"[tw] OR "india"[tw] OR "indian"[tw] OR "indonesia"[tw] OR "indonesian"[tw] OR "iran"[tw] OR "iranian"[tw] OR "iraq"[tw] OR "iraqi"[tw] OR "jamaica"[tw] OR "jamaica"[tw] OR "jamaican"[tw] OR "jamaican"[tw] OR "jordan"[tw] OR "jordan"[tw] OR "jordanian"[tw] OR "jordanian"[tw] OR "kazakhstan"[tw] OR "kazakhstan"[tw] OR "kenya"[tw] OR "kenya"[tw] OR "kenyan"[tw] OR "kenyan"[tw] OR "kiribati"[tw] OR "kiribati"[tw] OR "kosovar"[tw] OR "kosovar"[tw] OR "kosovo"[tw] OR "kosovo"[tw] OR "kyrgyz"[tw] OR "kyrgyz"[tw] OR "kyrgyzstan"[tw] OR "kyrgyzstan"[tw] OR "Lao"[tw] OR "Lao"[tw] OR "laos"[tw] OR "laos"[tw] OR "laotian"[tw] OR "laotian"[tw] OR "lebanese"[tw] OR "lebanese"[tw] OR "lebanon"[tw] OR "lebanon"[tw] OR "lesotho"[tw] OR "lesotho"[tw] OR "liberia"[tw] OR "liberia"[tw] OR "liberian"[tw] OR "liberian"[tw] OR "libya"[tw] OR "libya"[tw] OR "libyan"[tw] OR "libyan"[tw] OR "macedonia"[tw] OR "macedonia"[tw] OR "macedonian"[tw] OR "macedonian"[tw] OR "madagascar"[tw] OR "madagascar"[tw] OR "malagasy"[tw] OR "malagasy"[tw] OR "malawi"[tw] OR "malawi"[tw] OR "malawian"[tw] OR "malawian"[tw] OR "malaysia"[tw] OR "malaysia"[tw] OR "malaysian"[tw] OR "malaysian"[tw] OR "maldives"[tw] OR "maldives"[tw] OR "maldivian"[tw] OR "maldivian"[tw] OR "mali"[tw] OR "mali"[tw] OR "malian"[tw] OR "malian"[tw] OR "marshall islands"[tw] OR "marshall islands"[tw] OR "marshallese"[tw] OR "marshallese"[tw] OR "mauritania"[tw] OR "mauritania"[tw] OR "mauritanian"[tw] OR "mauritanian"[tw] OR "mauritian"[tw] OR "mauritian"[tw] OR "mauritius"[tw] OR "mauritius"[tw] OR "mexican"[tw] OR "mexican"[tw] OR "mexico"[tw] OR "mexico"[tw] OR "micronesia"[tw] OR "micronesia"[tw] OR "micronesian"[tw] OR "micronesian"[tw] OR "moldova"[tw] OR "moldova"[tw] OR "moldovan"[tw] OR "moldovan"[tw] OR "mongolia"[tw] OR "mongolia"[tw] OR "mongolian"[tw] OR "mongolian"[tw] OR "montenegrin"[tw] OR "montenegrin"[tw] OR "montenegro"[tw] OR "montenegro"[tw] OR "moroccan"[tw] OR "moroccan"[tw] OR "morocco"[tw] OR "morocco"[tw] OR "mozambican"[tw] OR "mozambican"[tw] OR "mozambique"[tw] OR "mozambique"[tw] OR "myanmar"[tw] OR "myanmar"[tw] OR "namibia"[tw] OR "namibian"[tw] OR "nepal"[tw] OR "nepalese"[tw] OR "nicaragua"[tw] OR "nicaraguan"[tw] OR "niger"[tw] OR "nigeria"[tw] OR "nigerian"[tw] OR "north korea"[tw] OR "north korea"[tw] OR "north korean"[tw] OR "north korean"[tw] OR "pakistan"[tw] OR "pakistani"[tw] OR "palau"[tw] OR "palauan"[tw] OR "panama"[tw] OR "panamanian"[tw] OR "papua new guinea"[tw] OR "papua new guinean"[tw] OR "paraguay"[tw] OR "paraguayan"[tw] OR "peru"[tw] OR "peruvian"[tw] OR "philippines"[tw] OR "principe"[tw] OR "romania"[tw] OR "romanian"[tw] OR "russia"[tw] OR "russian"[tw] OR "rwanda"[tw] OR "rwandan"[tw] OR "saint kitts"[tw] OR "saint lucia"[tw] OR "saint vincent"[tw] OR "salvadoran"[tw] OR "samoa"[tw] OR "samoan"[tw] OR "santomea"[tw] OR "sao tome"[tw] OR "senegal"[tw] OR "senegalese"[tw] OR "serbia"[tw] OR "serbian"[tw] OR "seychelles"[tw]

OR "seychellois"[tw] OR "sierra leone"[tw] OR "sierra leoneans"[tw] OR "solomon islander"[tw] OR "solomon islands"[tw] OR "somalia"[tw] OR "somalian"[tw] OR "south africa"[tw] OR "south african"[tw] OR "sri lanka"[tw] OR "sri lankan"[tw] OR "sudan"[tw] OR "sudanese"[tw] OR "suriname"[tw] OR "swazi"[tw] OR "swaziland"[tw] OR "syria"[tw] OR "syrian"[tw] OR "tadzhik"[tw] OR "tajik"[tw] OR "tajikistan"[tw] OR "tanzania"[tw] OR "tanzanian"[tw] OR "thai"[tw] OR "thailand"[tw] OR "timor"[tw] OR "togo"[tw] OR "togolese"[tw] OR "tonga"[tw] OR "tongan"[tw] OR "tunisia"[tw] OR "tunisian"[tw] OR "turkey"[tw] OR "turkish"[tw] OR "turkmen"[tw] OR "turkmenistan"[tw] OR "tuvalu"[tw] OR "tuvaluans"[tw] OR "uganda"[tw] OR "ugandan"[tw] OR "ukraine"[tw] OR "ukrainian"[tw] OR "uzbek"[tw] OR "uzbekistan"[tw] OR "vanuatu"[tw] OR "venezuela"[tw] OR "venezuelan"[tw] OR "vietnam"[tw] OR "vietnamese"[tw] OR "west bank"[tw] OR "yemen"[tw] OR "yemeni"[tw] OR "yemenite"[tw] OR "zambia"[tw] OR "zambian"[tw] OR "zimbabwe"[tw] OR "zimbabwean"[tw]
**Line 8 - High income countries**
"Alabama"[tw] OR "Alaska"[tw] OR "American Samoa"[tw] OR "Andorra"[tw] OR "Antigua and Barbuda"[tw] OR "Arizona"[tw] OR "Arkansas"[tw] OR "Aruba"[tw] OR "Australia"[tw] OR "Austria"[tw] OR "Bahamas"[tw] OR "Bahrain"[tw] OR "Barbados"[tw] OR "Belgium"[tw] OR "Bermuda"[tw] OR "Brunei"[tw] OR "Bulgaria"[tw] OR "California"[tw] OR "Canada"[tw] OR "Cayman Islands"[tw] OR "Channel Islands"[tw] OR "Chile"[tw] OR "Colorado"[tw] OR "Connecticut"[tw] OR "Croatia"[tw] OR "Curacao"[tw] OR "Cyprus"[tw] OR "Czech*"[tw] OR "Czechia"[tw] OR "Delaware"[tw] OR "Denmark"[tw] OR "District of Columbia"[tw] OR "england"[tw] OR "Estonia"[tw] OR "Faroe Islands"[tw] OR "Finland"[tw] OR "Florida"[tw] OR "France"[tw] OR "French Polynesia"[tw] OR "Georgia"[tw] OR "Germany"[tw] OR "Gibraltar"[tw] OR "Greece"[tw] OR "Greenland"[tw] OR "Guam"[tw] OR "Guyana"[tw] OR "Hawaii"[tw] OR "Hong Kong"[tw] OR "Hungary"[tw] OR "Iceland*"[tw] OR "Idaho*"[tw] OR "Illinois*"[tw] OR "Indiana*"[tw] OR "Iowa*"[tw] OR "Ireland"[tw] OR "Isle of Man"[tw] OR "Israel"[tw] OR "Italy"[tw] OR "Japan"[tw] OR "Kansas"[tw] OR "Kentucky"[tw] OR "Korea"[tw] OR "Kuwait"[tw] OR "Latvia"[tw] OR "Liechtenstein"[tw] OR "Lithuania"[tw] OR "Louisiana*"[tw] OR "Luxembourg"[tw] OR "Macao"[tw] OR "Maine"[tw] OR "Malta"[tw] OR "Maryland*"[tw] OR "Massachusetts"[tw] OR "Michigan"[tw] OR "Minnesota"[tw] OR "Mississippi"[tw] OR "Missouri"[tw] OR "Monaco"[tw] OR "Montana"[tw] OR "Nauru"[tw] OR "Nebraska"[tw] OR "Netherlands"[tw] OR "Nevada"[tw] OR "New Caledonia"[tw] OR "New Hampshire"[tw] OR "New Jersey"[tw] OR "New Mexico"[tw] OR "New York"[tw] OR "New Zealand"[tw] OR "North Carolina"[tw] OR "North Dakota"[tw] OR "Northern Mariana Islands"[tw] OR "Norway"[tw] OR "Ohio"[tw] OR "Oklahoma"[tw] OR "Oman"[tw] OR "Oregon"[tw] OR "Palau"[tw] OR "Panama"[tw] OR "Pennsylvania"[tw] OR "Poland"[tw] OR "Portugal"[tw] OR "Puerto Rico"[tw] OR "Qatar"[tw] OR "Rhode Island"[tw] OR "Romania"[tw] OR "Russia"[tw] OR "Russian"[tw] OR "Saint Kitts and Nevis"[tw] OR "Saint Martin"[tw] OR "San Marino"[tw] OR "Saudi Arabia"[tw] OR "scotland"[tw] OR "Seychelles"[tw] OR "Singapore"[tw] OR "Sint Maarten"[tw] OR "Slovak Republic"[tw] OR "Slovakia"[tw] OR "Slovenia"[tw] OR "South Carolina"[tw] OR "South Dakota"[tw] OR "Spain"[tw] OR "St Martin"[tw] OR "Sweden"[tw] OR "Switzerland"[tw]

OR "Taiwan"[tw] OR "Tennessee"[tw] OR "Texas"[tw] OR "Tobago"[tw] OR "Trinidad"[tw] OR "Turks and Caicos"[tw] OR "United Arab Emirates"[tw] OR "United Kingdom"[tw] OR "United States"[tw] OR "Uruguay"[tw] OR "Utah"[tw] OR "Vermont"[tw] OR "Virgin Islands"[tw] OR "Virginia"[tw] OR "wales"[tw] OR "Washington"[tw] OR "West Virginia"[tw] OR "Wisconsin"[tw] OR "Wyoming"[tw] OR "Alberta"[tw] OR "British Columbia"[tw] OR "Manitoba"[tw] OR "New Brunswick"[tw] OR "Newfoundland"[tw] OR "Northwest Territories"[tw] OR "Nova Scotia"[tw] OR "Nunavut"[tw] OR "Ontario"[tw] OR "Prince Edward Island"[tw] OR "Quebec"[tw] OR "Saskatchewan"[tw] OR "Yukon Territory"[tw] OR canada[mesh] OR "united states"[mesh] OR france[mesh] OR germany[mesh] OR russia[mesh] OR italy[mesh] OR denmark[mesh] OR norway[mesh] OR united kingdom[mesh] OR australia[mesh] OR "Republic of Korea"[Mesh]
**Line 9 - filter out LMICs unless they also mention HICs**
#6 NOT (#7 NOT #8)

CINAHL
**Line 1 - Homelessness**
((TI "displace*" OR AB "displace*") OR (TI "evict*" OR AB "evict*") OR (TI "food pantr*" OR AB "food pantr*") OR (TI "homeless*" OR AB "homeless*") OR (TI "houseless*" OR AB "houseless*") OR (TI "housing condition*" OR AB "housing condition*") OR (TI "housing first" OR AB "housing first") OR (TI "housing insecur*" OR AB "housing insecur*") OR (TI "housing instability" OR AB "housing instability") OR (TI "housing problem*" OR AB "housing problem*") OR (TI "housing quality" OR AB "housing quality") OR (TI "housing secur*" OR AB "housing secur*") OR (TI "housing stabil*" OR AB "housing stabil*") OR (TI "ill housed" OR AB "ill housed") OR (TI "insecure accommodation*" OR AB "insecure accommodation*") OR (TI "insecure housing" OR AB "insecure housing") OR (TI "insecurely housed" OR AB "insecurely housed") OR (TI "insufficient housing" OR AB "insufficient housing") OR (TI "migrant*" OR AB "migrant*") OR (TI "refugee*" OR AB "refugee*") OR (TI "roofless*" OR AB "roofless*") OR (TI "rough sleep*" OR AB "rough sleep*") OR (TI "runaway*" OR AB "runaway*") OR (TI "running away*" OR AB "running away*") OR (TI "settlement*" OR AB "settlement*") OR (TI "shelter*" OR AB "shelter*") OR (TI "short term accommodation*" OR AB "short term accommodation*") OR (TI "short term housing" OR AB "short term housing") OR (TI "slum*" OR AB "slum*") OR (TI "sofa surf*" OR AB "sofa surf*") OR (TI "soup kitchen*" OR AB "soup kitchen*") OR (TI "squatter*" OR AB "squatter*") OR (TI "squatting" OR AB "squatting") OR (TI "street child*" OR AB "street child*") OR (TI "street connected" OR AB "street connected") OR (TI "street dwell*" OR AB "street dwell*") OR (TI "street involved" OR AB "street involved") OR (TI "street

people" OR AB "street people") OR (TI "street youth" OR AB "street youth") OR (TI "temporary accommodation*" OR AB "temporary accommodation*") OR (TI "temporary camp*" OR AB "temporary camp*") OR (TI "temporary housing" OR AB "temporary housing") OR (TI "unhoused" OR AB "unhoused") OR (TI "unsheltered" OR AB "unsheltered") OR (TI "unstable housing" OR AB "unstable housing") OR (TI "unstably housed" OR AB "unstably housed") OR (TI "residential mobility" OR AB "residential mobility") OR (TI "farmworker*" OR AB "farmworker*") OR (TI "farm worker*" OR AB "farm worker*") OR (TI "resettlement*" OR AB "resettlement*") OR (MH "Farmworkers") OR (MH "Home Environment") OR (MH "Homeless Persons") OR (MH "Homelessness") OR (MH "Housing Instability") OR (MH "Migrants") OR (MH "Nomadic Persons+") OR (MH "Refugee Camps") OR (MH "Refugees") OR (MH "Residential Mobility") OR (MH "Runaways"))
**Line 2 - Co-development**
(TI "action research*" OR AB "action research*") OR (TI "citizen scien*" OR AB "citizen scien*") OR (TI "co-construct*" OR AB "co-construct*") OR (TI "co-creat*" OR AB "co-creat*") OR (TI "co-design*" OR AB "co-design*") OR (TI "co-develop*" OR AB "co-develop*") OR (TI "co-produc*" OR AB "co-produc*") OR (TI "co-research*" OR AB "co-research*") OR (TI "coconstruct*" OR AB "coconstruct*") OR (TI "cocreat*" OR AB "cocreat*") OR (TI "codesign*" OR AB "codesign*") OR (TI "codevelop*" OR AB "codevelop*") OR (TI "community driven" OR AB "community driven") OR (TI "community engage*" OR AB "community engage*") OR (TI "community informed" OR AB "community informed") OR (TI "community involve*" OR AB "community involve*") OR (TI "community led" OR AB "community led") OR (TI "community scien*" OR AB "community scien*") OR (TI "coproduc*" OR AB "coproduc*") OR (TI "coresearch*" OR AB "coresearch*") OR (TI "inclusive research" OR AB "inclusive research") OR (TI "participative" OR AB "participative") OR (TI "participatory" OR AB "participatory") OR (TI "partnership research" OR AB "partnership research") OR (TI "peer research*" OR AB "peer research*") OR (TI "public engage*" OR AB "public engage*") OR (TI "public involve*" OR AB "public involve*") OR (TI "public participation" OR AB "public participation") OR (TI "stakeholder driven" OR AB "stakeholder driven") OR (TI "stakeholder engage*" OR AB "stakeholder engage*") OR (TI "stakeholder involve*" OR AB "stakeholder involve*") OR (TI "stakeholder led" OR AB "stakeholder led") OR (TI "youth driven" OR AB "youth driven") OR (TI "youth led" OR AB "youth led") OR (MH "Action Research") OR (MH "Citizen Science") OR (MH "Patient Participation") OR (MH "Stakeholder Participation")
**Line 3 - Children/familie**s
((TI "adolescen*" OR AB "adolescen*") OR (TI "babies" OR AB "babies") OR (TI "baby" OR AB "baby") OR (TI "boy" OR AB "boy") OR (TI "boys" OR AB "boys") OR (TI "caregiver*" OR AB "caregiver*") OR (TI "child" OR AB "child") OR (TI "childhood" OR AB "childhood") OR (TI "children" OR AB "children") OR (TI "families" OR AB "families") OR (TI "family" OR AB "family") OR (TI "father" OR AB "father") OR (TI "fathers" OR AB "fathers") OR (TI "girl*" OR

AB "girl*") OR (TI "infant*" OR AB "infant*") OR (TI "minors" OR AB "minors") OR (TI "mother" OR AB "mother") OR (TI "mothers" OR AB "mothers") OR (TI "neonat*" OR AB "neonat*") OR (TI "newborn*" OR AB "newborn*") OR (TI "parent" OR AB "parent") OR (TI "parents" OR AB "parents") OR (TI "pediatri*" OR AB "pediatri*") OR (TI "teen*" OR AB "teen*") OR (TI "toddler*" OR AB "toddler*") OR (TI "youth" OR AB "youth") OR (MH "Adolescence+") OR (MH "Child+") OR (MH "Minors (Legal)") OR (MH "Family+") OR (MH "Dependent Families+"))
**Line 4 - Combine concepts**
S1 AND S2 AND S3
**Line 5 - Study type**
PT (abstract OR book OR "book chapter" OR "book review" OR commentary OR "doctoral dissertation" OR editorial OR masters thesis OR proceedings)
**Line 6 - Remove certain publication types**
S4 NOT S5
**Line 7 - Low and middle income countries**
(TI "afghan" OR AB "afghan" OR MW "afghan") OR (TI "afghanistan" OR AB "afghanistan" OR MW "afghanistan") OR (TI "albania" OR AB "albania" OR MW "albania") OR (TI "albanian" OR AB "albanian" OR MW "albanian") OR (TI "algeria" OR AB "algeria" OR MW "algeria") OR (TI "algerian" OR AB "algerian" OR MW "algerian") OR (TI "angola" OR AB "angola" OR MW "angola") OR (TI "angolan" OR AB "angolan" OR MW "angolan") OR (TI "argentina" OR AB "argentina" OR MW "argentina") OR (TI "argentinian" OR AB "argentinian" OR MW "argentinian") OR (TI "armenia" OR AB "armenia" OR MW "armenia") OR (TI "armenian" OR AB "armenian" OR MW "armenian") OR (TI "azerbaijan" OR AB "azerbaijan" OR MW "azerbaijan") OR (TI "azerbaijani" OR AB "azerbaijani" OR MW "azerbaijani") OR (TI "bajan" OR AB "bajan" OR MW "bajan") OR (TI "bangladesh" OR AB "bangladesh" OR MW "bangladesh") OR (TI "bangladeshi" OR AB "bangladeshi" OR MW "bangladeshi") OR (TI "belarus" OR AB "belarus" OR MW "belarus") OR (TI "belarusian" OR AB "belarusian" OR MW "belarusian") OR (TI "belize" OR AB "belize" OR MW "belize") OR (TI "belizean" OR AB "belizean" OR MW "belizean") OR (TI "benin" OR AB "benin" OR MW "benin") OR (TI "beninese" OR AB "beninese" OR MW "beninese") OR (TI "bhutan" OR AB "bhutan" OR MW "bhutan") OR (TI "bhutanese" OR AB "bhutanese" OR MW "bhutanese") OR (TI "bolivia" OR AB "bolivia" OR MW "bolivia") OR (TI "bolivian" OR AB "bolivian" OR MW "bolivian") OR (TI "bosnia" OR AB "bosnia" OR MW "bosnia") OR (TI "bosnian" OR AB "bosnian" OR MW "bosnian") OR (TI "botswana" OR AB "botswana" OR MW "botswana") OR (TI "brazil" OR AB

"brazil" OR MW "brazil") OR (TI "brazilian" OR AB "brazilian" OR MW "brazilian") OR (TI "burkina faso" OR AB "burkina faso" OR MW "burkina faso") OR (TI "burkinabes" OR AB "burkinabes" OR MW "burkinabes") OR (TI "burmese" OR AB "burmese" OR MW "burmese") OR (TI "burundi" OR AB "burundi" OR MW "burundi") OR (TI "burundian" OR AB "burundian" OR MW "burundian") OR (TI "cambodia" OR AB "cambodia" OR MW "cambodia") OR (TI "cambodian" OR AB "cambodian" OR MW "cambodian") OR (TI "cameroon" OR AB "cameroon" OR MW "cameroon") OR (TI "cameroonian" OR AB "cameroonian" OR MW "cameroonian") OR (TI "cape verde" OR AB "cape verde" OR MW "cape verde") OR (TI "cape verdeans" OR AB "cape verdeans" OR MW "cape verdeans") OR (TI "central african" OR AB "central african" OR MW "central african") OR (TI "central african republic" OR AB "central african republic" OR MW "central african republic") OR (TI "chad" OR AB "chad" OR MW "chad") OR (TI "chadian" OR AB "chadian" OR MW "chadian") OR (TI "china" OR AB "china" OR MW "china") OR (TI "chinese" OR AB "chinese" OR MW "chinese") OR (TI "colombia" OR AB "colombia" OR MW "colombia") OR (TI "colombian" OR AB "colombian" OR MW "colombian") OR (TI "comorian" OR AB "comorian" OR MW "comorian") OR (TI "comoros" OR AB "comoros" OR MW "comoros") OR (TI "congo" OR AB "congo" OR MW "congo") OR (TI "congolese" OR AB "congolese" OR MW "congolese") OR (TI "costa rica" OR AB "costa rica" OR MW "costa rica") OR (TI "costa rican" OR AB "costa rican" OR MW "costa rican") OR (TI "cote d'ivoire" OR AB "cote d'ivoire" OR MW "cote d'ivoire") OR (TI "cuba" OR AB "cuba" OR MW "cuba") OR (TI "cuban" OR AB "cuban" OR MW "cuban") OR (TI "djibouti" OR AB "djibouti" OR MW "djibouti") OR (TI "dominica" OR AB "dominica" OR MW "dominica") OR (TI "dominican" OR AB "dominican" OR MW "dominican") OR (TI "ecuador" OR AB "ecuador" OR MW "ecuador") OR (TI "ecuadorian" OR AB "ecuadorian" OR MW "ecuadorian") OR (TI "egypt" OR AB "egypt" OR MW "egypt") OR (TI "egyptian" OR AB "egyptian" OR MW "egyptian") OR (TI "el salvador" OR AB "el salvador" OR MW "el salvador") OR (TI "el salvadoran" OR AB "el salvadoran" OR MW "el salvadoran") OR (TI "eritrea" OR AB "eritrea" OR MW "eritrea") OR (TI "eritrean" OR AB "eritrean" OR MW "eritrean") OR (TI "eswatini" OR AB "eswatini" OR MW "eswatini") OR (TI "ethiopia" OR AB "ethiopia" OR MW "ethiopia") OR (TI "ethiopian" OR AB "ethiopian" OR MW "ethiopian") OR (TI "fiji" OR AB "fiji" OR MW "fiji") OR (TI "fijian" OR AB "fijian" OR MW "fijian") OR (TI "filipino" OR AB "filipino" OR MW "filipino") OR (TI "gabon" OR AB "gabon" OR MW "gabon") OR (TI "gabonese" OR AB "gabonese" OR MW "gabonese") OR (TI "gambia" OR AB "gambia" OR MW "gambia") OR (TI "gambian" OR AB "gambian" OR MW "gambian") OR (TI "gaza" OR AB "gaza" OR MW "gaza") OR (TI "ghana" OR AB "ghana" OR MW "ghana") OR (TI "ghanaian" OR AB "ghanaian" OR MW "ghanaian") OR (TI "grenada" OR AB "grenada" OR MW "grenada") OR (TI "grenadian" OR AB "grenadian" OR MW "grenadian") OR (TI "guatemala" OR AB "guatemala" OR MW "guatemala") OR (TI "guatemalan" OR AB "guatemalan" OR MW "guatemalan") OR (TI "guinea" OR AB "guinea" OR MW "guinea") OR (TI "guinean" OR AB "guinean" OR MW "guinean") OR (TI "haiti" OR AB "haiti" OR MW "haiti") OR (TI "haitian" OR AB "haitian" OR MW "haitian") OR (TI "honduran" OR AB "honduran" OR MW "honduran") OR (TI "honduras" OR AB "honduras" OR MW "honduras") OR (TI "india" OR AB "india" OR MW "india") OR (TI "indian" OR AB "indian" OR MW "indian") OR (TI "indonesia" OR AB "indonesia" OR MW "indonesia") OR (TI "indonesian" OR AB "indonesian" OR MW

"indonesian") OR (TI "iran" OR AB "iran" OR MW "iran") OR (TI "iranian" OR AB "iranian" OR MW "iranian") OR (TI "iraq" OR AB "iraq" OR MW "iraq") OR (TI "iraqi" OR AB "iraqi" OR MW "iraqi") OR (TI "jamaica" OR AB "jamaica" OR MW "jamaica") OR (TI "jamaica" OR AB "jamaica" OR MW "jamaica") OR (TI "jamaican" OR AB "jamaican" OR MW "jamaican") OR (TI "jamaican" OR AB "jamaican" OR MW "jamaican") OR (TI "jordan" OR AB "jordan" OR MW "jordan") OR (TI "jordan" OR AB "jordan" OR MW "jordan") OR (TI "jordanian" OR AB "jordanian" OR MW "jordanian") OR (TI "jordanian" OR AB "jordanian" OR MW "jordanian") OR (TI "kazakhstan" OR AB "kazakhstan" OR MW "kazakhstan") OR (TI "kazakhstan" OR AB "kazakhstan" OR MW "kazakhstan") OR (TI "kenya" OR AB "kenya" OR MW "kenya") OR (TI "kenya" OR AB "kenya" OR MW "kenya") OR (TI "kenyan" OR AB "kenyan" OR MW "kenyan") OR (TI "kenyan" OR AB "kenyan" OR MW "kenyan") OR (TI "kiribati" OR AB "kiribati" OR MW "kiribati") OR (TI "kiribati" OR AB "kiribati" OR MW "kiribati") OR (TI "kosovar" OR AB "kosovar" OR MW "kosovar") OR (TI "kosovar" OR AB "kosovar" OR MW "kosovar") OR (TI "kosovo" OR AB "kosovo" OR MW "kosovo") OR (TI "kosovo" OR AB "kosovo" OR MW "kosovo") OR (TI "kyrgyz" OR AB "kyrgyz" OR MW "kyrgyz") OR (TI "kyrgyz" OR AB "kyrgyz" OR MW "kyrgyz") OR (TI "kyrgyzstan" OR AB "kyrgyzstan" OR MW "kyrgyzstan") OR (TI "kyrgyzstan" OR AB "kyrgyzstan" OR MW "kyrgyzstan") OR (TI "Lao" OR AB "Lao" OR MW "Lao") OR (TI "Lao" OR AB "Lao" OR MW "Lao") OR (TI "laos" OR AB "laos" OR MW "laos") OR (TI "laos" OR AB "laos" OR MW "laos") OR (TI "laotian" OR AB "laotian" OR MW "laotian") OR (TI "laotian" OR AB "laotian" OR MW "laotian") OR (TI "lebanese" OR AB "lebanese" OR MW "lebanese") OR (TI "lebanese" OR AB "lebanese" OR MW "lebanese") OR (TI "lebanon" OR AB "lebanon" OR MW "lebanon") OR (TI "lebanon" OR AB "lebanon" OR MW "lebanon") OR (TI "lesotho" OR AB "lesotho" OR MW "lesotho") OR (TI "lesotho" OR AB "lesotho" OR MW "lesotho") OR (TI "liberia" OR AB "liberia" OR MW "liberia") OR (TI "liberia" OR AB "liberia" OR MW "liberia") OR (TI "liberian" OR AB "liberian" OR MW "liberian") OR (TI "liberian" OR AB "liberian" OR MW "liberian") OR (TI "libya" OR AB "libya" OR MW "libya") OR (TI "libya" OR AB "libya" OR MW "libya") OR (TI "libyan" OR AB "libyan" OR MW "libyan") OR (TI "libyan" OR AB "libyan" OR MW "libyan") OR (TI "macedonia" OR AB "macedonia" OR MW "macedonia") OR (TI "macedonia" OR AB "macedonia" OR MW "macedonia") OR (TI "macedonian" OR AB "macedonian" OR MW "macedonian") OR (TI "macedonian" OR AB "macedonian" OR MW "macedonian") OR (TI "madagascar" OR AB "madagascar" OR MW "madagascar") OR (TI "madagascar" OR AB "madagascar" OR MW "madagascar") OR (TI "malagasy" OR AB "malagasy" OR MW "malagasy") OR (TI "malagasy" OR AB "malagasy" OR MW "malagasy") OR (TI "malawi" OR AB "malawi" OR MW "malawi") OR (TI "malawi" OR AB "malawi" OR MW "malawi") OR (TI "malawian" OR AB "malawian" OR MW "malawian") OR (TI "malawian" OR AB "malawian" OR MW "malawian") OR (TI "malaysia" OR AB "malaysia" OR MW "malaysia") OR (TI "malaysia" OR AB "malaysia" OR MW "malaysia") OR (TI "malaysian" OR AB "malaysian" OR MW "malaysian") OR (TI "malaysian" OR AB "malaysian" OR MW "malaysian") OR (TI "maldives" OR AB "maldives" OR MW "maldives") OR (TI "maldives" OR AB "maldives" OR MW "maldives") OR (TI "maldivian" OR AB "maldivian" OR MW "maldivian") OR (TI "maldivian" OR AB "maldivian" OR MW "maldivian") OR (TI "mali" OR AB "mali" OR MW "mali") OR (TI "mali" OR AB "mali" OR MW "mali") OR (TI "malian" OR AB "malian" OR MW "malian") OR (TI "malian" OR AB "malian" OR MW "malian")

OR (TI "marshall islands" OR AB "marshall islands" OR MW "marshall islands") OR (TI "marshall islands" OR AB "marshall islands" OR MW "marshall islands") OR (TI "marshallese" OR AB "marshallese" OR MW "marshallese") OR (TI "marshallese" OR AB "marshallese" OR MW "marshallese") OR (TI "mauritania" OR AB "mauritania" OR MW "mauritania") OR (TI "mauritania" OR AB "mauritania" OR MW "mauritania") OR (TI "mauritanian" OR AB "mauritanian" OR MW "mauritanian") OR (TI "mauritanian" OR AB "mauritanian" OR MW "mauritanian") OR (TI "mauritian" OR AB "mauritian" OR MW "mauritian") OR (TI "mauritian" OR AB "mauritian" OR MW "mauritian") OR (TI "mauritius" OR AB "mauritius" OR MW "mauritius") OR (TI "mauritius" OR AB "mauritius" OR MW "mauritius") OR (TI "mexican" OR AB "mexican" OR MW "mexican") OR (TI "mexican" OR AB "mexican" OR MW "mexican") OR (TI "mexico" OR AB "mexico" OR MW "mexico") OR (TI "mexico" OR AB "mexico" OR MW "mexico") OR (TI "micronesia" OR AB "micronesia" OR MW "micronesia") OR (TI "micronesia" OR AB "micronesia" OR MW "micronesia") OR (TI "micronesian" OR AB "micronesian" OR MW "micronesian") OR (TI "micronesian" OR AB "micronesian" OR MW "micronesian") OR (TI "moldova" OR AB "moldova" OR MW "moldova") OR (TI "moldova" OR AB "moldova" OR MW "moldova") OR (TI "moldovan" OR AB "moldovan" OR MW "moldovan") OR (TI "moldovan" OR AB "moldovan" OR MW "moldovan") OR (TI "mongolia" OR AB "mongolia" OR MW "mongolia") OR (TI "mongolia" OR AB "mongolia" OR MW "mongolia") OR (TI "mongolian" OR AB "mongolian" OR MW "mongolian") OR (TI "mongolian" OR AB "mongolian" OR MW "mongolian") OR (TI "montenegrin" OR AB "montenegrin" OR MW "montenegrin") OR (TI "montenegrin" OR AB "montenegrin" OR MW "montenegrin") OR (TI "montenegro" OR AB "montenegro" OR MW "montenegro") OR (TI "montenegro" OR AB "montenegro" OR MW "montenegro") OR (TI "moroccan" OR AB "moroccan" OR MW "moroccan") OR (TI "moroccan" OR AB "moroccan" OR MW "moroccan") OR (TI "morocco" OR AB "morocco" OR MW "morocco") OR (TI "morocco" OR AB "morocco" OR MW "morocco") OR (TI "mozambican" OR AB "mozambican" OR MW "mozambican") OR (TI "mozambican" OR AB "mozambican" OR MW "mozambican") OR (TI "mozambique" OR AB "mozambique" OR MW "mozambique") OR (TI "mozambique" OR AB "mozambique" OR MW "mozambique") OR (TI "myanmar" OR AB "myanmar" OR MW "myanmar") OR (TI "myanmar" OR AB "myanmar" OR MW "myanmar") OR (TI "namibia" OR AB "namibia" OR MW "namibia") OR (TI "namibian" OR AB "namibian" OR MW "namibian") OR (TI "nepal" OR AB "nepal" OR MW "nepal") OR (TI "nepalese" OR AB "nepalese" OR MW "nepalese") OR (TI "nicaragua" OR AB "nicaragua" OR MW "nicaragua") OR (TI "nicaraguan" OR AB "nicaraguan" OR MW "nicaraguan") OR (TI "niger" OR AB "niger" OR MW "niger") OR (TI "nigeria" OR AB "nigeria" OR MW "nigeria") OR (TI "nigerian" OR AB "nigerian" OR MW "nigerian") OR (TI "north korea" OR AB "north korea" OR MW "north korea") OR (TI "north korea" OR AB "north korea" OR MW "north korea") OR (TI "north korean" OR AB "north korean" OR MW "north korean") OR (TI "north korean" OR AB "north korean" OR MW "north korean") OR (TI "pakistan" OR AB "pakistan" OR MW "pakistan") OR (TI "pakistani" OR AB "pakistani" OR MW "pakistani") OR (TI "palau" OR AB "palau" OR MW "palau") OR (TI "palauan" OR AB "palauan" OR MW "palauan") OR (TI "panama" OR AB "panama" OR MW "panama") OR (TI "panamanian" OR AB "panamanian" OR MW "panamanian") OR (TI "papua new guinea" OR AB "papua new guinea" OR MW "papua new guinea") OR (TI "papua new

guinean" OR AB "papua new guinean" OR MW "papua new guinean") OR (TI "paraguay" OR AB "paraguay" OR MW "paraguay") OR (TI "paraguayan" OR AB "paraguayan" OR MW "paraguayan") OR (TI "peru" OR AB "peru" OR MW "peru") OR (TI "peruvian" OR AB "peruvian" OR MW "peruvian") OR (TI "philippines" OR AB "philippines" OR MW "philippines") OR (TI "principe" OR AB "principe" OR MW "principe") OR (TI "romania" OR AB "romania" OR MW "romania") OR (TI "romanian" OR AB "romanian" OR MW "romanian") OR (TI "russia" OR AB "russia" OR MW "russia") OR (TI "russian" OR AB "russian" OR MW "russian") OR (TI "rwanda" OR AB "rwanda" OR MW "rwanda") OR (TI "rwandan" OR AB "rwandan" OR MW "rwandan") OR (TI "saint kitts" OR AB "saint kitts" OR MW "saint kitts") OR (TI "saint lucia" OR AB "saint lucia" OR MW "saint lucia") OR (TI "saint vincent" OR AB "saint vincent" OR MW "saint vincent") OR (TI "salvadoran" OR AB "salvadoran" OR MW "salvadoran") OR (TI "samoa" OR AB "samoa" OR MW "samoa") OR (TI "samoan" OR AB "samoan" OR MW "samoan") OR (TI "santomea" OR AB "santomea" OR MW "santomea") OR (TI "sao tome" OR AB "sao tome" OR MW "sao tome") OR (TI "senegal" OR AB "senegal" OR MW "senegal") OR (TI "senegalese" OR AB "senegalese" OR MW "senegalese") OR (TI "serbia" OR AB "serbia" OR MW "serbia") OR (TI "serbian" OR AB "serbian" OR MW "serbian") OR (TI "seychelles" OR AB "seychelles" OR MW "seychelles") OR (TI "seychellois" OR AB "seychellois" OR MW "seychellois") OR (TI "sierra leone" OR AB "sierra leone" OR MW "sierra leone") OR (TI "sierra leoneans" OR AB "sierra leoneans" OR MW "sierra leoneans") OR (TI "solomon islander" OR AB "solomon islander" OR MW "solomon islander") OR (TI "solomon islands" OR AB "solomon islands" OR MW "solomon islands") OR (TI "somalia" OR AB "somalia" OR MW "somalia") OR (TI "somalian" OR AB "somalian" OR MW "somalian") OR (TI "south africa" OR AB "south africa" OR MW "south africa") OR (TI "south african" OR AB "south african" OR MW "south african") OR (TI "sri lanka" OR AB "sri lanka" OR MW "sri lanka") OR (TI "sri lankan" OR AB "sri lankan" OR MW "sri lankan") OR (TI "sudan" OR AB "sudan" OR MW "sudan") OR (TI "sudanese" OR AB "sudanese" OR MW "sudanese") OR (TI "suriname" OR AB "suriname" OR MW "suriname") OR (TI "swazi" OR AB "swazi" OR MW "swazi") OR (TI "swaziland" OR AB "swaziland" OR MW "swaziland") OR (TI "syria" OR AB "syria" OR MW "syria") OR (TI "syrian" OR AB "syrian" OR MW "syrian") OR (TI "tadzhik" OR AB "tadzhik" OR MW "tadzhik") OR (TI "tajik" OR AB "tajik" OR MW "tajik") OR (TI "tajikistan" OR AB "tajikistan" OR MW "tajikistan") OR (TI "tanzania" OR AB "tanzania" OR MW "tanzania") OR (TI "tanzanian" OR AB "tanzanian" OR MW "tanzanian") OR (TI "thai" OR AB "thai" OR MW "thai") OR (TI "thailand" OR AB "thailand" OR MW "thailand") OR (TI "timor" OR AB "timor" OR MW "timor") OR (TI "togo" OR AB "togo" OR MW "togo") OR (TI "togolese" OR AB "togolese" OR MW "togolese") OR (TI "tonga" OR AB "tonga" OR MW "tonga") OR (TI "tongan" OR AB "tongan" OR MW "tongan") OR (TI "tunisia" OR AB "tunisia" OR MW "tunisia") OR (TI "tunisian" OR AB "tunisian" OR MW "tunisian") OR (TI "turkey" OR AB "turkey" OR MW "turkey") OR (TI "turkish" OR AB "turkish" OR MW "turkish") OR (TI "turkmen" OR AB "turkmen" OR MW "turkmen") OR (TI "turkmenistan" OR AB "turkmenistan" OR MW "turkmenistan") OR (TI "tuvalu" OR AB "tuvalu" OR MW "tuvalu") OR (TI "tuvaluans" OR AB "tuvaluans" OR MW "tuvaluans") OR (TI "uganda" OR AB "uganda" OR MW "uganda") OR (TI "ugandan" OR AB "ugandan" OR MW "ugandan") OR (TI "ukraine" OR AB "ukraine" OR MW "ukraine") OR (TI "ukrainian" OR AB "ukrainian" OR MW "ukrainian") OR (TI "uzbek"

OR AB "uzbek" OR MW "uzbek") OR (TI "uzbekistan" OR AB "uzbekistan" OR MW "uzbekistan") OR (TI "vanuatu" OR AB "vanuatu" OR MW "vanuatu") OR (TI "venezuela" OR AB "venezuela" OR MW "venezuela") OR (TI "venezuelan" OR AB "venezuelan" OR MW "venezuelan") OR (TI "vietnam" OR AB "vietnam" OR MW "vietnam") OR (TI "vietnamese" OR AB "vietnamese" OR MW "vietnamese") OR (TI "west bank" OR AB "west bank" OR MW "west bank") OR (TI "yemen" OR AB "yemen" OR MW "yemen") OR (TI "yemeni" OR AB "yemeni" OR MW "yemeni") OR (TI "yemenite" OR AB "yemenite" OR MW "yemenite") OR (TI "zambia" OR AB "zambia" OR MW "zambia") OR (TI "zambian" OR AB "zambian" OR MW "zambian") OR (TI "zimbabwe" OR AB "zimbabwe" OR MW "zimbabwe") OR (TI "zimbabwean" OR AB "zimbabwean" OR MW "zimbabwean")
**Line 8 - High income countries**
(TI "Alabama" OR AB "Alabama" OR MW "Alabama") OR (TI "Alaska" OR AB "Alaska" OR MW "Alaska") OR (TI "American Samoa" OR AB "American Samoa" OR MW "American Samoa") OR (TI "Andorra" OR AB "Andorra" OR MW "Andorra") OR (TI "Antigua and Barbuda" OR AB "Antigua and Barbuda" OR MW "Antigua and Barbuda") OR (TI "Arizona" OR AB "Arizona" OR MW "Arizona") OR (TI "Arkansas" OR AB "Arkansas" OR MW "Arkansas") OR (TI "Aruba" OR AB "Aruba" OR MW "Aruba") OR (TI "Australia" OR AB "Australia" OR MW "Australia") OR (TI "Austria" OR AB "Austria" OR MW "Austria") OR (TI "Bahamas" OR AB "Bahamas" OR MW "Bahamas") OR (TI "Bahrain" OR AB "Bahrain" OR MW "Bahrain") OR (TI "Barbados" OR AB "Barbados" OR MW "Barbados") OR (TI "Belgium" OR AB "Belgium" OR MW "Belgium") OR (TI "Bermuda" OR AB "Bermuda" OR MW "Bermuda") OR (TI "Brunei" OR AB "Brunei" OR MW "Brunei") OR (TI "Bulgaria" OR AB "Bulgaria" OR MW "Bulgaria") OR (TI "California" OR AB "California" OR MW "California") OR (TI "Canada" OR AB "Canada" OR MW "Canada") OR (TI "Cayman Islands" OR AB "Cayman Islands" OR MW "Cayman Islands") OR (TI "Channel Islands" OR AB "Channel Islands" OR MW "Channel Islands") OR (TI "Chile" OR AB "Chile" OR MW "Chile") OR (TI "Colorado" OR AB "Colorado" OR MW "Colorado") OR (TI "Connecticut" OR AB "Connecticut" OR MW "Connecticut") OR (TI "Croatia" OR AB "Croatia" OR MW "Croatia") OR (TI "Curacao" OR AB "Curacao" OR MW "Curacao") OR (TI "Cyprus" OR AB "Cyprus" OR MW "Cyprus") OR (TI "Czech*" OR AB "Czech*" OR MW "Czech*") OR (TI "Czechia" OR AB "Czechia" OR MW "Czechia") OR (TI "Delaware" OR AB "Delaware" OR MW "Delaware") OR (TI "Denmark" OR AB "Denmark" OR MW "Denmark") OR (TI "District of Columbia" OR AB "District of Columbia" OR MW "District of Columbia") OR (TI "england" OR AB "england" OR MW "england") OR (TI "Estonia" OR AB "Estonia" OR MW "Estonia") OR (TI "Faroe Islands" OR AB "Faroe Islands" OR MW "Faroe Islands") OR (TI "Finland" OR AB "Finland" OR MW "Finland") OR (TI "Florida" OR AB "Florida" OR MW "Florida") OR (TI "France" OR AB "France" OR MW "France") OR (TI "French Polynesia" OR AB "French Polynesia" OR MW "French Polynesia") OR (TI "Georgia" OR AB "Georgia" OR MW "Georgia") OR (TI "Germany" OR AB "Germany" OR MW "Germany") OR (TI "Gibraltar" OR AB "Gibraltar" OR MW "Gibraltar") OR (TI "Greece" OR AB "Greece" OR MW "Greece") OR (TI "Greenland" OR AB "Greenland" OR

MW "Greenland") OR (TI "Guam" OR AB "Guam" OR MW "Guam") OR (TI "Guyana" OR AB "Guyana" OR MW "Guyana") OR (TI "Hawaii" OR AB "Hawaii" OR MW "Hawaii") OR (TI "Hong Kong" OR AB "Hong Kong" OR MW "Hong Kong") OR (TI "Hungary" OR AB "Hungary" OR MW "Hungary") OR (TI "Iceland*" OR AB "Iceland*" OR MW "Iceland*") OR (TI "Idaho*" OR AB "Idaho*" OR MW "Idaho*") OR (TI "Illinois*" OR AB "Illinois*" OR MW "Illinois*") OR (TI "Indiana*" OR AB "Indiana*" OR MW "Indiana*") OR (TI "Iowa*" OR AB "Iowa*" OR MW "Iowa*") OR (TI "Ireland" OR AB "Ireland" OR MW "Ireland") OR (TI "Isle of Man" OR AB "Isle of Man" OR MW "Isle of Man") OR (TI "Israel" OR AB "Israel" OR MW "Israel") OR (TI "Italy" OR AB "Italy" OR MW "Italy") OR (TI "Japan" OR AB "Japan" OR MW "Japan") OR (TI "Kansas" OR AB "Kansas" OR MW "Kansas") OR (TI "Kentucky" OR AB "Kentucky" OR MW "Kentucky") OR (TI "Korea" OR AB "Korea" OR MW "Korea") OR (TI "Kuwait" OR AB "Kuwait" OR MW "Kuwait") OR (TI "Latvia" OR AB "Latvia" OR MW "Latvia") OR (TI "Liechtenstein" OR AB "Liechtenstein" OR MW "Liechtenstein") OR (TI "Lithuania" OR AB "Lithuania" OR MW "Lithuania") OR (TI "Louisiana*" OR AB "Louisiana*" OR MW "Louisiana*") OR (TI "Luxembourg" OR AB "Luxembourg" OR MW "Luxembourg") OR (TI "Macao" OR AB "Macao" OR MW "Macao") OR (TI "Maine" OR AB "Maine" OR MW "Maine") OR (TI "Malta" OR AB "Malta" OR MW "Malta") OR (TI "Maryland*" OR AB "Maryland*" OR MW "Maryland*") OR (TI "Massachusetts" OR AB "Massachusetts" OR MW "Massachusetts") OR (TI "Michigan" OR AB "Michigan" OR MW "Michigan") OR (TI "Minnesota" OR AB "Minnesota" OR MW "Minnesota") OR (TI "Mississippi" OR AB "Mississippi" OR MW "Mississippi") OR (TI "Missouri" OR AB "Missouri" OR MW "Missouri") OR (TI "Monaco" OR AB "Monaco" OR MW "Monaco") OR (TI "Montana" OR AB "Montana" OR MW "Montana") OR (TI "Nauru" OR AB "Nauru" OR MW "Nauru") OR (TI "Nebraska" OR AB "Nebraska" OR MW "Nebraska") OR (TI "Netherlands" OR AB "Netherlands" OR MW "Netherlands") OR (TI "Nevada" OR AB "Nevada" OR MW "Nevada") OR (TI "New Caledonia" OR AB "New Caledonia" OR MW "New Caledonia") OR (TI "New Hampshire" OR AB "New Hampshire" OR MW "New Hampshire") OR (TI "New Jersey" OR AB "New Jersey" OR MW "New Jersey") OR (TI "New Mexico" OR AB "New Mexico" OR MW "New Mexico") OR (TI "New York" OR AB "New York" OR MW "New York") OR (TI "New Zealand" OR AB "New Zealand" OR MW "New Zealand") OR (TI "North Carolina" OR AB "North Carolina" OR MW "North Carolina") OR (TI "North Dakota" OR AB "North Dakota" OR MW "North Dakota") OR (TI "Northern Mariana Islands" OR AB "Northern Mariana Islands" OR MW "Northern Mariana Islands") OR (TI "Norway" OR AB "Norway" OR MW "Norway") OR (TI "Ohio" OR AB "Ohio" OR MW "Ohio") OR (TI "Oklahoma" OR AB "Oklahoma" OR MW "Oklahoma") OR (TI "Oman" OR AB "Oman" OR MW "Oman") OR (TI "Oregon" OR AB "Oregon" OR MW "Oregon") OR (TI "Palau" OR AB "Palau" OR MW "Palau") OR (TI "Panama" OR AB "Panama" OR MW "Panama") OR (TI "Pennsylvania" OR AB "Pennsylvania" OR MW "Pennsylvania") OR (TI "Poland" OR AB "Poland" OR MW "Poland") OR (TI "Portugal" OR AB "Portugal" OR MW "Portugal") OR (TI "Puerto Rico" OR AB "Puerto Rico" OR MW "Puerto Rico") OR (TI "Qatar" OR AB "Qatar" OR MW "Qatar") OR (TI "Rhode Island" OR AB "Rhode Island" OR MW "Rhode Island") OR (TI "Romania" OR AB "Romania" OR MW "Romania") OR (TI "Russia" OR AB "Russia" OR MW "Russia") OR (TI "Russian" OR AB "Russian" OR MW "Russian") OR (TI "Saint Kitts and Nevis" OR AB "Saint Kitts and Nevis" OR MW "Saint Kitts and Nevis") OR (TI "Saint Martin" OR AB "Saint Martin"

OR MW "Saint Martin") OR (TI "San Marino" OR AB "San Marino" OR MW "San Marino") OR (TI "Saudi Arabia" OR AB "Saudi Arabia" OR MW "Saudi Arabia") OR (TI "scotland" OR AB "scotland" OR MW "scotland") OR (TI "Seychelles" OR AB "Seychelles" OR MW "Seychelles") OR (TI "Singapore" OR AB "Singapore" OR MW "Singapore") OR (TI "Sint Maarten" OR AB "Sint Maarten" OR MW "Sint Maarten") OR (TI "Slovak Republic" OR AB "Slovak Republic" OR MW "Slovak Republic") OR (TI "Slovakia" OR AB "Slovakia" OR MW "Slovakia") OR (TI "Slovenia" OR AB "Slovenia" OR MW "Slovenia") OR (TI "South Carolina" OR AB "South Carolina" OR MW "South Carolina") OR (TI "South Dakota" OR AB "South Dakota" OR MW "South Dakota") OR (TI "Spain" OR AB "Spain" OR MW "Spain") OR (TI "St Martin" OR AB "St Martin" OR MW "St Martin") OR (TI "Sweden" OR AB "Sweden" OR MW "Sweden") OR (TI "Switzerland" OR AB "Switzerland" OR MW "Switzerland") OR (TI "Taiwan" OR AB "Taiwan" OR MW "Taiwan") OR (TI "Tennessee" OR AB "Tennessee" OR MW "Tennessee") OR (TI "Texas" OR AB "Texas" OR MW "Texas") OR (TI "Tobago" OR AB "Tobago" OR MW "Tobago") OR (TI "Trinidad" OR AB "Trinidad" OR MW "Trinidad") OR (TI "Turks and Caicos" OR AB "Turks and Caicos" OR MW "Turks and Caicos") OR (TI "United Arab Emirates" OR AB "United Arab Emirates" OR MW "United Arab Emirates") OR (TI "United Kingdom" OR AB "United Kingdom" OR MW "United Kingdom") OR (TI "United States" OR AB "United States" OR MW "United States") OR (TI "Uruguay" OR AB "Uruguay" OR MW "Uruguay") OR (TI "Utah" OR AB "Utah" OR MW "Utah") OR (TI "Vermont" OR AB "Vermont" OR MW "Vermont") OR (TI "Virgin Islands" OR AB "Virgin Islands" OR MW "Virgin Islands") OR (TI "Virginia" OR AB "Virginia" OR MW "Virginia") OR (TI "wales" OR AB "wales" OR MW "wales") OR (TI "Washington" OR AB "Washington" OR MW "Washington") OR (TI "West Virginia" OR AB "West Virginia" OR MW "West Virginia") OR (TI "Wisconsin" OR AB "Wisconsin" OR MW "Wisconsin") OR (TI "Wyoming" OR AB "Wyoming" OR MW "Wyoming") OR (TI "Alberta" OR AB "Alberta" OR MW "Alberta") OR (TI "British Columbia" OR AB "British Columbia" OR MW "British Columbia") OR (TI "Manitoba" OR AB "Manitoba" OR MW "Manitoba") OR (TI "New Brunswick" OR AB "New Brunswick" OR MW "New Brunswick") OR (TI "Newfoundland" OR AB "Newfoundland" OR MW "Newfoundland") OR (TI "Northwest Territories" OR AB "Northwest Territories" OR MW "Northwest Territories") OR (TI "Nova Scotia" OR AB "Nova Scotia" OR MW "Nova Scotia") OR (TI "Nunavut" OR AB "Nunavut" OR MW "Nunavut") OR (TI "Ontario" OR AB "Ontario" OR MW "Ontario") OR (TI "Prince Edward Island" OR AB "Prince Edward Island" OR MW "Prince Edward Island") OR (TI "Quebec" OR AB "Quebec" OR MW "Quebec") OR (TI "Saskatchewan" OR AB "Saskatchewan" OR MW "Saskatchewan") OR (TI "Yukon Territory" OR AB "Yukon Territory" OR MW "Yukon Territory") OR (MH "Canada+") OR (MH "United States+") OR (MH "United Kingdom+") OR (MH "Australia+")
**Line 8 - filter out LMICs unless they also mention HICs**
S6 NOT (S7 NOT S8)
Used platform options to filter to:

Academic Journals<br>2000-2024<br>English

Embase
**Line 1 - Homelessness**
("displace*" or "encampment*" or "evict*" or "farm worker*" or "farmworker*" or "food pantr*" or "homeless*" or "houseless*" or "housing condition*" or "housing first" or "housing insecur*" or "housing instability" or "housing problem*" or "housing quality" or "housing secur*" or "housing stabil*" or "ill housed" or "insecure accommodation*" or "insecure housing" or "insecurely housed" or "insufficient housing" or "migrant*" or "refugee*" or "resettlement*" or "residential mobility" or "roofless*" or "rough sleep*" or "runaway*" or "running away*" or "settlement*" or "shelter*" or "short term accommodation*" or "short term housing" or "slum*" or "sofa surf*" or "soup kitchen*" or "squatter*" or "squatting" or "street child*" or "street connected" or "street dwell*" or "street involved" or "street people" or "street youth" or "temporary accommodation*" or "temporary camp*" or "temporary housing" or "unhoused" or "unsheltered" or "unstable housing" or "unstably housed").ti,ab. or exp homeless person/ or homelessness/ or agricultural worker/ or home environment/ or housing instability/ or exp housing quality/ or exp migrant/ or refugee camp/ or exp refugee/ or forced migration/ or runaway behavior/
**Line 2 - Co-development**
("action research*" or "citizen scien*" or "co-construct*" or "co-creat*" or "co-design*" or "co-develop*" or "co-produc*" or "co-research*" or "coconstruct*" or "cocreat*" or "codesign*" or "codevelop*" or "community driven" or "community engage*" or "community informed" or "community involve*" or "community led" or "community scien*" or "coproduc*" or "coresearch*" or "inclusive research" or "participative" or "participatory" or "partnership research" or "peer research*" or "public engage*" or "public involve*" or "public participation" or "stakeholder driven" or "stakeholder engage*" or "stakeholder involve*" or "stakeholder led" or "youth driven" or "youth led").ti,ab. or exp participatory research/ or exp community participation/
**Line 3 - Children/familie**s

("adolescen*" or "babies" or "baby" or "boy" or "boys" or "caregiver*" or "child" or "childhood" or "children" or "families" or "family" or "father" or "fathers" or "girl*" or "infant*" or "minors" or "mother" or "mothers" or "neonat*" or "newborn*" or "parent" or "parents" or "pediatri*" or "teen*" or "toddler*" or "youth").ti,ab. or exp adolescent/ or adolescent health/ or exp child/ or childhood/ or child health/ or juvenile/ or exp parent/ or exp family/
**Line 4 - Combine concepts**
1 AND 2 AND 3
**Line 6 - Low and middle income countries**
(afghan or afghanistan or albania or albanian or algeria or algerian or angola or angolan or argentina or argentinian or armenia or armenian or azerbaijan or azerbaijani or bajan or bangladesh or bangladeshi or belarus or belarusian or belize or belizean or benin or beninese or bhutan or bhutanese or bolivia or bolivian or bosnia or bosnian or botswana or brazil or brazilian or burkina faso or burkinabes or burmese or burundi or burundian or cambodia or cambodian or cameroon or cameroonian or cape verde or cape verdeans or central african or central african republic or chad or chadian or china or chinese or colombia or colombian or comorian or comoros or congo or congolese or costa rica or costa rican or cote d'ivoire or cuba or cuban or djibouti or dominica or dominican or ecuador or ecuadorian or egypt or egyptian or el salvador or el salvadoran or eritrea or eritrean or eswatini or ethiopia or ethiopian or fiji or fijian or filipino or gabon or gabonese or gambia or gambian or gaza or ghana or ghanaian or grenada or grenadian or guatemala or guatemalan or guinea or guinean or haiti or haitian or honduran or honduras or india or indian or indonesia or indonesian or iran or iranian or iraq or iraqi or jamaica or jamaica or jamaican or jamaican or jordan or jordan or jordanian or jordanian or kazakhstan or kazakhstan or kenya or kenya or kenyan or kenyan or kiribati or kiribati or kosovar or kosovar or kosovo or kosovo or kyrgyz or kyrgyz or kyrgyzstan or kyrgyzstan or Lao or Lao or laos or laos or laotian or laotian or lebanese or lebanese or lebanon or lebanon or lesotho or lesotho or liberia or liberia or liberian or liberian or libya or libya or libyan or libyan or macedonia or macedonia or macedonian or macedonian or madagascar or madagascar or malagasy or malagasy or malawi or malawi or malawian or malawian or malaysia or malaysia or malaysian or malaysian or maldives or maldives or maldivian or maldivian or mali or mali or malian or malian or marshall islands or marshall islands or marshallese or marshallese or mauritania or mauritania or mauritanian or mauritanian or mauritian or mauritian or mauritius or mauritius or mexican or mexican or mexico or mexico or micronesia or micronesia or micronesian or micronesian or moldova or moldova or moldovan or moldovan or mongolia or mongolia or mongolian or mongolian or montenegrin or montenegrin or montenegro or montenegro or moroccan or moroccan or morocco or morocco or mozambican or mozambican or mozambique or mozambique or myanmar or myanmar or namibia or namibian or nepal or nepalese or nicaragua or nicaraguan or niger or nigeria or nigerian or north korea or north korea or north korean or

north korean or pakistan or pakistani or palau or palauan or panama or panamanian or papua new guinea or papua new guinean or paraguay or paraguayan or peru or peruvian or philippines or principe or romania or romanian or russia or russian or rwanda or rwandan or saint kitts or saint lucia or saint vincent or salvadoran or samoa or samoan or santomea or sao tome or senegal or senegalese or serbia or serbian or seychelles or seychellois or sierra leone or sierra leoneans or solomon islander or solomon islands or somalia or somalian or south africa or south african or sri lanka or sri lankan or sudan or sudanese or suriname or swazi or swaziland or syria or syrian or tadzhik or tajik or tajikistan or tanzania or tanzanian or thai or thailand or timor or togo or togolese or tonga or tongan or tunisia or tunisian or turkey or turkish or turkmen or turkmenistan or tuvalu or tuvaluans or uganda or ugandan or ukraine or ukrainian or uzbek or uzbekistan or vanuatu or venezuela or venezuelan or vietnam or vietnamese or west bank or yemen or yemeni or yemenite or zambia or zambian or zimbabwe or zimbabwean).ti,ab,hw.
**Line 6 - High income countries**
(Alabama or Alaska or American Samoa or Andorra or "Antigua and Barbuda" or Arizona or Arkansas or Aruba or Australia or Austria or Bahamas or Bahrain or Barbados or Belgium or Bermuda or Brunei or Bulgaria or California or Canada or Cayman Islands or Channel Islands or Chile or Colorado or Connecticut or Croatia or Curacao or Cyprus or Czech* or Czechia or Delaware or Denmark or District of Columbia or england or Estonia or Faroe Islands or Finland or Florida or France or French Polynesia or Georgia or Germany or Gibraltar or Greece or Greenland or Guam or Guyana or Hawaii or Hong Kong or Hungary or Iceland* or Idaho* or Illinois* or Indiana* or Iowa* or Ireland or Isle of Man or Israel or Italy or Japan or Kansas or Kentucky or Korea or Kuwait or Latvia or Liechtenstein or Lithuania or Louisiana* or Luxembourg or Macao or Maine or Malta or Maryland* or Massachusetts or Michigan or Minnesota or Mississippi or Missouri or Monaco or Montana or Nauru or Nebraska or Netherlands or Nevada or New Caledonia or New Hampshire or New Jersey or New Mexico or New York or New Zealand or North Carolina or North Dakota or Northern Mariana Islands or Norway or Ohio or Oklahoma or Oman or Oregon or Palau or Panama or Pennsylvania or Poland or Portugal or Puerto Rico or Qatar or Rhode Island or Romania or Russia or Russian or "Saint Kitts and Nevis" or Saint Martin or San Marino or Saudi Arabia or scotland or Seychelles or Singapore or Sint Maarten or Slovak Republic or Slovakia or Slovenia or South Carolina or South Dakota or Spain or St Martin or Sweden or Switzerland or Taiwan or Tennessee or Texas or Tobago or Trinidad or "Turks and Caicos" or United Arab Emirates or United Kingdom or United States or Uruguay or Utah or Vermont or Virgin Islands or Virginia or wales or Washington or West Virginia or Wisconsin or Wyoming or Alberta or British Columbia or Manitoba or New Brunswick or Newfoundland or Northwest Territories or Nova Scotia or Nunavut or Ontario or Prince Edward Island or Quebec or Saskatchewan or Yukon Territory).ti,ab,hw.

**Line 7 - filter out LMICs unless they also mention HICs**
4 not (5 not 6)
**Line 8 - limit by language, year, study type**
limit 7 to (english language and yr="2000 -Current" and (article or article in press or letter or "review"))

Global Health
**Line 1 - Homelessness**
(adolescen* or babies or baby or boy or boys or caregiver* or child or childhood or children or families or family or father or fathers or girl* or infant* or minors or mother or mothers or neonat* or newborn* or parent or parents or pediatri* or teen* or toddler* or youth).ti,ab,hw.
**Line 2 - Co-development**
(action research* or citizen scien* or co-construct* or co-creat* or co-design* or co-develop* or co-produc* or co-research* or coconstruct* or cocreat* or codesign* or codevelop* or community driven or community engage* or community informed or community involve* or community led or community scien* or coproduc* or coresearch* or inclusive research or participative or participatory or partnership research or peer research* or public engage* or public involve* or public participation or stakeholder driven or stakeholder engage* or stakeholder involve* or stakeholder led or youth driven or youth led).ti,ab,hw. or exp Community Involvement/
**Line 3 - Children/familie**s
(adolescen* or babies or baby or boy or boys or caregiver* or child or childhood or children or families or family or father or fathers or girl* or infant* or minors or mother or mothers or neonat* or newborn* or parent or parents or pediatri* or teen* or toddler* or youth).ti,ab,hw.
**Line 4 - Combine concepts**

1 AND 2 AND 3
**Line 5 - Low and middle income countries**
(afghan or afghanistan or albania or albanian or algeria or algerian or angola or angolan or argentina or argentinian or armenia or armenian or azerbaijan or azerbaijani or bajan or bangladesh or bangladeshi or belarus or belarusian or belize or belizean or benin or beninese or bhutan or bhutanese or bolivia or bolivian or bosnia or bosnian or botswana or brazil or brazilian or burkina faso or burkinabes or burmese or burundi or burundian or cambodia or cambodian or cameroon or cameroonian or cape verde or cape verdeans or central african or central african republic or chad or chadian or china or chinese or colombia or colombian or comorian or comoros or congo or congolese or costa rica or costa rican or cote d'ivoire or cuba or cuban or djibouti or dominica or dominican or ecuador or ecuadorian or egypt or egyptian or el salvador or el salvadoran or eritrea or eritrean or eswatini or ethiopia or ethiopian or fiji or fijian or filipino or gabon or gabonese or gambia or gambian or gaza or ghana or ghanaian or grenada or grenadian or guatemala or guatemalan or guinea or guinean or haiti or haitian or honduran or honduras or india or indian or indonesia or indonesian or iran or iranian or iraq or iraqi or jamaica or jamaica or jamaican or jamaican or jordan or jordan or jordanian or jordanian or kazakhstan or kazakhstan or kenya or kenya or kenyan or kenyan or kiribati or kiribati or kosovar or kosovar or kosovo or kosovo or kyrgyz or kyrgyz or kyrgyzstan or kyrgyzstan or Lao or Lao or laos or laos or laotian or laotian or lebanese or lebanese or lebanon or lebanon or lesotho or lesotho or liberia or liberia or liberian or liberian or libya or libya or libyan or libyan or macedonia or macedonia or macedonian or macedonian or madagascar or madagascar or malagasy or malagasy or malawi or malawi or malawian or malawian or malaysia or malaysia or malaysian or malaysian or maldives or maldives or maldivian or maldivian or mali or mali or malian or malian or marshall islands or marshall islands or marshallese or marshallese or mauritania or mauritania or mauritanian or mauritanian or mauritian or mauritian or mauritius or mauritius or mexican or mexican or mexico or mexico or micronesia or micronesia or micronesian or micronesian or moldova or moldova or moldovan or moldovan or mongolia or mongolia or mongolian or mongolian or montenegrin or montenegrin or montenegro or montenegro or moroccan or moroccan or morocco or morocco or mozambican or mozambican or mozambique or mozambique or myanmar or myanmar or namibia or namibian or nepal or nepalese or nicaragua or nicaraguan or niger or nigeria or nigerian or north korea or north korea or north korean or north korean or pakistan or pakistani or palau or palauan or panama or panamanian or papua new guinea or papua new guinean or paraguay or paraguayan or peru or peruvian or philippines or principe or romania or romanian or russia or russian or rwanda or rwandan or saint kitts or saint lucia or saint vincent or salvadoran or samoa or samoan or santomea or sao tome or senegal or senegalese or serbia or serbian or seychelles or seychellois or sierra leone or sierra leoneans or solomon islander or solomon islands or somalia or somalian or south africa or south african or sri lanka or sri lankan or sudan or sudanese or suriname or swazi or swaziland or syria or syrian or tadzhik or tajik or tajikistan or tanzania or tanzanian or

thai or thailand or timor or togo or togolese or tonga or tongan or tunisia or tunisian or turkey or turkish or turkmen or turkmenistan or tuvalu or tuvaluans or uganda or ugandan or ukraine or ukrainian or uzbek or uzbekistan or vanuatu or venezuela or venezuelan or vietnam or vietnamese or west bank or yemen or yemeni or yemenite or zambia or zambian or zimbabwe or zimbabwean).ti,ab,hw.
**Line 6 - High income countries**
(Alabama or Alaska or American Samoa or Andorra or "Antigua and Barbuda" or Arizona or Arkansas or Aruba or Australia or Austria or Bahamas or Bahrain or Barbados or Belgium or Bermuda or Brunei or Bulgaria or California or Canada or Cayman Islands or Channel Islands or Chile or Colorado or Connecticut or Croatia or Curacao or Cyprus or Czech* or Czechia or Delaware or Denmark or District of Columbia or england or Estonia or Faroe Islands or Finland or Florida or France or French Polynesia or Georgia or Germany or Gibraltar or Greece or Greenland or Guam or Guyana or Hawaii or Hong Kong or Hungary or Iceland* or Idaho* or Illinois* or Indiana* or Iowa* or Ireland or Isle of Man or Israel or Italy or Japan or Kansas or Kentucky or Korea or Kuwait or Latvia or Liechtenstein or Lithuania or Louisiana* or Luxembourg or Macao or Maine or Malta or Maryland* or Massachusetts or Michigan or Minnesota or Mississippi or Missouri or Monaco or Montana or Nauru or Nebraska or Netherlands or Nevada or New Caledonia or New Hampshire or New Jersey or New Mexico or New York or New Zealand or North Carolina or North Dakota or Northern Mariana Islands or Norway or Ohio or Oklahoma or Oman or Oregon or Palau or Panama or Pennsylvania or Poland or Portugal or Puerto Rico or Qatar or Rhode Island or Romania or Russia or Russian or "Saint Kitts and Nevis" or Saint Martin or San Marino or Saudi Arabia or scotland or Seychelles or Singapore or Sint Maarten or Slovak Republic or Slovakia or Slovenia or South Carolina or South Dakota or Spain or St Martin or Sweden or Switzerland or Taiwan or Tennessee or Texas or Tobago or Trinidad or "Turks and Caicos" or United Arab Emirates or United Kingdom or United States or Uruguay or Utah or Vermont or Virgin Islands or Virginia or wales or Washington or West Virginia or Wisconsin or Wyoming or Alberta or British Columbia or Manitoba or New Brunswick or Newfoundland or Northwest Territories or Nova Scotia or Nunavut or Ontario or Prince Edward Island or Quebec or Saskatchewan or Yukon Territory).ti,ab,hw.
**Line 7 - filter out LMICs unless they also mention HICs**
4 not (5 not 6)
**Line 8 - limit by language, date, study type**
limit 7 to (english language and yr="2000 -Current") and (conference paper or journal article)

PsycInfo via Ovid
**Line 1 - Homelessness**
(asylum seeker* or displace* or encampment* or evict* or farm worker* or farmworker* or food pantr* or homeless* or houseless* or housing condition* or housing first or housing insecur* or housing instability or housing problem* or housing quality or housing secur* or housing stabil* or ill housed or insecure accommodation* or insecure housing or insecurely housed or insufficient housing or migrant* or refugee* or resettlement* or residential mobility or roofless* or rough sleep* or runaway* or running away* or settlement* or shelter* or short term accommodation* or short term housing or slum* or sofa surf* or soup kitchen* or squatter* or squatting or street child* or street connected or street dwell* or street involved or street people or street youth or temporary accommodation* or temporary camp* or temporary housing or unhoused or unsheltered or unstable housing or unstably housed).ti,ab,hw. or exp migrant workers/ or human displacement/ or human migration/ or asylum seeking/ or runaway behavior/ or exp Homeless/ or exp shelters/
**Line 2 - Co-development**
(action research* or citizen scien* or co-construct* or co-creat* or co-design* or co-develop* or co-produc* or co-research* or coconstruct* or cocreat* or codesign* or codevelop* or community driven or community engage* or community informed or community involve* or community led or community scien* or coproduc* or coresearch* or inclusive research or participative or participatory or partnership research or peer research* or public engage* or public involve* or public participation or stakeholder driven or stakeholder engage* or stakeholder involve* or stakeholder led or youth driven or youth led).ti,ab,hw. or exp Action Research/ or exp Community-Based Participatory Research/ or exp Community Involvement/
**Line 3 - Children/familie**s
(adolescen* or babies or baby or boy or boys or caregiver* or child or childhood or children or families or family or father or fathers or girl* or infant* or minors or mother or mothers or neonat* or newborn* or parent or parents or pediatri* or teen* or toddler* or youth).ti,ab,hw.
**Line 4 - Combine concepts**
1 AND 2 AND 3
**Line 5 - Low and middle income countries**

(afghan or afghanistan or albania or albanian or algeria or algerian or angola or angolan or argentina or argentinian or armenia or armenian or azerbaijan or azerbaijani or bajan or bangladesh or bangladeshi or belarus or belarusian or belize or belizean or benin or beninese or bhutan or bhutanese or bolivia or bolivian or bosnia or bosnian or botswana or brazil or brazilian or burkina faso or burkinabes or burmese or burundi or burundian or cambodia or cambodian or cameroon or cameroonian or cape verde or cape verdeans or central african or central african republic or chad or chadian or china or chinese or colombia or colombian or comorian or comoros or congo or congolese or costa rica or costa rican or cote d'ivoire or cuba or cuban or djibouti or dominica or dominican or ecuador or ecuadorian or egypt or egyptian or el salvador or el salvadoran or eritrea or eritrean or eswatini or ethiopia or ethiopian or fiji or fijian or filipino or gabon or gabonese or gambia or gambian or gaza or ghana or ghanaian or grenada or grenadian or guatemala or guatemalan or guinea or guinean or haiti or haitian or honduran or honduras or india or indian or indonesia or indonesian or iran or iranian or iraq or iraqi or jamaica or jamaica or jamaican or jamaican or jordan or jordan or jordanian or jordanian or kazakhstan or kazakhstan or kenya or kenya or kenyan or kenyan or kiribati or kiribati or kosovar or kosovar or kosovo or kosovo or kyrgyz or kyrgyz or kyrgyzstan or kyrgyzstan or Lao or Lao or laos or laos or laotian or laotian or lebanese or lebanese or lebanon or lebanon or lesotho or lesotho or liberia or liberia or liberian or liberian or libya or libya or libyan or libyan or macedonia or macedonia or macedonian or macedonian or madagascar or madagascar or malagasy or malagasy or malawi or malawi or malawian or malawian or malaysia or malaysia or malaysian or malaysian or maldives or maldives or maldivian or maldivian or mali or mali or malian or malian or marshall islands or marshall islands or marshallese or marshallese or mauritania or mauritania or mauritanian or mauritanian or mauritian or mauritian or mauritius or mauritius or mexican or mexican or mexico or mexico or micronesia or micronesia or micronesian or micronesian or moldova or moldova or moldovan or moldovan or mongolia or mongolia or mongolian or mongolian or montenegrin or montenegrin or montenegro or montenegro or moroccan or moroccan or morocco or morocco or mozambican or mozambican or mozambique or mozambique or myanmar or myanmar or namibia or namibian or nepal or nepalese or nicaragua or nicaraguan or niger or nigeria or nigerian or north korea or north korea or north korean or north korean or pakistan or pakistani or palau or palauan or panama or panamanian or papua new guinea or papua new guinean or paraguay or paraguayan or peru or peruvian or philippines or principe or romania or romanian or russia or russian or rwanda or rwandan or saint kitts or saint lucia or saint vincent or salvadoran or samoa or samoan or santomea or sao tome or senegal or senegalese or serbia or serbian or seychelles or seychellois or sierra leone or sierra leoneans or solomon islander or solomon islands or somalia or somalian or south africa or south african or sri lanka or sri lankan or sudan or sudanese or suriname or swazi or swaziland or syria or syrian or tadzhik or tajik or tajikistan or tanzania or tanzanian or thai or thailand or timor or togo or togolese or tonga or tongan or tunisia or tunisian or turkey or turkish or turkmen or turkmenistan or tuvalu or tuvaluans or uganda or ugandan or ukraine or ukrainian or uzbek or uzbekistan or vanuatu or venezuela or venezuelan or vietnam or

vietnamese or west bank or yemen or yemeni or yemenite or zambia or zambian or zimbabwe or zimbabwean).ti,ab,lo
**Line 6 - High income countries**
(Alabama or Alaska or American Samoa or Andorra or "Antigua and Barbuda" or Arizona or Arkansas or Aruba or Australia or Austria or Bahamas or Bahrain or Barbados or Belgium or Bermuda or Brunei or Bulgaria or California or Canada or Cayman Islands or Channel Islands or Chile or Colorado or Connecticut or Croatia or Curacao or Cyprus or Czech* or Czechia or Delaware or Denmark or District of Columbia or england or Estonia or Faroe Islands or Finland or Florida or France or French Polynesia or Georgia or Germany or Gibraltar or Greece or Greenland or Guam or Guyana or Hawaii or Hong Kong or Hungary or Iceland* or Idaho* or Illinois* or Indiana* or Iowa* or Ireland or Isle of Man or Israel or Italy or Japan or Kansas or Kentucky or Korea or Kuwait or Latvia or Liechtenstein or Lithuania or Louisiana* or Luxembourg or Macao or Maine or Malta or Maryland* or Massachusetts or Michigan or Minnesota or Mississippi or Missouri or Monaco or Montana or Nauru or Nebraska or Netherlands or Nevada or New Caledonia or New Hampshire or New Jersey or New Mexico or New York or New Zealand or North Carolina or North Dakota or Northern Mariana Islands or Norway or Ohio or Oklahoma or Oman or Oregon or Palau or Panama or Pennsylvania or Poland or Portugal or Puerto Rico or Qatar or Rhode Island or Romania or Russia or Russian or "Saint Kitts and Nevis" or Saint Martin or San Marino or Saudi Arabia or scotland or Seychelles or Singapore or Sint Maarten or Slovak Republic or Slovakia or Slovenia or South Carolina or South Dakota or Spain or St Martin or Sweden or Switzerland or Taiwan or Tennessee or Texas or Tobago or Trinidad or "Turks and Caicos" or United Arab Emirates or United Kingdom or United States or Uruguay or Utah or Vermont or Virgin Islands or Virginia or wales or Washington or West Virginia or Wisconsin or Wyoming or Alberta or British Columbia or Manitoba or New Brunswick or Newfoundland or Northwest Territories or Nova Scotia or Nunavut or Ontario or Prince Edward Island or Quebec or Saskatchewan or Yukon Territory).ti,ab,lo
**Line 7 - filter out LMICs unless they also mention HICs**
4 not (5 not 6)
**Line 8 - Limit by language, dates**
limit 7 to (english language and yr="2000 -Current")
**Line 9 - Limit by publication type**

limit 8 to journal article

Eric/Education Source
**Line 1 - Homelessness**
((TI "displace*" OR AB "displace*") OR (TI "encampment*" OR AB "encampment*") OR (TI "evict*" OR AB "evict*") OR (TI "farm worker*" OR AB "farm worker*") OR (TI "farmworker*" OR AB "farmworker*") OR (TI "food pantr*" OR AB "food pantr*") OR (TI "homeless*" OR AB "homeless*") OR (TI "houseless*" OR AB "houseless*") OR (TI "housing condition*" OR AB "housing condition*") OR (TI "housing first" OR AB "housing first") OR (TI "housing insecur*" OR AB "housing insecur*") OR (TI "housing instability" OR AB "housing instability") OR (TI "housing problem*" OR AB "housing problem*") OR (TI "housing quality" OR AB "housing quality") OR (TI "housing secur*" OR AB "housing secur*") OR (TI "housing stabil*" OR AB "housing stabil*") OR (TI "ill housed" OR AB "ill housed") OR (TI "insecure accommodation*" OR AB "insecure accommodation*") OR (TI "insecure housing" OR AB "insecure housing") OR (TI "insecurely housed" OR AB "insecurely housed") OR (TI "insufficient housing" OR AB "insufficient housing") OR (TI "migrant*" OR AB "migrant*") OR (TI "refugee*" OR AB "refugee*") OR (TI "resettlement*" OR AB "resettlement*") OR (TI "residential mobility" OR AB "residential mobility") OR (TI "roofless*" OR AB "roofless*") OR (TI "rough sleep*" OR AB "rough sleep*") OR (TI "runaway*" OR AB "runaway*") OR (TI "running away*" OR AB "running away*") OR (TI "settlement*" OR AB "settlement*") OR (TI "shelter*" OR AB "shelter*") OR (TI "short term accommodation*" OR AB "short term accommodation*") OR (TI "short term housing" OR AB "short term housing") OR (TI "slum*" OR AB "slum*") OR (TI "sofa surf*" OR AB "sofa surf*") OR (TI "soup kitchen*" OR AB "soup kitchen*") OR (TI "squatter*" OR AB "squatter*") OR (TI "squatting" OR AB "squatting") OR (TI "street child*" OR AB "street child*") OR (TI "street connected" OR AB "street connected") OR (TI "street dwell*" OR AB "street dwell*") OR (TI "street involved" OR AB "street involved") OR (TI "street people" OR AB "street people") OR (TI "street youth" OR AB "street youth") OR (TI "temporary accommodation*" OR AB "temporary accommodation*") OR (TI "temporary camp*" OR AB "temporary camp*") OR (TI "temporary housing" OR AB "temporary housing") OR (TI "unhoused" OR AB "unhoused") OR (TI "unsheltered" OR AB "unsheltered") OR (TI "unstable housing" OR AB "unstable housing") OR (TI "unstably housed" OR AB "unstably housed") OR SU "Homeless People" OR SU "Emergency Shelters" OR SU "Housing Needs" OR SU "Homeless students" OR SU "Education of homeless children" OR SU "Homeless children")

**Line 2 - Co-development**
((TI "citizen scien*" OR AB "citizen scien*") OR (TI "co-construct*" OR AB "co-construct*") OR (TI "co-creat*" OR AB "co-creat*") OR (TI "co-design*" OR AB "co-design*") OR (TI "co-develop*" OR AB "co-develop*") OR (TI "co-produc*" OR AB "co-produc*") OR (TI "co-research*" OR AB "co-research*") OR (TI "coconstruct*" OR AB "coconstruct*") OR (TI "cocreat*" OR AB "cocreat*") OR (TI "codesign*" OR AB "codesign*") OR (TI "codevelop*" OR AB "codevelop*") OR (TI "community driven" OR AB "community driven") OR (TI "community engage*" OR AB "community engage*") OR (TI "community informed" OR AB "community informed") OR (TI "community involve*" OR AB "community involve*") OR (TI "community led" OR AB "community led") OR (TI "community scien*" OR AB "community scien*") OR (TI "coproduc*" OR AB "coproduc*") OR (TI "coresearch*" OR AB "coresearch*") OR (TI "inclusive research" OR AB "inclusive research") OR (TI "participative" OR AB "participative") OR (TI "participatory" OR AB "participatory") OR (TI "partnership research" OR AB "partnership research") OR (TI "peer research*" OR AB "peer research*") OR (TI "public engage*" OR AB "public engage*") OR (TI "public involve*" OR AB "public involve*") OR (TI "public participation" OR AB "public participation") OR (TI "stakeholder driven" OR AB "stakeholder driven") OR (TI "stakeholder engage*" OR AB "stakeholder engage*") OR (TI "stakeholder involve*" OR AB "stakeholder involve*") OR (TI "stakeholder led" OR AB "stakeholder led") OR (TI "youth driven" OR AB "youth driven") OR (TI "youth led" OR AB "youth led") OR SU "Action Research" OR SU "Participatory Research" OR SU "Action research" OR SU "Action research in education")
**Line 3 - Children/familie**s
((TI "adolescen*" OR AB "adolescen*") OR (TI "babies" OR AB "babies") OR (TI "baby" OR AB "baby") OR (TI "boy" OR AB "boy") OR (TI "boys" OR AB "boys") OR (TI "caregiver*" OR AB "caregiver*") OR (TI "child" OR AB "child") OR (TI "childhood" OR AB "childhood") OR (TI "children" OR AB "children") OR (TI "families" OR AB "families") OR (TI "family" OR AB "family") OR (TI "father" OR AB "father") OR (TI "fathers" OR AB "fathers") OR (TI "girl*" OR AB "girl*") OR (TI "infant*" OR AB "infant*") OR (TI "minors" OR AB "minors") OR (TI "mother" OR AB "mother") OR (TI "mothers" OR AB "mothers") OR (TI "neonat*" OR AB "neonat*") OR (TI "newborn*" OR AB "newborn*") OR (TI "parent" OR AB "parent") OR (TI "parents" OR AB "parents") OR (TI "pediatri*" OR AB "pediatri*") OR (TI "teen*" OR AB "teen*") OR (TI "toddler*" OR AB "toddler*") OR (TI "youth" OR AB "youth") OR SU "Children" OR SU "Youth" OR SU "Families" OR SU "Children" OR SU "Dependents" OR SU "Dysfunctional families" OR SU "Families of people with disabilities" OR SU "Family roles" OR SU "Family size" OR SU "Family structure" OR SU "Family support" OR SU "Family-school relationships" OR SU "Fatherless families" OR SU "Fathers" OR SU "Heads of households" OR SU "Host families of foreign students" OR SU "Language in families" OR SU "Motherless families" OR SU

"Mothers" OR SU "Parent-child relationships" OR SU "Parents" OR SU "Siblings" OR SU "Single-parent families" OR SU "Stepfamilies" OR SU "Students' families")
**Line 4 - Health outcomes**
(TI "abus*" OR AB "abus*" OR SU "abus*") OR (TI "behavioral" OR AB "behavioral" OR SU "behavioral") OR (TI "behavioural" OR AB "behavioural" OR SU "behavioural") OR (TI "birth*" OR AB "birth*" OR SU "birth*") OR (TI "bodyweight" OR AB "bodyweight" OR SU "bodyweight") OR (TI "books" OR AB "books" OR SU "books") OR (TI "breast fe*" OR AB "breast fe*" OR SU "breast fe*") OR (TI "breast milk" OR AB "breast milk" OR SU "breast milk") OR (TI "breastfe*" OR AB "breastfe*" OR SU "breastfe*") OR (TI "breastmilk" OR AB "breastmilk" OR SU "breastmilk") OR (TI "caregiver-child" OR AB "caregiver-child" OR SU "caregiver-child") OR (TI "caregiving" OR AB "caregiving" OR SU "caregiving") OR (TI "child care" OR AB "child care" OR SU "child care") OR (TI "child development*" OR AB "child development*" OR SU "child development*") OR (TI "childbirth*" OR AB "childbirth*" OR SU "childbirth*") OR (TI "childcare" OR AB "childcare" OR SU "childcare") OR (TI "childhood" OR AB "childhood" OR SU "childhood") OR (TI "childhood development" OR AB "childhood development" OR SU "childhood development") OR (TI "children's development*" OR AB "children's development*" OR SU "children's development*") OR (TI "circumference" OR AB "circumference" OR SU "circumference") OR (TI "clean*" OR AB "clean*" OR SU "clean*") OR (TI "diet*" OR AB "diet*" OR SU "diet*") OR (TI "disciplin*" OR AB "disciplin*" OR SU "disciplin*") OR (TI "education" OR AB "education" OR SU "education") OR (TI "familial" OR AB "familial" OR SU "familial") OR (TI "father child" OR AB "father child" OR SU "father child") OR (TI "feeding" OR AB "feeding" OR SU "feeding") OR (TI "food*" OR AB "food*" OR SU "food*") OR (TI "gestation*" OR AB "gestation*" OR SU "gestation*") OR (TI "health*" OR AB "health*" OR SU "health*") OR (TI "height" OR AB "height" OR SU "height") OR (TI "hunger" OR AB "hunger" OR SU "hunger") OR (TI "hygien*" OR AB "hygien*" OR SU "hygien*") OR (TI "infant development*" OR AB "infant development*" OR SU "infant development*") OR (TI "lactat*" OR AB "lactat*" OR SU "lactat*") OR (TI "learning" OR AB "learning" OR SU "learning") OR (TI "literacy" OR AB "literacy" OR SU "literacy") OR (TI "malnutrition" OR AB "malnutrition" OR SU "malnutrition") OR (TI "maternal child" OR AB "maternal child" OR SU "maternal child") OR (TI "maternal infant" OR AB "maternal infant" OR SU "maternal infant") OR (TI "meal*" OR AB "meal*" OR SU "meal*") OR (TI "micronutrient*" OR AB "micronutrient*" OR SU "micronutrient*") OR (TI "mineral*" OR AB "mineral*" OR SU "mineral*") OR (TI "morbid*" OR AB "morbid*" OR SU "morbid*") OR (TI "mortal*" OR AB "mortal*" OR SU "mortal*") OR (TI "mother infant" OR AB "mother infant" OR SU "mother infant") OR (TI "neonatal development*" OR AB "neonatal development*" OR SU "neonatal development*") OR (TI "nutrient*" OR AB "nutrient*" OR SU "nutrient*") OR (TI "nutrition*" OR AB "nutrition*" OR SU "nutrition*") OR (TI "obes*" OR AB "obes*" OR SU "obes*") OR (TI "overweight" OR AB "overweight" OR SU "overweight") OR (TI "parent-child" OR AB "parent-child" OR SU "parent-child") OR (TI "parental" OR AB "parental" OR SU "parental") OR (TI "parenting" OR AB "parenting" OR SU "parenting") OR (TI "play" OR AB "play" OR SU "play")

OR (TI "prevent*" OR AB "prevent*" OR SU "prevent*") OR (TI "punish*" OR AB "punish*" OR SU "punish*") OR (TI "reading" OR AB "reading" OR SU "reading") OR (TI "safe" OR AB "safe" OR SU "safe") OR (TI "safety" OR AB "safety" OR SU "safety") OR (TI "security" OR AB "security" OR SU "security") OR (TI "stunt*" OR AB "stunt*" OR SU "stunt*") OR (TI "supervis*" OR AB "supervis*" OR SU "supervis*") OR (TI "supplement*" OR AB "supplement*" OR SU "supplement*") OR (TI "underweight" OR AB "underweight" OR SU "underweight") OR (TI "utiliz*" OR AB "utiliz*" OR SU "utiliz*") OR (TI "violence" OR AB "violence" OR SU "violence") OR (TI "psychosocial" OR AB "psychosocial" OR SU "psychosocial") OR (TI "infection*" OR AB "infection*" OR SU "infection*") OR (TI "condition*" OR AB "condition*" OR SU "condition*") OR (TI "vaccin*" OR AB "vaccin*" OR SU "vaccin*") OR (TI "disease*" OR AB "disease*" OR SU "disease*") OR (TI "disorder*" OR AB "disorder*" OR SU "disorder*") OR (TI "vitamin*" OR AB "vitamin*" OR SU "vitamin*") OR (TI "wasting" OR AB "wasting" OR SU "wasting") OR (TI "weight" OR AB "weight" OR SU "weight") OR (TI "well being" OR AB "well being" OR SU "well being") OR (TI "wellbeing" OR AB "wellbeing" OR SU "wellbeing")
**Line 5 - Combine concepts**
S1 AND S2 AND S3 AND S4
**Line 6 - Low and middle income countries**
(TI "afghan" OR AB "afghan" OR SU "afghan") OR (TI "afghanistan" OR AB "afghanistan" OR SU "afghanistan") OR (TI "albania" OR AB "albania" OR SU "albania") OR (TI "albanian" OR AB "albanian" OR SU "albanian") OR (TI "algeria" OR AB "algeria" OR SU "algeria") OR (TI "algerian" OR AB "algerian" OR SU "algerian") OR (TI "angola" OR AB "angola" OR SU "angola") OR (TI "angolan" OR AB "angolan" OR SU "angolan") OR (TI "argentina" OR AB "argentina" OR SU "argentina") OR (TI "argentinian" OR AB "argentinian" OR SU "argentinian") OR (TI "armenia" OR AB "armenia" OR SU "armenia") OR (TI "armenian" OR AB "armenian" OR SU "armenian") OR (TI "azerbaijan" OR AB "azerbaijan" OR SU "azerbaijan") OR (TI "azerbaijani" OR AB "azerbaijani" OR SU "azerbaijani") OR (TI "bajan" OR AB "bajan" OR SU "bajan") OR (TI "bangladesh" OR AB "bangladesh" OR SU "bangladesh") OR (TI "bangladeshi" OR AB "bangladeshi" OR SU "bangladeshi") OR (TI "belarus" OR AB "belarus" OR SU "belarus") OR (TI "belarusian" OR AB "belarusian" OR SU "belarusian") OR (TI "belize" OR AB "belize" OR SU "belize") OR (TI "belizean" OR AB "belizean" OR SU "belizean") OR (TI "benin" OR AB "benin" OR SU "benin") OR (TI "beninese" OR AB "beninese" OR SU "beninese") OR (TI "bhutan" OR AB "bhutan" OR SU "bhutan") OR (TI "bhutanese" OR AB "bhutanese" OR SU "bhutanese") OR (TI "bolivia" OR AB "bolivia" OR SU "bolivia") OR (TI "bolivian" OR AB "bolivian" OR SU "bolivian") OR (TI "bosnia" OR AB "bosnia" OR SU "bosnia") OR (TI "bosnian" OR AB "bosnian" OR SU "bosnian") OR (TI "botswana" OR AB "botswana" OR SU "botswana") OR (TI "brazil" OR AB "brazil" OR SU "brazil") OR (TI "brazilian" OR AB "brazilian" OR SU "brazilian") OR (TI

"burkina faso" OR AB "burkina faso" OR SU "burkina faso") OR (TI "burkinabes" OR AB "burkinabes" OR SU "burkinabes") OR (TI "burmese" OR AB "burmese" OR SU "burmese") OR (TI "burundi" OR AB "burundi" OR SU "burundi") OR (TI "burundian" OR AB "burundian" OR SU "burundian") OR (TI "cambodia" OR AB "cambodia" OR SU "cambodia") OR (TI "cambodian" OR AB "cambodian" OR SU "cambodian") OR (TI "cameroon" OR AB "cameroon" OR SU "cameroon") OR (TI "cameroonian" OR AB "cameroonian" OR SU "cameroonian") OR (TI "cape verde" OR AB "cape verde" OR SU "cape verde") OR (TI "cape verdeans" OR AB "cape verdeans" OR SU "cape verdeans") OR (TI "central african" OR AB "central african" OR SU "central african") OR (TI "central african republic" OR AB "central african republic" OR SU "central african republic") OR (TI "chad" OR AB "chad" OR SU "chad") OR (TI "chadian" OR AB "chadian" OR SU "chadian") OR (TI "china" OR AB "china" OR SU "china") OR (TI "chinese" OR AB "chinese" OR SU "chinese") OR (TI "colombia" OR AB "colombia" OR SU "colombia") OR (TI "colombian" OR AB "colombian" OR SU "colombian") OR (TI "comorian" OR AB "comorian" OR SU "comorian") OR (TI "comoros" OR AB "comoros" OR SU "comoros") OR (TI "congo" OR AB "congo" OR SU "congo") OR (TI "congolese" OR AB "congolese" OR SU "congolese") OR (TI "costa rica" OR AB "costa rica" OR SU "costa rica") OR (TI "costa rican" OR AB "costa rican" OR SU "costa rican") OR (TI "cote d'ivoire" OR AB "cote d'ivoire" OR SU "cote d'ivoire") OR (TI "cuba" OR AB "cuba" OR SU "cuba") OR (TI "cuban" OR AB "cuban" OR SU "cuban") OR (TI "djibouti" OR AB "djibouti" OR SU "djibouti") OR (TI "dominica" OR AB "dominica" OR SU "dominica") OR (TI "dominican" OR AB "dominican" OR SU "dominican") OR (TI "ecuador" OR AB "ecuador" OR SU "ecuador") OR (TI "ecuadorian" OR AB "ecuadorian" OR SU "ecuadorian") OR (TI "egypt" OR AB "egypt" OR SU "egypt") OR (TI "egyptian" OR AB "egyptian" OR SU "egyptian") OR (TI "el salvador" OR AB "el salvador" OR SU "el salvador") OR (TI "el salvadoran" OR AB "el salvadoran" OR SU "el salvadoran") OR (TI "eritrea" OR AB "eritrea" OR SU "eritrea") OR (TI "eritrean" OR AB "eritrean" OR SU "eritrean") OR (TI "eswatini" OR AB "eswatini" OR SU "eswatini") OR (TI "ethiopia" OR AB "ethiopia" OR SU "ethiopia") OR (TI "ethiopian" OR AB "ethiopian" OR SU "ethiopian") OR (TI "fiji" OR AB "fiji" OR SU "fiji") OR (TI "fijian" OR AB "fijian" OR SU "fijian") OR (TI "filipino" OR AB "filipino" OR SU "filipino") OR (TI "gabon" OR AB "gabon" OR SU "gabon") OR (TI "gabonese" OR AB "gabonese" OR SU "gabonese") OR (TI "gambia" OR AB "gambia" OR SU "gambia") OR (TI "gambian" OR AB "gambian" OR SU "gambian") OR (TI "gaza" OR AB "gaza" OR SU "gaza") OR (TI "ghana" OR AB "ghana" OR SU "ghana") OR (TI "ghanaian" OR AB "ghanaian" OR SU "ghanaian") OR (TI "grenada" OR AB "grenada" OR SU "grenada") OR (TI "grenadian" OR AB "grenadian" OR SU "grenadian") OR (TI "guatemala" OR AB "guatemala" OR SU "guatemala") OR (TI "guatemalan" OR AB "guatemalan" OR SU "guatemalan") OR (TI "guinea" OR AB "guinea" OR SU "guinea") OR (TI "guinean" OR AB "guinean" OR SU "guinean") OR (TI "haiti" OR AB "haiti" OR SU "haiti") OR (TI "haitian" OR AB "haitian" OR SU "haitian") OR (TI "honduran" OR AB "honduran" OR SU "honduran") OR (TI "honduras" OR AB "honduras" OR SU "honduras") OR (TI "india" OR AB "india" OR SU "india") OR (TI "indian" OR AB "indian" OR SU "indian") OR (TI "indonesia" OR AB "indonesia" OR SU "indonesia") OR (TI "indonesian" OR AB "indonesian" OR SU "indonesian") OR (TI "iran" OR AB "iran" OR SU "iran") OR (TI "iranian" OR AB "iranian" OR SU "iranian") OR (TI "iraq" OR AB "iraq" OR SU "iraq") OR (TI "iraqi" OR AB "iraqi" OR SU

"iraqi") OR (TI "jamaica" OR AB "jamaica" OR SU "jamaica") OR (TI "jamaica" OR AB "jamaica" OR SU "jamaica") OR (TI "jamaican" OR AB "jamaican" OR SU "jamaican") OR (TI "jamaican" OR AB "jamaican" OR SU "jamaican") OR (TI "jordan" OR AB "jordan" OR SU "jordan") OR (TI "jordan" OR AB "jordan" OR SU "jordan") OR (TI "jordanian" OR AB "jordanian" OR SU "jordanian") OR (TI "jordanian" OR AB "jordanian" OR SU "jordanian") OR (TI "kazakhstan" OR AB "kazakhstan" OR SU "kazakhstan") OR (TI "kazakhstan" OR AB "kazakhstan" OR SU "kazakhstan") OR (TI "kenya" OR AB "kenya" OR SU "kenya") OR (TI "kenya" OR AB "kenya" OR SU "kenya") OR (TI "kenyan" OR AB "kenyan" OR SU "kenyan") OR (TI "kenyan" OR AB "kenyan" OR SU "kenyan") OR (TI "kiribati" OR AB "kiribati" OR SU "kiribati") OR (TI "kiribati" OR AB "kiribati" OR SU "kiribati") OR (TI "kosovar" OR AB "kosovar" OR SU "kosovar") OR (TI "kosovar" OR AB "kosovar" OR SU "kosovar") OR (TI "kosovo" OR AB "kosovo" OR SU "kosovo") OR (TI "kosovo" OR AB "kosovo" OR SU "kosovo") OR (TI "kyrgyz" OR AB "kyrgyz" OR SU "kyrgyz") OR (TI "kyrgyz" OR AB "kyrgyz" OR SU "kyrgyz") OR (TI "kyrgyzstan" OR AB "kyrgyzstan" OR SU "kyrgyzstan") OR (TI "kyrgyzstan" OR AB "kyrgyzstan" OR SU "kyrgyzstan") OR (TI "Lao" OR AB "Lao" OR SU "Lao") OR (TI "Lao" OR AB "Lao" OR SU "Lao") OR (TI "laos" OR AB "laos" OR SU "laos") OR (TI "laos" OR AB "laos" OR SU "laos") OR (TI "laotian" OR AB "laotian" OR SU "laotian") OR (TI "laotian" OR AB "laotian" OR SU "laotian") OR (TI "lebanese" OR AB "lebanese" OR SU "lebanese") OR (TI "lebanese" OR AB "lebanese" OR SU "lebanese") OR (TI "lebanon" OR AB "lebanon" OR SU "lebanon") OR (TI "lebanon" OR AB "lebanon" OR SU "lebanon") OR (TI "lesotho" OR AB "lesotho" OR SU "lesotho") OR (TI "lesotho" OR AB "lesotho" OR SU "lesotho") OR (TI "liberia" OR AB "liberia" OR SU "liberia") OR (TI "liberia" OR AB "liberia" OR SU "liberia") OR (TI "liberian" OR AB "liberian" OR SU "liberian") OR (TI "liberian" OR AB "liberian" OR SU "liberian") OR (TI "libya" OR AB "libya" OR SU "libya") OR (TI "libya" OR AB "libya" OR SU "libya") OR (TI "libyan" OR AB "libyan" OR SU "libyan") OR (TI "libyan" OR AB "libyan" OR SU "libyan") OR (TI "macedonia" OR AB "macedonia" OR SU "macedonia") OR (TI "macedonia" OR AB "macedonia" OR SU "macedonia") OR (TI "macedonian" OR AB "macedonian" OR SU "macedonian") OR (TI "macedonian" OR AB "macedonian" OR SU "macedonian") OR (TI "madagascar" OR AB "madagascar" OR SU "madagascar") OR (TI "madagascar" OR AB "madagascar" OR SU "madagascar") OR (TI "malagasy" OR AB "malagasy" OR SU "malagasy") OR (TI "malagasy" OR AB "malagasy" OR SU "malagasy") OR (TI "malawi" OR AB "malawi" OR SU "malawi") OR (TI "malawi" OR AB "malawi" OR SU "malawi") OR (TI "malawian" OR AB "malawian" OR SU "malawian") OR (TI "malawian" OR AB "malawian" OR SU "malawian") OR (TI "malaysia" OR AB "malaysia" OR SU "malaysia") OR (TI "malaysia" OR AB "malaysia" OR SU "malaysia") OR (TI "malaysian" OR AB "malaysian" OR SU "malaysian") OR (TI "malaysian" OR AB "malaysian" OR SU "malaysian") OR (TI "maldives" OR AB "maldives" OR SU "maldives") OR (TI "maldives" OR AB "maldives" OR SU "maldives") OR (TI "maldivian" OR AB "maldivian" OR SU "maldivian") OR (TI "maldivian" OR AB "maldivian" OR SU "maldivian") OR (TI "mali" OR AB "mali" OR SU "mali") OR (TI "mali" OR AB "mali" OR SU "mali") OR (TI "malian" OR AB "malian" OR SU "malian") OR (TI "malian" OR AB "malian" OR SU "malian") OR (TI "marshall islands" OR AB "marshall islands" OR SU "marshall islands") OR (TI "marshall islands" OR AB "marshall islands" OR SU "marshall islands") OR (TI "marshallese" OR AB "marshallese" OR SU "marshallese") OR (TI

"marshallese" OR AB "marshallese" OR SU "marshallese") OR (TI "mauritania" OR AB "mauritania" OR SU "mauritania") OR (TI "mauritania" OR AB "mauritania" OR SU "mauritania") OR (TI "mauritanian" OR AB "mauritanian" OR SU "mauritanian") OR (TI "mauritanian" OR AB "mauritanian" OR SU "mauritanian") OR (TI "mauritian" OR AB "mauritian" OR SU "mauritian") OR (TI "mauritian" OR AB "mauritian" OR SU "mauritian") OR (TI "mauritius" OR AB "mauritius" OR SU "mauritius") OR (TI "mauritius" OR AB "mauritius" OR SU "mauritius") OR (TI "mexican" OR AB "mexican" OR SU "mexican") OR (TI "mexican" OR AB "mexican" OR SU "mexican") OR (TI "mexico" OR AB "mexico" OR SU "mexico") OR (TI "mexico" OR AB "mexico" OR SU "mexico") OR (TI "micronesia" OR AB "micronesia" OR SU "micronesia") OR (TI "micronesia" OR AB "micronesia" OR SU "micronesia") OR (TI "micronesian" OR AB "micronesian" OR SU "micronesian") OR (TI "micronesian" OR AB "micronesian" OR SU "micronesian") OR (TI "moldova" OR AB "moldova" OR SU "moldova") OR (TI "moldova" OR AB "moldova" OR SU "moldova") OR (TI "moldovan" OR AB "moldovan" OR SU "moldovan") OR (TI "moldovan" OR AB "moldovan" OR SU "moldovan") OR (TI "mongolia" OR AB "mongolia" OR SU "mongolia") OR (TI "mongolia" OR AB "mongolia" OR SU "mongolia") OR (TI "mongolian" OR AB "mongolian" OR SU "mongolian") OR (TI "mongolian" OR AB "mongolian" OR SU "mongolian") OR (TI "montenegrin" OR AB "montenegrin" OR SU "montenegrin") OR (TI "montenegrin" OR AB "montenegrin" OR SU "montenegrin") OR (TI "montenegro" OR AB "montenegro" OR SU "montenegro") OR (TI "montenegro" OR AB "montenegro" OR SU "montenegro") OR (TI "moroccan" OR AB "moroccan" OR SU "moroccan") OR (TI "moroccan" OR AB "moroccan" OR SU "moroccan") OR (TI "morocco" OR AB "morocco" OR SU "morocco") OR (TI "morocco" OR AB "morocco" OR SU "morocco") OR (TI "mozambican" OR AB "mozambican" OR SU "mozambican") OR (TI "mozambican" OR AB "mozambican" OR SU "mozambican") OR (TI "mozambique" OR AB "mozambique" OR SU "mozambique") OR (TI "mozambique" OR AB "mozambique" OR SU "mozambique") OR (TI "myanmar" OR AB "myanmar" OR SU "myanmar") OR (TI "myanmar" OR AB "myanmar" OR SU "myanmar") OR (TI "namibia" OR AB "namibia" OR SU "namibia") OR (TI "namibian" OR AB "namibian" OR SU "namibian") OR (TI "nepal" OR AB "nepal" OR SU "nepal") OR (TI "nepalese" OR AB "nepalese" OR SU "nepalese") OR (TI "nicaragua" OR AB "nicaragua" OR SU "nicaragua") OR (TI "nicaraguan" OR AB "nicaraguan" OR SU "nicaraguan") OR (TI "niger" OR AB "niger" OR SU "niger") OR (TI "nigeria" OR AB "nigeria" OR SU "nigeria") OR (TI "nigerian" OR AB "nigerian" OR SU "nigerian") OR (TI "north korea" OR AB "north korea" OR SU "north korea") OR (TI "north korea" OR AB "north korea" OR SU "north korea") OR (TI "north korean" OR AB "north korean" OR SU "north korean") OR (TI "north korean" OR AB "north korean" OR SU "north korean") OR (TI "pakistan" OR AB "pakistan" OR SU "pakistan") OR (TI "pakistani" OR AB "pakistani" OR SU "pakistani") OR (TI "palau" OR AB "palau" OR SU "palau") OR (TI "palauan" OR AB "palauan" OR SU "palauan") OR (TI "panama" OR AB "panama" OR SU "panama") OR (TI "panamanian" OR AB "panamanian" OR SU "panamanian") OR (TI "papua new guinea" OR AB "papua new guinea" OR SU "papua new guinea") OR (TI "papua new guinean" OR AB "papua new guinean" OR SU "papua new guinean") OR (TI "paraguay" OR AB "paraguay" OR SU "paraguay") OR (TI "paraguayan" OR AB "paraguayan" OR SU "paraguayan") OR (TI "peru" OR AB "peru" OR SU "peru") OR (TI "peruvian" OR AB "peruvian" OR SU "peruvian") OR (TI "philippines" OR

AB "philippines" OR SU "philippines") OR (TI "principe" OR AB "principe" OR SU "principe") OR (TI "romania" OR AB "romania" OR SU "romania") OR (TI "romanian" OR AB "romanian" OR SU "romanian") OR (TI "russia" OR AB "russia" OR SU "russia") OR (TI "russian" OR AB "russian" OR SU "russian") OR (TI "rwanda" OR AB "rwanda" OR SU "rwanda") OR (TI "rwandan" OR AB "rwandan" OR SU "rwandan") OR (TI "saint kitts" OR AB "saint kitts" OR SU "saint kitts") OR (TI "saint lucia" OR AB "saint lucia" OR SU "saint lucia") OR (TI "saint vincent" OR AB "saint vincent" OR SU "saint vincent") OR (TI "salvadoran" OR AB "salvadoran" OR SU "salvadoran") OR (TI "samoa" OR AB "samoa" OR SU "samoa") OR (TI "samoan" OR AB "samoan" OR SU "samoan") OR (TI "santomea" OR AB "santomea" OR SU "santomea") OR (TI "sao tome" OR AB "sao tome" OR SU "sao tome") OR (TI "senegal" OR AB "senegal" OR SU "senegal") OR (TI "senegalese" OR AB "senegalese" OR SU "senegalese") OR (TI "serbia" OR AB "serbia" OR SU "serbia") OR (TI "serbian" OR AB "serbian" OR SU "serbian") OR (TI "seychelles" OR AB "seychelles" OR SU "seychelles") OR (TI "seychellois" OR AB "seychellois" OR SU "seychellois") OR (TI "sierra leone" OR AB "sierra leone" OR SU "sierra leone") OR (TI "sierra leoneans" OR AB "sierra leoneans" OR SU "sierra leoneans") OR (TI "solomon islander" OR AB "solomon islander" OR SU "solomon islander") OR (TI "solomon islands" OR AB "solomon islands" OR SU "solomon islands") OR (TI "somalia" OR AB "somalia" OR SU "somalia") OR (TI "somalian" OR AB "somalian" OR SU "somalian") OR (TI "south africa" OR AB "south africa" OR SU "south africa") OR (TI "south african" OR AB "south african" OR SU "south african") OR (TI "sri lanka" OR AB "sri lanka" OR SU "sri lanka") OR (TI "sri lankan" OR AB "sri lankan" OR SU "sri lankan") OR (TI "sudan" OR AB "sudan" OR SU "sudan") OR (TI "sudanese" OR AB "sudanese" OR SU "sudanese") OR (TI "suriname" OR AB "suriname" OR SU "suriname") OR (TI "swazi" OR AB "swazi" OR SU "swazi") OR (TI "swaziland" OR AB "swaziland" OR SU "swaziland") OR (TI "syria" OR AB "syria" OR SU "syria") OR (TI "syrian" OR AB "syrian" OR SU "syrian") OR (TI "tadzhik" OR AB "tadzhik" OR SU "tadzhik") OR (TI "tajik" OR AB "tajik" OR SU "tajik") OR (TI "tajikistan" OR AB "tajikistan" OR SU "tajikistan") OR (TI "tanzania" OR AB "tanzania" OR SU "tanzania") OR (TI "tanzanian" OR AB "tanzanian" OR SU "tanzanian") OR (TI "thai" OR AB "thai" OR SU "thai") OR (TI "thailand" OR AB "thailand" OR SU "thailand") OR (TI "timor" OR AB "timor" OR SU "timor") OR (TI "togo" OR AB "togo" OR SU "togo") OR (TI "togolese" OR AB "togolese" OR SU "togolese") OR (TI "tonga" OR AB "tonga" OR SU "tonga") OR (TI "tongan" OR AB "tongan" OR SU "tongan") OR (TI "tunisia" OR AB "tunisia" OR SU "tunisia") OR (TI "tunisian" OR AB "tunisian" OR SU "tunisian") OR (TI "turkey" OR AB "turkey" OR SU "turkey") OR (TI "turkish" OR AB "turkish" OR SU "turkish") OR (TI "turkmen" OR AB "turkmen" OR SU "turkmen") OR (TI "turkmenistan" OR AB "turkmenistan" OR SU "turkmenistan") OR (TI "tuvalu" OR AB "tuvalu" OR SU "tuvalu") OR (TI "tuvaluans" OR AB "tuvaluans" OR SU "tuvaluans") OR (TI "uganda" OR AB "uganda" OR SU "uganda") OR (TI "ugandan" OR AB "ugandan" OR SU "ugandan") OR (TI "ukraine" OR AB "ukraine" OR SU "ukraine") OR (TI "ukrainian" OR AB "ukrainian" OR SU "ukrainian") OR (TI "uzbek" OR AB "uzbek" OR SU "uzbek") OR (TI "uzbekistan" OR AB "uzbekistan" OR SU "uzbekistan") OR (TI "vanuatu" OR AB "vanuatu" OR SU "vanuatu") OR (TI "venezuela" OR AB "venezuela" OR SU "venezuela") OR (TI "venezuelan" OR AB "venezuelan" OR SU "venezuelan") OR (TI "vietnam" OR AB "vietnam" OR SU "vietnam") OR (TI "vietnamese" OR AB "vietnamese" OR

SU "vietnamese") OR (TI "west bank" OR AB "west bank" OR SU "west bank") OR (TI "yemen" OR AB "yemen" OR SU "yemen") OR (TI "yemeni" OR AB "yemeni" OR SU "yemeni") OR (TI "yemenite" OR AB "yemenite" OR SU "yemenite") OR (TI "zambia" OR AB "zambia" OR SU "zambia") OR (TI "zambian" OR AB "zambian" OR SU "zambian") OR (TI "zimbabwe" OR AB "zimbabwe" OR SU "zimbabwe") OR (TI "zimbabwean" OR AB "zimbabwean" OR SU "zimbabwean")
**Line 7 - High income countries**
(TI "Alabama" OR AB "Alabama" OR MW "Alabama") OR (TI "Alaska" OR AB "Alaska" OR MW "Alaska") OR (TI "American Samoa" OR AB "American Samoa" OR MW "American Samoa") OR (TI "Andorra" OR AB "Andorra" OR MW "Andorra") OR (TI ""Antigua and Barbuda"" OR AB ""Antigua and Barbuda"" OR MW ""Antigua and Barbuda"") OR (TI "Arizona" OR AB "Arizona" OR MW "Arizona") OR (TI "Arkansas" OR AB "Arkansas" OR MW "Arkansas") OR (TI "Aruba" OR AB "Aruba" OR MW "Aruba") OR (TI "Australia" OR AB "Australia" OR MW "Australia") OR (TI "Austria" OR AB "Austria" OR MW "Austria") OR (TI "Bahamas" OR AB "Bahamas" OR MW "Bahamas") OR (TI "Bahrain" OR AB "Bahrain" OR MW "Bahrain") OR (TI "Barbados" OR AB "Barbados" OR MW "Barbados") OR (TI "Belgium" OR AB "Belgium" OR MW "Belgium") OR (TI "Bermuda" OR AB "Bermuda" OR MW "Bermuda") OR (TI "Brunei" OR AB "Brunei" OR MW "Brunei") OR (TI "Bulgaria" OR AB "Bulgaria" OR MW "Bulgaria") OR (TI "California" OR AB "California" OR MW "California") OR (TI "Canada" OR AB "Canada" OR MW "Canada") OR (TI "Cayman Islands" OR AB "Cayman Islands" OR MW "Cayman Islands") OR (TI "Channel Islands" OR AB "Channel Islands" OR MW "Channel Islands") OR (TI "Chile" OR AB "Chile" OR MW "Chile") OR (TI "Colorado" OR AB "Colorado" OR MW "Colorado") OR (TI "Connecticut" OR AB "Connecticut" OR MW "Connecticut") OR (TI "Croatia" OR AB "Croatia" OR MW "Croatia") OR (TI "Curacao" OR AB "Curacao" OR MW "Curacao") OR (TI "Cyprus" OR AB "Cyprus" OR MW "Cyprus") OR (TI "Czech*" OR AB "Czech*" OR MW "Czech*") OR (TI "Czechia" OR AB "Czechia" OR MW "Czechia") OR (TI "Delaware" OR AB "Delaware" OR MW "Delaware") OR (TI "Denmark" OR AB "Denmark" OR MW "Denmark") OR (TI "District of Columbia" OR AB "District of Columbia" OR MW "District of Columbia") OR (TI "england" OR AB "england" OR MW "england") OR (TI "Estonia" OR AB "Estonia" OR MW "Estonia") OR (TI "Faroe Islands" OR AB "Faroe Islands" OR MW "Faroe Islands") OR (TI "Finland" OR AB "Finland" OR MW "Finland") OR (TI "Florida" OR AB "Florida" OR MW "Florida") OR (TI "France" OR AB "France" OR MW "France") OR (TI "French Polynesia" OR AB "French Polynesia" OR MW "French Polynesia") OR (TI "Georgia" OR AB "Georgia" OR MW "Georgia") OR (TI "Germany" OR AB "Germany" OR MW "Germany") OR (TI "Gibraltar" OR AB "Gibraltar" OR MW "Gibraltar") OR (TI "Greece" OR AB "Greece" OR MW "Greece") OR (TI "Greenland" OR AB "Greenland" OR MW "Greenland") OR (TI "Guam" OR AB "Guam" OR MW "Guam") OR (TI "Guyana" OR AB "Guyana" OR MW "Guyana") OR (TI "Hawaii" OR AB "Hawaii" OR MW "Hawaii") OR (TI "Hong Kong" OR AB "Hong Kong" OR MW "Hong Kong") OR (TI "Hungary" OR AB "Hungary" OR MW "Hungary") OR (TI "Iceland*" OR AB "Iceland*" OR MW "Iceland*") OR (TI "Idaho*"

OR AB "Idaho*" OR MW "Idaho*") OR (TI "Illinois*" OR AB "Illinois*" OR MW "Illinois*") OR (TI "Indiana*" OR AB "Indiana*" OR MW "Indiana*") OR (TI "Iowa*" OR AB "Iowa*" OR MW "Iowa*") OR (TI "Ireland" OR AB "Ireland" OR MW "Ireland") OR (TI "Isle of Man" OR AB "Isle of Man" OR MW "Isle of Man") OR (TI "Israel" OR AB "Israel" OR MW "Israel") OR (TI "Italy" OR AB "Italy" OR MW "Italy") OR (TI "Japan" OR AB "Japan" OR MW "Japan") OR (TI "Kansas" OR AB "Kansas" OR MW "Kansas") OR (TI "Kentucky" OR AB "Kentucky" OR MW "Kentucky") OR (TI "Korea" OR AB "Korea" OR MW "Korea") OR (TI "Kuwait" OR AB "Kuwait" OR MW "Kuwait") OR (TI "Latvia" OR AB "Latvia" OR MW "Latvia") OR (TI "Liechtenstein" OR AB "Liechtenstein" OR MW "Liechtenstein") OR (TI "Lithuania" OR AB "Lithuania" OR MW "Lithuania") OR (TI "Louisiana*" OR AB "Louisiana*" OR MW "Louisiana*") OR (TI "Luxembourg" OR AB "Luxembourg" OR MW "Luxembourg") OR (TI "Macao" OR AB "Macao" OR MW "Macao") OR (TI "Maine" OR AB "Maine" OR MW "Maine") OR (TI "Malta" OR AB "Malta" OR MW "Malta") OR (TI "Maryland*" OR AB "Maryland*" OR MW "Maryland*") OR (TI "Massachusetts" OR AB "Massachusetts" OR MW "Massachusetts") OR (TI "Michigan" OR AB "Michigan" OR MW "Michigan") OR (TI "Minnesota" OR AB "Minnesota" OR MW "Minnesota") OR (TI "Mississippi" OR AB "Mississippi" OR MW "Mississippi") OR (TI "Missouri" OR AB "Missouri" OR MW "Missouri") OR (TI "Monaco" OR AB "Monaco" OR MW "Monaco") OR (TI "Montana" OR AB "Montana" OR MW "Montana") OR (TI "Nauru" OR AB "Nauru" OR MW "Nauru") OR (TI "Nebraska" OR AB "Nebraska" OR MW "Nebraska") OR (TI "Netherlands" OR AB "Netherlands" OR MW "Netherlands") OR (TI "Nevada" OR AB "Nevada" OR MW "Nevada") OR (TI "New Caledonia" OR AB "New Caledonia" OR MW "New Caledonia") OR (TI "New Hampshire" OR AB "New Hampshire" OR MW "New Hampshire") OR (TI "New Jersey" OR AB "New Jersey" OR MW "New Jersey") OR (TI "New Mexico" OR AB "New Mexico" OR MW "New Mexico") OR (TI "New York" OR AB "New York" OR MW "New York") OR (TI "New Zealand" OR AB "New Zealand" OR MW "New Zealand") OR (TI "North Carolina" OR AB "North Carolina" OR MW "North Carolina") OR (TI "North Dakota" OR AB "North Dakota" OR MW "North Dakota") OR (TI "Northern Mariana Islands" OR AB "Northern Mariana Islands" OR MW "Northern Mariana Islands") OR (TI "Norway" OR AB "Norway" OR MW "Norway") OR (TI "Ohio" OR AB "Ohio" OR MW "Ohio") OR (TI "Oklahoma" OR AB "Oklahoma" OR MW "Oklahoma") OR (TI "Oman" OR AB "Oman" OR MW "Oman") OR (TI "Oregon" OR AB "Oregon" OR MW "Oregon") OR (TI "Palau" OR AB "Palau" OR MW "Palau") OR (TI "Panama" OR AB "Panama" OR MW "Panama") OR (TI "Pennsylvania" OR AB "Pennsylvania" OR MW "Pennsylvania") OR (TI "Poland" OR AB "Poland" OR MW "Poland") OR (TI "Portugal" OR AB "Portugal" OR MW "Portugal") OR (TI "Puerto Rico" OR AB "Puerto Rico" OR MW "Puerto Rico") OR (TI "Qatar" OR AB "Qatar" OR MW "Qatar") OR (TI "Rhode Island" OR AB "Rhode Island" OR MW "Rhode Island") OR (TI "Romania" OR AB "Romania" OR MW "Romania") OR (TI "Russia" OR AB "Russia" OR MW "Russia") OR (TI "Russian" OR AB "Russian" OR MW "Russian") OR (TI ""Saint Kitts and Nevis"" OR AB ""Saint Kitts and Nevis"" OR MW ""Saint Kitts and Nevis"") OR (TI "Saint Martin" OR AB "Saint Martin" OR MW "Saint Martin") OR (TI "San Marino" OR AB "San Marino" OR MW "San Marino") OR (TI "Saudi Arabia" OR AB "Saudi Arabia" OR MW "Saudi Arabia") OR (TI "scotland" OR AB "scotland" OR MW "scotland") OR (TI "Seychelles" OR AB "Seychelles" OR MW "Seychelles") OR (TI "Singapore" OR AB "Singapore" OR MW "Singapore") OR (TI "Sint

Maarten" OR AB "Sint Maarten" OR MW "Sint Maarten") OR (TI "Slovak Republic" OR AB "Slovak Republic" OR MW "Slovak Republic") OR (TI "Slovakia" OR AB "Slovakia" OR MW "Slovakia") OR (TI "Slovenia" OR AB "Slovenia" OR MW "Slovenia") OR (TI "South Carolina" OR AB "South Carolina" OR MW "South Carolina") OR (TI "South Dakota" OR AB "South Dakota" OR MW "South Dakota") OR (TI "Spain" OR AB "Spain" OR MW "Spain") OR (TI "St Martin" OR AB "St Martin" OR MW "St Martin") OR (TI "Sweden" OR AB "Sweden" OR MW "Sweden") OR (TI "Switzerland" OR AB "Switzerland" OR MW "Switzerland") OR (TI "Taiwan" OR AB "Taiwan" OR MW "Taiwan") OR (TI "Tennessee" OR AB "Tennessee" OR MW "Tennessee") OR (TI "Texas" OR AB "Texas" OR MW "Texas") OR (TI "Tobago" OR AB "Tobago" OR MW "Tobago") OR (TI "Trinidad" OR AB "Trinidad" OR MW "Trinidad") OR (TI ""Turks and Caicos"" OR AB ""Turks and Caicos"" OR MW ""Turks and Caicos"") OR (TI "United Arab Emirates" OR AB "United Arab Emirates" OR MW "United Arab Emirates") OR (TI "United Kingdom" OR AB "United Kingdom" OR MW "United Kingdom") OR (TI "United States" OR AB "United States" OR MW "United States") OR (TI "Uruguay" OR AB "Uruguay" OR MW "Uruguay") OR (TI "Utah" OR AB "Utah" OR MW "Utah") OR (TI "Vermont" OR AB "Vermont" OR MW "Vermont") OR (TI "Virgin Islands" OR AB "Virgin Islands" OR MW "Virgin Islands") OR (TI "Virginia" OR AB "Virginia" OR MW "Virginia") OR (TI "wales" OR AB "wales" OR MW "wales") OR (TI "Washington" OR AB "Washington" OR MW "Washington") OR (TI "West Virginia" OR AB "West Virginia" OR MW "West Virginia") OR (TI "Wisconsin" OR AB "Wisconsin" OR MW "Wisconsin") OR (TI "Wyoming" OR AB "Wyoming" OR MW "Wyoming") OR (TI "Alberta" OR AB "Alberta" OR MW "Alberta") OR (TI "British Columbia" OR AB "British Columbia" OR MW "British Columbia") OR (TI "Manitoba" OR AB "Manitoba" OR MW "Manitoba") OR (TI "New Brunswick" OR AB "New Brunswick" OR MW "New Brunswick") OR (TI "Newfoundland" OR AB "Newfoundland" OR MW "Newfoundland") OR (TI "Northwest Territories" OR AB "Northwest Territories" OR MW "Northwest Territories") OR (TI "Nova Scotia" OR AB "Nova Scotia" OR MW "Nova Scotia") OR (TI "Nunavut" OR AB "Nunavut" OR MW "Nunavut") OR (TI "Ontario" OR AB "Ontario" OR MW "Ontario") OR (TI "Prince Edward Island" OR AB "Prince Edward Island" OR MW "Prince Edward Island") OR (TI "Quebec" OR AB "Quebec" OR MW "Quebec") OR (TI "Saskatchewan" OR AB "Saskatchewan" OR MW "Saskatchewan") OR (TI "Yukon Territory" OR AB "Yukon Territory" OR MW "Yukon Territory") OR (SU "Canada+") OR (SU "United States+") OR (SU "United Kingdom+") OR (SU "Australia+")
**Line 8 - filter out LMICs unless they also mention HICs**
S5 not (S6 not S7)
Used platform options to filter to:<br>Academic Journals<br>2000-2024

English

SocIndex
**Line 1 - Homelessness**
((TI "displace*" OR AB "displace*") OR (TI "encampment*" OR AB "encampment*") OR (TI "evict*" OR AB "evict*") OR (TI "farm worker*" OR AB "farm worker*") OR (TI "farmworker*" OR AB "farmworker*") OR (TI "food pantr*" OR AB "food pantr*") OR (TI "homeless*" OR AB "homeless*") OR (TI "houseless*" OR AB "houseless*") OR (TI "housing condition*" OR AB "housing condition*") OR (TI "housing first" OR AB "housing first") OR (TI "housing insecur*" OR AB "housing insecur*") OR (TI "housing instability" OR AB "housing instability") OR (TI "housing problem*" OR AB "housing problem*") OR (TI "housing quality" OR AB "housing quality") OR (TI "housing secur*" OR AB "housing secur*") OR (TI "housing stabil*" OR AB "housing stabil*") OR (TI "ill housed" OR AB "ill housed") OR (TI "insecure accommodation*" OR AB "insecure accommodation*") OR (TI "insecure housing" OR AB "insecure housing") OR (TI "insecurely housed" OR AB "insecurely housed") OR (TI "insufficient housing" OR AB "insufficient housing") OR (TI "migrant*" OR AB "migrant*") OR (TI "refugee*" OR AB "refugee*") OR (TI "resettlement*" OR AB "resettlement*") OR (TI "residential mobility" OR AB "residential mobility") OR (TI "roofless*" OR AB "roofless*") OR (TI "rough sleep*" OR AB "rough sleep*") OR (TI "runaway*" OR AB "runaway*") OR (TI "running away*" OR AB "running away*") OR (TI "settlement*" OR AB "settlement*") OR (TI "shelter*" OR AB "shelter*") OR (TI "short term accommodation*" OR AB "short term accommodation*") OR (TI "short term housing" OR AB "short term housing") OR (TI "slum*" OR AB "slum*") OR (TI "sofa surf*" OR AB "sofa surf*") OR (TI "soup kitchen*" OR AB "soup kitchen*") OR (TI "squatter*" OR AB "squatter*") OR (TI "squatting" OR AB "squatting") OR (TI "street child*" OR AB "street child*") OR (TI "street connected" OR AB "street connected") OR (TI "street dwell*" OR AB "street dwell*") OR (TI "street involved" OR AB "street involved") OR (TI "street people" OR AB "street people") OR (TI "street youth" OR AB "street youth") OR (TI "temporary accommodation*" OR AB "temporary accommodation*") OR (TI "temporary camp*" OR AB "temporary camp*") OR (TI "temporary housing" OR AB "temporary housing") OR (TI "unhoused" OR AB "unhoused") OR (TI "unsheltered" OR AB "unsheltered") OR (TI "unstable housing" OR AB "unstable housing") OR (TI "unstably housed" OR AB "unstably housed") OR SU "Homeless People" OR SU "Emergency Shelters" OR SU "Housing Needs" OR SU "Homeless students" OR SU "Education of homeless children" OR SU "Homeless children")
**Line 2 - Co-development**

((TI "citizen scien*" OR AB "citizen scien*") OR (TI "co-construct*" OR AB "co-construct*") OR (TI "co-creat*" OR AB "co-creat*") OR (TI "co-design*" OR AB "co-design*") OR (TI "co-develop*" OR AB "co-develop*") OR (TI "co-produc*" OR AB "co-produc*") OR (TI "co-research*" OR AB "co-research*") OR (TI "coconstruct*" OR AB "coconstruct*") OR (TI "cocreat*" OR AB "cocreat*") OR (TI "codesign*" OR AB "codesign*") OR (TI "codevelop*" OR AB "codevelop*") OR (TI "community driven" OR AB "community driven") OR (TI "community engage*" OR AB "community engage*") OR (TI "community informed" OR AB "community informed") OR (TI "community involve*" OR AB "community involve*") OR (TI "community led" OR AB "community led") OR (TI "community scien*" OR AB "community scien*") OR (TI "coproduc*" OR AB "coproduc*") OR (TI "coresearch*" OR AB "coresearch*") OR (TI "inclusive research" OR AB "inclusive research") OR (TI "participative" OR AB "participative") OR (TI "participatory" OR AB "participatory") OR (TI "partnership research" OR AB "partnership research") OR (TI "peer research*" OR AB "peer research*") OR (TI "public engage*" OR AB "public engage*") OR (TI "public involve*" OR AB "public involve*") OR (TI "public participation" OR AB "public participation") OR (TI "stakeholder driven" OR AB "stakeholder driven") OR (TI "stakeholder engage*" OR AB "stakeholder engage*") OR (TI "stakeholder involve*" OR AB "stakeholder involve*") OR (TI "stakeholder led" OR AB "stakeholder led") OR (TI "youth driven" OR AB "youth driven") OR (TI "youth led" OR AB "youth led") OR SU "Action Research" OR SU "Participatory Research" OR SU "Action research" OR SU "Action research in education")
**Line 3 - Children/familie**s
((TI "adolescen*" OR AB "adolescen*") OR (TI "babies" OR AB "babies") OR (TI "baby" OR AB "baby") OR (TI "boy" OR AB "boy") OR (TI "boys" OR AB "boys") OR (TI "caregiver*" OR AB "caregiver*") OR (TI "child" OR AB "child") OR (TI "childhood" OR AB "childhood") OR (TI "children" OR AB "children") OR (TI "families" OR AB "families") OR (TI "family" OR AB "family") OR (TI "father" OR AB "father") OR (TI "fathers" OR AB "fathers") OR (TI "girl*" OR AB "girl*") OR (TI "infant*" OR AB "infant*") OR (TI "minors" OR AB "minors") OR (TI "mother" OR AB "mother") OR (TI "mothers" OR AB "mothers") OR (TI "neonat*" OR AB "neonat*") OR (TI "newborn*" OR AB "newborn*") OR (TI "parent" OR AB "parent") OR (TI "parents" OR AB "parents") OR (TI "pediatri*" OR AB "pediatri*") OR (TI "teen*" OR AB "teen*") OR (TI "toddler*" OR AB "toddler*") OR (TI "youth" OR AB "youth") OR SU "Children" OR SU "Youth" OR SU "Families" OR SU "Children" OR SU "Dependents" OR SU "Dysfunctional families" OR SU "Families of people with disabilities" OR SU "Family roles" OR SU "Family size" OR SU "Family structure" OR SU "Family support" OR SU "Family-school relationships" OR SU "Fatherless families" OR SU "Fathers" OR SU "Heads of households" OR SU "Host families of foreign students" OR SU "Language in families" OR SU "Motherless families" OR SU "Mothers" OR SU "Parent-child relationships" OR SU "Parents" OR SU "Siblings" OR SU "Single-parent families" OR SU "Stepfamilies" OR SU "Students' families")

Line 4 - Health outcomes
(TI "abus*" OR AB "abus*" OR SU "abus*") OR (TI "behavioral" OR AB "behavioral" OR SU "behavioral") OR (TI "behavioural" OR AB "behavioural" OR SU "behavioural") OR (TI "birth*" OR AB "birth*" OR SU "birth*") OR (TI "bodyweight" OR AB "bodyweight" OR SU "bodyweight") OR (TI "books" OR AB "books" OR SU "books") OR (TI "breast fe*" OR AB "breast fe*" OR SU "breast fe*") OR (TI "breast milk" OR AB "breast milk" OR SU "breast milk") OR (TI "breastfe*" OR AB "breastfe*" OR SU "breastfe*") OR (TI "breastmilk" OR AB "breastmilk" OR SU "breastmilk") OR (TI "caregiver-child" OR AB "caregiver-child" OR SU "caregiver-child") OR (TI "caregiving" OR AB "caregiving" OR SU "caregiving") OR (TI "child care" OR AB "child care" OR SU "child care") OR (TI "child development*" OR AB "child development*" OR SU "child development*") OR (TI "childbirth*" OR AB "childbirth*" OR SU "childbirth*") OR (TI "childcare" OR AB "childcare" OR SU "childcare") OR (TI "childhood" OR AB "childhood" OR SU "childhood") OR (TI "childhood development" OR AB "childhood development" OR SU "childhood development") OR (TI "children's development*" OR AB "children's development*" OR SU "children's development*") OR (TI "circumference" OR AB "circumference" OR SU "circumference") OR (TI "clean*" OR AB "clean*" OR SU "clean*") OR (TI "diet*" OR AB "diet*" OR SU "diet*") OR (TI "disciplin*" OR AB "disciplin*" OR SU "disciplin*") OR (TI "education" OR AB "education" OR SU "education") OR (TI "familial" OR AB "familial" OR SU "familial") OR (TI "father child" OR AB "father child" OR SU "father child") OR (TI "feeding" OR AB "feeding" OR SU "feeding") OR (TI "food*" OR AB "food*" OR SU "food*") OR (TI "gestation*" OR AB "gestation*" OR SU "gestation*") OR (TI "health*" OR AB "health*" OR SU "health*") OR (TI "height" OR AB "height" OR SU "height") OR (TI "hunger" OR AB "hunger" OR SU "hunger") OR (TI "hygien*" OR AB "hygien*" OR SU "hygien*") OR (TI "infant development*" OR AB "infant development*" OR SU "infant development*") OR (TI "lactat*" OR AB "lactat*" OR SU "lactat*") OR (TI "learning" OR AB "learning" OR SU "learning") OR (TI "literacy" OR AB "literacy" OR SU "literacy") OR (TI "malnutrition" OR AB "malnutrition" OR SU "malnutrition") OR (TI "maternal child" OR AB "maternal child" OR SU "maternal child") OR (TI "maternal infant" OR AB "maternal infant" OR SU "maternal infant") OR (TI "meal*" OR AB "meal*" OR SU "meal*") OR (TI "micronutrient*" OR AB "micronutrient*" OR SU "micronutrient*") OR (TI "mineral*" OR AB "mineral*" OR SU "mineral*") OR (TI "morbid*" OR AB "morbid*" OR SU "morbid*") OR (TI "mortal*" OR AB "mortal*" OR SU "mortal*") OR (TI "mother infant" OR AB "mother infant" OR SU "mother infant") OR (TI "neonatal development*" OR AB "neonatal development*" OR SU "neonatal development*") OR (TI "nutrient*" OR AB "nutrient*" OR SU "nutrient*") OR (TI "nutrition*" OR AB "nutrition*" OR SU "nutrition*") OR (TI "obes*" OR AB "obes*" OR SU "obes*") OR (TI "overweight" OR AB "overweight" OR SU "overweight") OR (TI "parent-child" OR AB "parent-child" OR SU "parent-child") OR (TI "parental" OR AB "parental" OR SU "parental") OR (TI "parenting" OR AB "parenting" OR SU "parenting") OR (TI "play" OR AB "play" OR SU "play") OR (TI "prevent*" OR AB "prevent*" OR SU "prevent*") OR (TI "punish*" OR AB "punish*" OR SU "punish*") OR (TI "reading" OR AB "reading" OR SU "reading") OR (TI "safe" OR AB "safe" OR SU "safe") OR (TI "safety" OR AB "safety" OR SU "safety") OR (TI "security" OR AB "security" OR SU "security") OR (TI "stunt*" OR AB "stunt*" OR SU "stunt*") OR (TI

"supervis*" OR AB "supervis*" OR SU "supervis*") OR (TI "supplement*" OR AB "supplement*" OR SU "supplement*") OR (TI "underweight" OR AB "underweight" OR SU "underweight") OR (TI "utiliz*" OR AB "utiliz*" OR SU "utiliz*") OR (TI "violence" OR AB "violence" OR SU "violence") OR (TI "psychosocial" OR AB "psychosocial" OR SU "psychosocial") OR (TI "infection*" OR AB "infection*" OR SU "infection*") OR (TI "condition*" OR AB "condition*" OR SU "condition*") OR (TI "vaccin*" OR AB "vaccin*" OR SU "vaccin*") OR (TI "disease*" OR AB "disease*" OR SU "disease*") OR (TI "disorder*" OR AB "disorder*" OR SU "disorder*") OR (TI "vitamin*" OR AB "vitamin*" OR SU "vitamin*") OR (TI "wasting" OR AB "wasting" OR SU "wasting") OR (TI "weight" OR AB "weight" OR SU "weight") OR (TI "well being" OR AB "well being" OR SU "well being") OR (TI "wellbeing" OR AB "wellbeing" OR SU "wellbeing")
**Line 5 - Combine concepts**
S1 AND S2 AND S3 AND S4
**Line 6 - Low and middle income countries**
(TI "afghan" OR AB "afghan" OR SU "afghan") OR (TI "afghanistan" OR AB "afghanistan" OR SU "afghanistan") OR (TI "albania" OR AB "albania" OR SU "albania") OR (TI "albanian" OR AB "albanian" OR SU "albanian") OR (TI "algeria" OR AB "algeria" OR SU "algeria") OR (TI "algerian" OR AB "algerian" OR SU "algerian") OR (TI "angola" OR AB "angola" OR SU "angola") OR (TI "angolan" OR AB "angolan" OR SU "angolan") OR (TI "argentina" OR AB "argentina" OR SU "argentina") OR (TI "argentinian" OR AB "argentinian" OR SU "argentinian") OR (TI "armenia" OR AB "armenia" OR SU "armenia") OR (TI "armenian" OR AB "armenian" OR SU "armenian") OR (TI "azerbaijan" OR AB "azerbaijan" OR SU "azerbaijan") OR (TI "azerbaijani" OR AB "azerbaijani" OR SU "azerbaijani") OR (TI "bajan" OR AB "bajan" OR SU "bajan") OR (TI "bangladesh" OR AB "bangladesh" OR SU "bangladesh") OR (TI "bangladeshi" OR AB "bangladeshi" OR SU "bangladeshi") OR (TI "belarus" OR AB "belarus" OR SU "belarus") OR (TI "belarusian" OR AB "belarusian" OR SU "belarusian") OR (TI "belize" OR AB "belize" OR SU "belize") OR (TI "belizean" OR AB "belizean" OR SU "belizean") OR (TI "benin" OR AB "benin" OR SU "benin") OR (TI "beninese" OR AB "beninese" OR SU "beninese") OR (TI "bhutan" OR AB "bhutan" OR SU "bhutan") OR (TI "bhutanese" OR AB "bhutanese" OR SU "bhutanese") OR (TI "bolivia" OR AB "bolivia" OR SU "bolivia") OR (TI "bolivian" OR AB "bolivian" OR SU "bolivian") OR (TI "bosnia" OR AB "bosnia" OR SU "bosnia") OR (TI "bosnian" OR AB "bosnian" OR SU "bosnian") OR (TI "botswana" OR AB "botswana" OR SU "botswana") OR (TI "brazil" OR AB "brazil" OR SU "brazil") OR (TI "brazilian" OR AB "brazilian" OR SU "brazilian") OR (TI "burkina faso" OR AB "burkina faso" OR SU "burkina faso") OR (TI "burkinabes" OR AB "burkinabes" OR SU "burkinabes") OR (TI "burmese" OR AB "burmese" OR SU "burmese") OR (TI "burundi" OR AB "burundi" OR SU "burundi") OR (TI "burundian" OR AB "burundian" OR SU "burundian") OR (TI "cambodia" OR AB "cambodia" OR SU "cambodia") OR (TI

"cambodian" OR AB "cambodian" OR SU "cambodian") OR (TI "cameroon" OR AB "cameroon" OR SU "cameroon") OR (TI "cameroonian" OR AB "cameroonian" OR SU "cameroonian") OR (TI "cape verde" OR AB "cape verde" OR SU "cape verde") OR (TI "cape verdeans" OR AB "cape verdeans" OR SU "cape verdeans") OR (TI "central african" OR AB "central african" OR SU "central african") OR (TI "central african republic" OR AB "central african republic" OR SU "central african republic") OR (TI "chad" OR AB "chad" OR SU "chad") OR (TI "chadian" OR AB "chadian" OR SU "chadian") OR (TI "china" OR AB "china" OR SU "china") OR (TI "chinese" OR AB "chinese" OR SU "chinese") OR (TI "colombia" OR AB "colombia" OR SU "colombia") OR (TI "colombian" OR AB "colombian" OR SU "colombian") OR (TI "comorian" OR AB "comorian" OR SU "comorian") OR (TI "comoros" OR AB "comoros" OR SU "comoros") OR (TI "congo" OR AB "congo" OR SU "congo") OR (TI "congolese" OR AB "congolese" OR SU "congolese") OR (TI "costa rica" OR AB "costa rica" OR SU "costa rica") OR (TI "costa rican" OR AB "costa rican" OR SU "costa rican") OR (TI "cote d'ivoire" OR AB "cote d'ivoire" OR SU "cote d'ivoire") OR (TI "cuba" OR AB "cuba" OR SU "cuba") OR (TI "cuban" OR AB "cuban" OR SU "cuban") OR (TI "djibouti" OR AB "djibouti" OR SU "djibouti") OR (TI "dominica" OR AB "dominica" OR SU "dominica") OR (TI "dominican" OR AB "dominican" OR SU "dominican") OR (TI "ecuador" OR AB "ecuador" OR SU "ecuador") OR (TI "ecuadorian" OR AB "ecuadorian" OR SU "ecuadorian") OR (TI "egypt" OR AB "egypt" OR SU "egypt") OR (TI "egyptian" OR AB "egyptian" OR SU "egyptian") OR (TI "el salvador" OR AB "el salvador" OR SU "el salvador") OR (TI "el salvadoran" OR AB "el salvadoran" OR SU "el salvadoran") OR (TI "eritrea" OR AB "eritrea" OR SU "eritrea") OR (TI "eritrean" OR AB "eritrean" OR SU "eritrean") OR (TI "eswatini" OR AB "eswatini" OR SU "eswatini") OR (TI "ethiopia" OR AB "ethiopia" OR SU "ethiopia") OR (TI "ethiopian" OR AB "ethiopian" OR SU "ethiopian") OR (TI "fiji" OR AB "fiji" OR SU "fiji") OR (TI "fijian" OR AB "fijian" OR SU "fijian") OR (TI "filipino" OR AB "filipino" OR SU "filipino") OR (TI "gabon" OR AB "gabon" OR SU "gabon") OR (TI "gabonese" OR AB "gabonese" OR SU "gabonese") OR (TI "gambia" OR AB "gambia" OR SU "gambia") OR (TI "gambian" OR AB "gambian" OR SU "gambian") OR (TI "gaza" OR AB "gaza" OR SU "gaza") OR (TI "ghana" OR AB "ghana" OR SU "ghana") OR (TI "ghanaian" OR AB "ghanaian" OR SU "ghanaian") OR (TI "grenada" OR AB "grenada" OR SU "grenada") OR (TI "grenadian" OR AB "grenadian" OR SU "grenadian") OR (TI "guatemala" OR AB "guatemala" OR SU "guatemala") OR (TI "guatemalan" OR AB "guatemalan" OR SU "guatemalan") OR (TI "guinea" OR AB "guinea" OR SU "guinea") OR (TI "guinean" OR AB "guinean" OR SU "guinean") OR (TI "haiti" OR AB "haiti" OR SU "haiti") OR (TI "haitian" OR AB "haitian" OR SU "haitian") OR (TI "honduran" OR AB "honduran" OR SU "honduran") OR (TI "honduras" OR AB "honduras" OR SU "honduras") OR (TI "india" OR AB "india" OR SU "india") OR (TI "indian" OR AB "indian" OR SU "indian") OR (TI "indonesia" OR AB "indonesia" OR SU "indonesia") OR (TI "indonesian" OR AB "indonesian" OR SU "indonesian") OR (TI "iran" OR AB "iran" OR SU "iran") OR (TI "iranian" OR AB "iranian" OR SU "iranian") OR (TI "iraq" OR AB "iraq" OR SU "iraq") OR (TI "iraqi" OR AB "iraqi" OR SU "iraqi") OR (TI "jamaica" OR AB "jamaica" OR SU "jamaica") OR (TI "jamaica" OR AB "jamaica" OR SU "jamaica") OR (TI "jamaican" OR AB "jamaican" OR SU "jamaican") OR (TI "jamaican" OR AB "jamaican" OR SU "jamaican") OR (TI "jordan" OR AB "jordan" OR SU "jordan") OR (TI "jordan" OR AB "jordan" OR SU "jordan") OR (TI "jordanian" OR AB

"jordanian" OR SU "jordanian") OR (TI "jordanian" OR AB "jordanian" OR SU "jordanian") OR (TI "kazakhstan" OR AB "kazakhstan" OR SU "kazakhstan") OR (TI "kazakhstan" OR AB "kazakhstan" OR SU "kazakhstan") OR (TI "kenya" OR AB "kenya" OR SU "kenya") OR (TI "kenya" OR AB "kenya" OR SU "kenya") OR (TI "kenyan" OR AB "kenyan" OR SU "kenyan") OR (TI "kenyan" OR AB "kenyan" OR SU "kenyan") OR (TI "kiribati" OR AB "kiribati" OR SU "kiribati") OR (TI "kiribati" OR AB "kiribati" OR SU "kiribati") OR (TI "kosovar" OR AB "kosovar" OR SU "kosovar") OR (TI "kosovar" OR AB "kosovar" OR SU "kosovar") OR (TI "kosovo" OR AB "kosovo" OR SU "kosovo") OR (TI "kosovo" OR AB "kosovo" OR SU "kosovo") OR (TI "kyrgyz" OR AB "kyrgyz" OR SU "kyrgyz") OR (TI "kyrgyz" OR AB "kyrgyz" OR SU "kyrgyz") OR (TI "kyrgyzstan" OR AB "kyrgyzstan" OR SU "kyrgyzstan") OR (TI "kyrgyzstan" OR AB "kyrgyzstan" OR SU "kyrgyzstan") OR (TI "Lao" OR AB "Lao" OR SU "Lao") OR (TI "Lao" OR AB "Lao" OR SU "Lao") OR (TI "laos" OR AB "laos" OR SU "laos") OR (TI "laos" OR AB "laos" OR SU "laos") OR (TI "laotian" OR AB "laotian" OR SU "laotian") OR (TI "laotian" OR AB "laotian" OR SU "laotian") OR (TI "lebanese" OR AB "lebanese" OR SU "lebanese") OR (TI "lebanese" OR AB "lebanese" OR SU "lebanese") OR (TI "lebanon" OR AB "lebanon" OR SU "lebanon") OR (TI "lebanon" OR AB "lebanon" OR SU "lebanon") OR (TI "lesotho" OR AB "lesotho" OR SU "lesotho") OR (TI "lesotho" OR AB "lesotho" OR SU "lesotho") OR (TI "liberia" OR AB "liberia" OR SU "liberia") OR (TI "liberia" OR AB "liberia" OR SU "liberia") OR (TI "liberian" OR AB "liberian" OR SU "liberian") OR (TI "liberian" OR AB "liberian" OR SU "liberian") OR (TI "libya" OR AB "libya" OR SU "libya") OR (TI "libya" OR AB "libya" OR SU "libya") OR (TI "libyan" OR AB "libyan" OR SU "libyan") OR (TI "libyan" OR AB "libyan" OR SU "libyan") OR (TI "macedonia" OR AB "macedonia" OR SU "macedonia") OR (TI "macedonia" OR AB "macedonia" OR SU "macedonia") OR (TI "macedonian" OR AB "macedonian" OR SU "macedonian") OR (TI "macedonian" OR AB "macedonian" OR SU "macedonian") OR (TI "madagascar" OR AB "madagascar" OR SU "madagascar") OR (TI "madagascar" OR AB "madagascar" OR SU "madagascar") OR (TI "malagasy" OR AB "malagasy" OR SU "malagasy") OR (TI "malagasy" OR AB "malagasy" OR SU "malagasy") OR (TI "malawi" OR AB "malawi" OR SU "malawi") OR (TI "malawi" OR AB "malawi" OR SU "malawi") OR (TI "malawian" OR AB "malawian" OR SU "malawian") OR (TI "malawian" OR AB "malawian" OR SU "malawian") OR (TI "malaysia" OR AB "malaysia" OR SU "malaysia") OR (TI "malaysia" OR AB "malaysia" OR SU "malaysia") OR (TI "malaysian" OR AB "malaysian" OR SU "malaysian") OR (TI "malaysian" OR AB "malaysian" OR SU "malaysian") OR (TI "maldives" OR AB "maldives" OR SU "maldives") OR (TI "maldives" OR AB "maldives" OR SU "maldives") OR (TI "maldivian" OR AB "maldivian" OR SU "maldivian") OR (TI "maldivian" OR AB "maldivian" OR SU "maldivian") OR (TI "mali" OR AB "mali" OR SU "mali") OR (TI "mali" OR AB "mali" OR SU "mali") OR (TI "malian" OR AB "malian" OR SU "malian") OR (TI "malian" OR AB "malian" OR SU "malian") OR (TI "marshall islands" OR AB "marshall islands" OR SU "marshall islands") OR (TI "marshall islands" OR AB "marshall islands" OR SU "marshall islands") OR (TI "marshallese" OR AB "marshallese" OR SU "marshallese") OR (TI "marshallese" OR AB "marshallese" OR SU "marshallese") OR (TI "mauritania" OR AB "mauritania" OR SU "mauritania") OR (TI "mauritania" OR AB "mauritania" OR SU "mauritania") OR (TI "mauritanian" OR AB "mauritanian" OR SU "mauritanian") OR (TI "mauritanian" OR AB "mauritanian" OR SU "mauritanian") OR (TI "mauritian" OR AB

"mauritian" OR SU "mauritian") OR (TI "mauritian" OR AB "mauritian" OR SU "mauritian") OR (TI "mauritius" OR AB "mauritius" OR SU "mauritius") OR (TI "mauritius" OR AB "mauritius" OR SU "mauritius") OR (TI "mexican" OR AB "mexican" OR SU "mexican") OR (TI "mexican" OR AB "mexican" OR SU "mexican") OR (TI "mexico" OR AB "mexico" OR SU "mexico") OR (TI "mexico" OR AB "mexico" OR SU "mexico") OR (TI "micronesia" OR AB "micronesia" OR SU "micronesia") OR (TI "micronesia" OR AB "micronesia" OR SU "micronesia") OR (TI "micronesian" OR AB "micronesian" OR SU "micronesian") OR (TI "micronesian" OR AB "micronesian" OR SU "micronesian") OR (TI "moldova" OR AB "moldova" OR SU "moldova") OR (TI "moldova" OR AB "moldova" OR SU "moldova") OR (TI "moldovan" OR AB "moldovan" OR SU "moldovan") OR (TI "moldovan" OR AB "moldovan" OR SU "moldovan") OR (TI "mongolia" OR AB "mongolia" OR SU "mongolia") OR (TI "mongolia" OR AB "mongolia" OR SU "mongolia") OR (TI "mongolian" OR AB "mongolian" OR SU "mongolian") OR (TI "mongolian" OR AB "mongolian" OR SU "mongolian") OR (TI "montenegrin" OR AB "montenegrin" OR SU "montenegrin") OR (TI "montenegrin" OR AB "montenegrin" OR SU "montenegrin") OR (TI "montenegro" OR AB "montenegro" OR SU "montenegro") OR (TI "montenegro" OR AB "montenegro" OR SU "montenegro") OR (TI "moroccan" OR AB "moroccan" OR SU "moroccan") OR (TI "moroccan" OR AB "moroccan" OR SU "moroccan") OR (TI "morocco" OR AB "morocco" OR SU "morocco") OR (TI "morocco" OR AB "morocco" OR SU "morocco") OR (TI "mozambican" OR AB "mozambican" OR SU "mozambican") OR (TI "mozambican" OR AB "mozambican" OR SU "mozambican") OR (TI "mozambique" OR AB "mozambique" OR SU "mozambique") OR (TI "mozambique" OR AB "mozambique" OR SU "mozambique") OR (TI "myanmar" OR AB "myanmar" OR SU "myanmar") OR (TI "myanmar" OR AB "myanmar" OR SU "myanmar") OR (TI "namibia" OR AB "namibia" OR SU "namibia") OR (TI "namibian" OR AB "namibian" OR SU "namibian") OR (TI "nepal" OR AB "nepal" OR SU "nepal") OR (TI "nepalese" OR AB "nepalese" OR SU "nepalese") OR (TI "nicaragua" OR AB "nicaragua" OR SU "nicaragua") OR (TI "nicaraguan" OR AB "nicaraguan" OR SU "nicaraguan") OR (TI "niger" OR AB "niger" OR SU "niger") OR (TI "nigeria" OR AB "nigeria" OR SU "nigeria") OR (TI "nigerian" OR AB "nigerian" OR SU "nigerian") OR (TI "north korea" OR AB "north korea" OR SU "north korea") OR (TI "north korea" OR AB "north korea" OR SU "north korea") OR (TI "north korean" OR AB "north korean" OR SU "north korean") OR (TI "north korean" OR AB "north korean" OR SU "north korean") OR (TI "pakistan" OR AB "pakistan" OR SU "pakistan") OR (TI "pakistani" OR AB "pakistani" OR SU "pakistani") OR (TI "palau" OR AB "palau" OR SU "palau") OR (TI "palauan" OR AB "palauan" OR SU "palauan") OR (TI "panama" OR AB "panama" OR SU "panama") OR (TI "panamanian" OR AB "panamanian" OR SU "panamanian") OR (TI "papua new guinea" OR AB "papua new guinea" OR SU "papua new guinea") OR (TI "papua new guinean" OR AB "papua new guinean" OR SU "papua new guinean") OR (TI "paraguay" OR AB "paraguay" OR SU "paraguay") OR (TI "paraguayan" OR AB "paraguayan" OR SU "paraguayan") OR (TI "peru" OR AB "peru" OR SU "peru") OR (TI "peruvian" OR AB "peruvian" OR SU "peruvian") OR (TI "philippines" OR AB "philippines" OR SU "philippines") OR (TI "principe" OR AB "principe" OR SU "principe") OR (TI "romania" OR AB "romania" OR SU "romania") OR (TI "romanian" OR AB "romanian" OR SU "romanian") OR (TI "russia" OR AB "russia" OR SU "russia") OR (TI "russian" OR AB "russian" OR SU "russian") OR (TI "rwanda" OR AB "rwanda" OR SU "rwanda") OR (TI

"rwandan" OR AB "rwandan" OR SU "rwandan") OR (TI "saint kitts" OR AB "saint kitts" OR SU "saint kitts") OR (TI "saint lucia" OR AB "saint lucia" OR SU "saint lucia") OR (TI "saint vincent" OR AB "saint vincent" OR SU "saint vincent") OR (TI "salvadoran" OR AB "salvadoran" OR SU "salvadoran") OR (TI "samoa" OR AB "samoa" OR SU "samoa") OR (TI "samoan" OR AB "samoan" OR SU "samoan") OR (TI "santomea" OR AB "santomea" OR SU "santomea") OR (TI "sao tome" OR AB "sao tome" OR SU "sao tome") OR (TI "senegal" OR AB "senegal" OR SU "senegal") OR (TI "senegalese" OR AB "senegalese" OR SU "senegalese") OR (TI "serbia" OR AB "serbia" OR SU "serbia") OR (TI "serbian" OR AB "serbian" OR SU "serbian") OR (TI "seychelles" OR AB "seychelles" OR SU "seychelles") OR (TI "seychellois" OR AB "seychellois" OR SU "seychellois") OR (TI "sierra leone" OR AB "sierra leone" OR SU "sierra leone") OR (TI "sierra leoneans" OR AB "sierra leoneans" OR SU "sierra leoneans") OR (TI "solomon islander" OR AB "solomon islander" OR SU "solomon islander") OR (TI "solomon islands" OR AB "solomon islands" OR SU "solomon islands") OR (TI "somalia" OR AB "somalia" OR SU "somalia") OR (TI "somalian" OR AB "somalian" OR SU "somalian") OR (TI "south africa" OR AB "south africa" OR SU "south africa") OR (TI "south african" OR AB "south african" OR SU "south african") OR (TI "sri lanka" OR AB "sri lanka" OR SU "sri lanka") OR (TI "sri lankan" OR AB "sri lankan" OR SU "sri lankan") OR (TI "sudan" OR AB "sudan" OR SU "sudan") OR (TI "sudanese" OR AB "sudanese" OR SU "sudanese") OR (TI "suriname" OR AB "suriname" OR SU "suriname") OR (TI "swazi" OR AB "swazi" OR SU "swazi") OR (TI "swaziland" OR AB "swaziland" OR SU "swaziland") OR (TI "syria" OR AB "syria" OR SU "syria") OR (TI "syrian" OR AB "syrian" OR SU "syrian") OR (TI "tadzhik" OR AB "tadzhik" OR SU "tadzhik") OR (TI "tajik" OR AB "tajik" OR SU "tajik") OR (TI "tajikistan" OR AB "tajikistan" OR SU "tajikistan") OR (TI "tanzania" OR AB "tanzania" OR SU "tanzania") OR (TI "tanzanian" OR AB "tanzanian" OR SU "tanzanian") OR (TI "thai" OR AB "thai" OR SU "thai") OR (TI "thailand" OR AB "thailand" OR SU "thailand") OR (TI "timor" OR AB "timor" OR SU "timor") OR (TI "togo" OR AB "togo" OR SU "togo") OR (TI "togolese" OR AB "togolese" OR SU "togolese") OR (TI "tonga" OR AB "tonga" OR SU "tonga") OR (TI "tongan" OR AB "tongan" OR SU "tongan") OR (TI "tunisia" OR AB "tunisia" OR SU "tunisia") OR (TI "tunisian" OR AB "tunisian" OR SU "tunisian") OR (TI "turkey" OR AB "turkey" OR SU "turkey") OR (TI "turkish" OR AB "turkish" OR SU "turkish") OR (TI "turkmen" OR AB "turkmen" OR SU "turkmen") OR (TI "turkmenistan" OR AB "turkmenistan" OR SU "turkmenistan") OR (TI "tuvalu" OR AB "tuvalu" OR SU "tuvalu") OR (TI "tuvaluans" OR AB "tuvaluans" OR SU "tuvaluans") OR (TI "uganda" OR AB "uganda" OR SU "uganda") OR (TI "ugandan" OR AB "ugandan" OR SU "ugandan") OR (TI "ukraine" OR AB "ukraine" OR SU "ukraine") OR (TI "ukrainian" OR AB "ukrainian" OR SU "ukrainian") OR (TI "uzbek" OR AB "uzbek" OR SU "uzbek") OR (TI "uzbekistan" OR AB "uzbekistan" OR SU "uzbekistan") OR (TI "vanuatu" OR AB "vanuatu" OR SU "vanuatu") OR (TI "venezuela" OR AB "venezuela" OR SU "venezuela") OR (TI "venezuelan" OR AB "venezuelan" OR SU "venezuelan") OR (TI "vietnam" OR AB "vietnam" OR SU "vietnam") OR (TI "vietnamese" OR AB "vietnamese" OR SU "vietnamese") OR (TI "west bank" OR AB "west bank" OR SU "west bank") OR (TI "yemen" OR AB "yemen" OR SU "yemen") OR (TI "yemeni" OR AB "yemeni" OR SU "yemeni") OR (TI "yemenite" OR AB "yemenite" OR SU "yemenite") OR (TI "zambia" OR AB "zambia" OR SU "zambia") OR (TI "zambian" OR AB "zambian" OR SU "zambian") OR (TI

"zimbabwe" OR AB "zimbabwe" OR SU "zimbabwe") OR (TI "zimbabwean" OR AB "zimbabwean" OR SU "zimbabwean")
**Line 7 - High income countries**
(TI "Alabama" OR AB "Alabama" OR MW "Alabama") OR (TI "Alaska" OR AB "Alaska" OR MW "Alaska") OR (TI "American Samoa" OR AB "American Samoa" OR MW "American Samoa") OR (TI "Andorra" OR AB "Andorra" OR MW "Andorra") OR (TI ""Antigua and Barbuda"" OR AB ""Antigua and Barbuda"" OR MW ""Antigua and Barbuda"") OR (TI "Arizona" OR AB "Arizona" OR MW "Arizona") OR (TI "Arkansas" OR AB "Arkansas" OR MW "Arkansas") OR (TI "Aruba" OR AB "Aruba" OR MW "Aruba") OR (TI "Australia" OR AB "Australia" OR MW "Australia") OR (TI "Austria" OR AB "Austria" OR MW "Austria") OR (TI "Bahamas" OR AB "Bahamas" OR MW "Bahamas") OR (TI "Bahrain" OR AB "Bahrain" OR MW "Bahrain") OR (TI "Barbados" OR AB "Barbados" OR MW "Barbados") OR (TI "Belgium" OR AB "Belgium" OR MW "Belgium") OR (TI "Bermuda" OR AB "Bermuda" OR MW "Bermuda") OR (TI "Brunei" OR AB "Brunei" OR MW "Brunei") OR (TI "Bulgaria" OR AB "Bulgaria" OR MW "Bulgaria") OR (TI "California" OR AB "California" OR MW "California") OR (TI "Canada" OR AB "Canada" OR MW "Canada") OR (TI "Cayman Islands" OR AB "Cayman Islands" OR MW "Cayman Islands") OR (TI "Channel Islands" OR AB "Channel Islands" OR MW "Channel Islands") OR (TI "Chile" OR AB "Chile" OR MW "Chile") OR (TI "Colorado" OR AB "Colorado" OR MW "Colorado") OR (TI "Connecticut" OR AB "Connecticut" OR MW "Connecticut") OR (TI "Croatia" OR AB "Croatia" OR MW "Croatia") OR (TI "Curacao" OR AB "Curacao" OR MW "Curacao") OR (TI "Cyprus" OR AB "Cyprus" OR MW "Cyprus") OR (TI "Czech*" OR AB "Czech*" OR MW "Czech*") OR (TI "Czechia" OR AB "Czechia" OR MW "Czechia") OR (TI "Delaware" OR AB "Delaware" OR MW "Delaware") OR (TI "Denmark" OR AB "Denmark" OR MW "Denmark") OR (TI "District of Columbia" OR AB "District of Columbia" OR MW "District of Columbia") OR (TI "england" OR AB "england" OR MW "england") OR (TI "Estonia" OR AB "Estonia" OR MW "Estonia") OR (TI "Faroe Islands" OR AB "Faroe Islands" OR MW "Faroe Islands") OR (TI "Finland" OR AB "Finland" OR MW "Finland") OR (TI "Florida" OR AB "Florida" OR MW "Florida") OR (TI "France" OR AB "France" OR MW "France") OR (TI "French Polynesia" OR AB "French Polynesia" OR MW "French Polynesia") OR (TI "Georgia" OR AB "Georgia" OR MW "Georgia") OR (TI "Germany" OR AB "Germany" OR MW "Germany") OR (TI "Gibraltar" OR AB "Gibraltar" OR MW "Gibraltar") OR (TI "Greece" OR AB "Greece" OR MW "Greece") OR (TI "Greenland" OR AB "Greenland" OR MW "Greenland") OR (TI "Guam" OR AB "Guam" OR MW "Guam") OR (TI "Guyana" OR AB "Guyana" OR MW "Guyana") OR (TI "Hawaii" OR AB "Hawaii" OR MW "Hawaii") OR (TI "Hong Kong" OR AB "Hong Kong" OR MW "Hong Kong") OR (TI "Hungary" OR AB "Hungary" OR MW "Hungary") OR (TI "Iceland*" OR AB "Iceland*" OR MW "Iceland*") OR (TI "Idaho*" OR AB "Idaho*" OR MW "Idaho*") OR (TI "Illinois*" OR AB "Illinois*" OR MW "Illinois*") OR (TI "Indiana*" OR AB "Indiana*" OR MW "Indiana*") OR (TI "Iowa*" OR AB "Iowa*" OR MW "Iowa*") OR (TI "Ireland" OR AB "Ireland" OR MW "Ireland") OR (TI "Isle of Man" OR AB "Isle of Man" OR MW "Isle of Man") OR (TI "Israel" OR AB "Israel" OR MW "Israel") OR (TI "Italy"

OR AB "Italy" OR MW "Italy") OR (TI "Japan" OR AB "Japan" OR MW "Japan") OR (TI "Kansas" OR AB "Kansas" OR MW "Kansas") OR (TI "Kentucky" OR AB "Kentucky" OR MW "Kentucky") OR (TI "Korea" OR AB "Korea" OR MW "Korea") OR (TI "Kuwait" OR AB "Kuwait" OR MW "Kuwait") OR (TI "Latvia" OR AB "Latvia" OR MW "Latvia") OR (TI "Liechtenstein" OR AB "Liechtenstein" OR MW "Liechtenstein") OR (TI "Lithuania" OR AB "Lithuania" OR MW "Lithuania") OR (TI "Louisiana*" OR AB "Louisiana*" OR MW "Louisiana*") OR (TI "Luxembourg" OR AB "Luxembourg" OR MW "Luxembourg") OR (TI "Macao" OR AB "Macao" OR MW "Macao") OR (TI "Maine" OR AB "Maine" OR MW "Maine") OR (TI "Malta" OR AB "Malta" OR MW "Malta") OR (TI "Maryland*" OR AB "Maryland*" OR MW "Maryland*") OR (TI "Massachusetts" OR AB "Massachusetts" OR MW "Massachusetts") OR (TI "Michigan" OR AB "Michigan" OR MW "Michigan") OR (TI "Minnesota" OR AB "Minnesota" OR MW "Minnesota") OR (TI "Mississippi" OR AB "Mississippi" OR MW "Mississippi") OR (TI "Missouri" OR AB "Missouri" OR MW "Missouri") OR (TI "Monaco" OR AB "Monaco" OR MW "Monaco") OR (TI "Montana" OR AB "Montana" OR MW "Montana") OR (TI "Nauru" OR AB "Nauru" OR MW "Nauru") OR (TI "Nebraska" OR AB "Nebraska" OR MW "Nebraska") OR (TI "Netherlands" OR AB "Netherlands" OR MW "Netherlands") OR (TI "Nevada" OR AB "Nevada" OR MW "Nevada") OR (TI "New Caledonia" OR AB "New Caledonia" OR MW "New Caledonia") OR (TI "New Hampshire" OR AB "New Hampshire" OR MW "New Hampshire") OR (TI "New Jersey" OR AB "New Jersey" OR MW "New Jersey") OR (TI "New Mexico" OR AB "New Mexico" OR MW "New Mexico") OR (TI "New York" OR AB "New York" OR MW "New York") OR (TI "New Zealand" OR AB "New Zealand" OR MW "New Zealand") OR (TI "North Carolina" OR AB "North Carolina" OR MW "North Carolina") OR (TI "North Dakota" OR AB "North Dakota" OR MW "North Dakota") OR (TI "Northern Mariana Islands" OR AB "Northern Mariana Islands" OR MW "Northern Mariana Islands") OR (TI "Norway" OR AB "Norway" OR MW "Norway") OR (TI "Ohio" OR AB "Ohio" OR MW "Ohio") OR (TI "Oklahoma" OR AB "Oklahoma" OR MW "Oklahoma") OR (TI "Oman" OR AB "Oman" OR MW "Oman") OR (TI "Oregon" OR AB "Oregon" OR MW "Oregon") OR (TI "Palau" OR AB "Palau" OR MW "Palau") OR (TI "Panama" OR AB "Panama" OR MW "Panama") OR (TI "Pennsylvania" OR AB "Pennsylvania" OR MW "Pennsylvania") OR (TI "Poland" OR AB "Poland" OR MW "Poland") OR (TI "Portugal" OR AB "Portugal" OR MW "Portugal") OR (TI "Puerto Rico" OR AB "Puerto Rico" OR MW "Puerto Rico") OR (TI "Qatar" OR AB "Qatar" OR MW "Qatar") OR (TI "Rhode Island" OR AB "Rhode Island" OR MW "Rhode Island") OR (TI "Romania" OR AB "Romania" OR MW "Romania") OR (TI "Russia" OR AB "Russia" OR MW "Russia") OR (TI "Russian" OR AB "Russian" OR MW "Russian") OR (TI ""Saint Kitts and Nevis"" OR AB ""Saint Kitts and Nevis"" OR MW ""Saint Kitts and Nevis"") OR (TI "Saint Martin" OR AB "Saint Martin" OR MW "Saint Martin") OR (TI "San Marino" OR AB "San Marino" OR MW "San Marino") OR (TI "Saudi Arabia" OR AB "Saudi Arabia" OR MW "Saudi Arabia") OR (TI "scotland" OR AB "scotland" OR MW "scotland") OR (TI "Seychelles" OR AB "Seychelles" OR MW "Seychelles") OR (TI "Singapore" OR AB "Singapore" OR MW "Singapore") OR (TI "Sint Maarten" OR AB "Sint Maarten" OR MW "Sint Maarten") OR (TI "Slovak Republic" OR AB "Slovak Republic" OR MW "Slovak Republic") OR (TI "Slovakia" OR AB "Slovakia" OR MW "Slovakia") OR (TI "Slovenia" OR AB "Slovenia" OR MW "Slovenia") OR (TI "South Carolina" OR AB "South Carolina" OR MW "South Carolina") OR (TI "South Dakota" OR AB "South

Dakota" OR MW "South Dakota") OR (TI "Spain" OR AB "Spain" OR MW "Spain") OR (TI "St Martin" OR AB "St Martin" OR MW "St Martin") OR (TI "Sweden" OR AB "Sweden" OR MW "Sweden") OR (TI "Switzerland" OR AB "Switzerland" OR MW "Switzerland") OR (TI "Taiwan" OR AB "Taiwan" OR MW "Taiwan") OR (TI "Tennessee" OR AB "Tennessee" OR MW "Tennessee") OR (TI "Texas" OR AB "Texas" OR MW "Texas") OR (TI "Tobago" OR AB "Tobago" OR MW "Tobago") OR (TI "Trinidad" OR AB "Trinidad" OR MW "Trinidad") OR (TI ""Turks and Caicos"" OR AB ""Turks and Caicos"" OR MW ""Turks and Caicos"") OR (TI "United Arab Emirates" OR AB "United Arab Emirates" OR MW "United Arab Emirates") OR (TI "United Kingdom" OR AB "United Kingdom" OR MW "United Kingdom") OR (TI "United States" OR AB "United States" OR MW "United States") OR (TI "Uruguay" OR AB "Uruguay" OR MW "Uruguay") OR (TI "Utah" OR AB "Utah" OR MW "Utah") OR (TI "Vermont" OR AB "Vermont" OR MW "Vermont") OR (TI "Virgin Islands" OR AB "Virgin Islands" OR MW "Virgin Islands") OR (TI "Virginia" OR AB "Virginia" OR MW "Virginia") OR (TI "wales" OR AB "wales" OR MW "wales") OR (TI "Washington" OR AB "Washington" OR MW "Washington") OR (TI "West Virginia" OR AB "West Virginia" OR MW "West Virginia") OR (TI "Wisconsin" OR AB "Wisconsin" OR MW "Wisconsin") OR (TI "Wyoming" OR AB "Wyoming" OR MW "Wyoming") OR (TI "Alberta" OR AB "Alberta" OR MW "Alberta") OR (TI "British Columbia" OR AB "British Columbia" OR MW "British Columbia") OR (TI "Manitoba" OR AB "Manitoba" OR MW "Manitoba") OR (TI "New Brunswick" OR AB "New Brunswick" OR MW "New Brunswick") OR (TI "Newfoundland" OR AB "Newfoundland" OR MW "Newfoundland") OR (TI "Northwest Territories" OR AB "Northwest Territories" OR MW "Northwest Territories") OR (TI "Nova Scotia" OR AB "Nova Scotia" OR MW "Nova Scotia") OR (TI "Nunavut" OR AB "Nunavut" OR MW "Nunavut") OR (TI "Ontario" OR AB "Ontario" OR MW "Ontario") OR (TI "Prince Edward Island" OR AB "Prince Edward Island" OR MW "Prince Edward Island") OR (TI "Quebec" OR AB "Quebec" OR MW "Quebec") OR (TI "Saskatchewan" OR AB "Saskatchewan" OR MW "Saskatchewan") OR (TI "Yukon Territory" OR AB "Yukon Territory" OR MW "Yukon Territory") OR (SU "Canada+") OR (SU "United States+") OR (SU "United Kingdom+") OR (SU "Australia+")
**Line 8 - filter out LMICs unless they also mention HICs**
S5 not (S6 not S7)
Used platform options to filter to:<br>2000-2024<br>English

Additional sources

Homelessness Impact: Evidence Finder

https://www.homelessnessimpact.org/
The Evidence Finder map was used over other tools available on this site because they included filters for Children & Families, as well as Health Outcomes. The map interface was not ideal, however.

After application of the filters, each area of the map was zoomed in and individual studies were opened in new tabs. Zotero software [Roy Rosenzweig Center for History and New Media. (2016) Zotero [Computer software]] browser connector to import the studies.

Three hundred seventeen articles were imported into EndNote from the Evidence Finder (1/2025). These were filtered by searching within the record for terms relevant to co-development. These included: participatory, "action research," "citizen scien," "co-," " co-," partner, collabora, code, copro, cocre. No articles included both youth and co-dev terms. No studies were uploaded for screening.

Collaborative Indigenous Research Digital Garden

https://www.oise.utoronto.ca/collaborativeindigenousresearch/research

The tag "Children and Families" was used to limit results before downloading. One hundred thirteen studies were downloaded (1/2025) and then uploaded to a spreadsheet. These were filtered by searching for terms in any part of the record relevant to health or housing/homelessness, including health, nutrition, wellbeing, welfare, care, medical, medicine, therapy, housing, housed, homeless. Then results were filtered by country or region. Forty records were retained after filtering. Six of these were excluded because they were not research articles or reports. Thirty-four results were uploaded for screening.

Health Cascade Co-Creation Database

Loisel, Q., Agnello, D., & Chastin, S. (2022). Co-Creation Database (2.0) [Data set]. Zenodo. https://doi.org/10.5281/zenodo.7849501

The Co-Creation Database includes 52,821 references, which were downloaded as a CSV and opened in a spreadsheet.

Records were first filtered for children/family terms in the title and abstract: child, youth, teens, teenag, teen (title only), adolesc, family, families, parent, mother, father, pregnan, infant, young people

After this filter, 9358 rows were retained for having at least 1 of these terms in the title or abstract. These were uploaded to EndNote, where 880 duplicates were removed (from within Co-creation Database results, as well as with already-retrieved records).

Records were then tagged with homelessness or health based on searches of the following keywords:

- Homelessness: homeless, housed, soup kitchen, squat, sofa surf, slum, shelter, runaway, settlement, refugee, migrant, food pantry, evict, farmworker, farm worker, encamp, displace, camps, street, housing

- Health: health, disease, illness, nutrition, diet, psychological, psychosocial, wellbeing, well-being, well being, emotional, child development, adolescent development, youth development, suicide, substance use, substance abuse, drug use, drug abuse, physical activity, overweight, body weight, obesity, asthma, parent child, mother child, mother infant, quality of life, mortality, safe, violen, abuse, neglect, risk behav, risky behav, risk taking, child care, childcare, childbirth, breast, infection, hiv, sexual, tobacco, smoking, drinking, alcohol, prevent (title), medic, disorder, meals, food, disabled, disabilit, education_, family planning, contracept

Some records were weeded out based on location (LMICs) and age of participants (some were for elderly). Two hundred six records were tagged with both homelessness and health (from the initial set that had already been filtered for children/families). These (206 results) were uploaded for screening.

Google Scholar search

- Google Scholar was searched with the following strategy February 2025:
  - (codevelop OR codesign OR coproduce OR cocreate OR coresearch) (homeless OR homelessness OR migrant) (children OR family OR youth OR mother)
- The search was implemented using Apify.com with the following scraper: marco.gullo/google-scholar-scraper. This allowed links and texts from results to be downloaded into a CSV file, rather than requiring a manual review within the Google Scholar interface.
  - Default settings were used except for the following: "Search keyword" (codevelop OR codesign OR coproduce OR cocreate OR coresearch) (homeless OR homelessness OR migrant) (children OR family OR adolescents OR youth OR parent OR mothers); Max items: 1000; Newer than: 2000. Input JSON code pasted below:


```
{
 "enableDebugDumps": false,
 "filter": "all",
 "keyword": "(codevelop OR codesign OR coproduce OR cocreate OR
coresearch) (homeless OR homelessness OR migrant) (children OR family OR
adolescents OR youth OR parent OR mothers)",
 "maxItems": 1000,
 "newerThan": 2000,
 "proxyOptions": {
   "useApifyProxy": true,
   "apifyProxyGroups": []
 },
 "sortBy": "relevance",
 "articleType": "any"
}
```


- Results were downloaded from Apify as a CSV and uploaded to a spreadsheet. For each search result, the data included the title, authors, all or part of the source (journal) title, snippets of text where matches were found with the search terms, the type of results (article, citation, patent), main link to the search result and additional link to a PDF or HTML version if applicable.
- Conditional formatting was used to identify duplicate results and to identify results with keywords matching the search terms. Results were tagged with the concepts of "co-development", "homelessness", and "child/families" if the search terms for those concepts were found in the search result (mainly in the title of the result or in the matching text snippets).

- Results with all three concepts present were first reviewed manually, and out-of-scope materials were excluded. For example, studies clearly taking place in lower-income countries, which focused on non-health-related issues or which focused on populations not explicitly indicated to be homeless, migrants, or having housing issues, were excluded at this point. The remaining results were uploaded to Covidence for screening.
- 995 results were uploaded to a spreadsheet; 442 results were manually reviewed; 52 were uploaded to Covidence for screening

Table S3. Additional Records

Source/Step	*# 12/2024*	Notes
PubMed	597	
Embase	662	
PsycInfo	342	
SocIndex	186	
Eric/Education	250	
Global Health	251	
Cinahl	491	
**Total from databases**	**2779**	1/2025-2/2025
Other sources	292	*34 crcl*<br>*52 google scholar*<br>*206 cascade*
**Total retrieved**	**3071**	
Duplicates (endnote + cov + manual)	1285	1239 endnote<br>43 covidence<br>3 manual
Filtered in Endnote (reviews, reply, commentary, editorial, etc)	169	128 reviews<br>41 other
**Total title/abstract screen**	**1617 in cov**	
Removed, title/abstract screen	1521	
**Total full-text review**	**96**	
Removed, full-text review	87 total<br>For PRISMA diagram	**Full data on Reasons for Exclusions below***

	23 = two or more reasons for exclusion<br>21 = not able to distinguish population of interest or population not homeless<br>15 = not co developed with population<br>12 = no intervention<br>7 = population not children or families<br>5 = not original research<br>4 = wrong publication type	
**Reports/studies included in review**	**9**	

*Reasons for Exclusion	Number of Excluded Articles
No way to distinguish population of interest from broader population	8
Interventions not co-developed with the population of interest (children and families)	29
Not an intervention, program, or strategy	20
No children under 18 yrs old or families of such children	15
Not original research (i.e., commentary, editorial, review)	6
Wrong publication types (i.e., meeting abstracts, dissertations)	4
No way to distinguish housing status, or did not fit the McKinney-Vento definition	30
**The sum of excluded articles per reason**	**112**

Table S4. Data Extraction Sheet Example

Reviewer	Covidence ID	Author	Year	Country/ State/ City of Study	Study Quality	Study Type	N	Population	Inclusion Criteria	Exclusion Criteria	Study Setting	Age of Children	Gender %	Ethnicity %	Mean Income	Context	Intervention Description	Intervention Type	Intervention Components	Intervention Delivery Mode	Provider of the Intervention	Primary Outcomes	Secondary Outcomes	Outcome Results
JK	1016	Cawley	2022	USA	poor	Qualitative (Cas	not specified	children K-5 and their caregiv	co-developed with families experiencing homelessness		Shelters	0-5 Y	not specified	not specificed	not specified	Transit...	repeat check-ins, collaborative reflection, understandin science programs based on family interests for	Socio-structural	Mental Health S	In Person Wo	Museum Staff	Social Support	Quality of Life	Intervention helped combat stigma about homelessness by working towards making parents and children feel welcome, safe, heard, and wanted
SL	1016	Cawley	2022	USA	Poor	Qualit...	NA	Families stay temporarily at th into more permanent housing	- Children under 18 years-old or families of such childre - Experiencing homelessness, a lack of stable, safe, ar		Shelters	0-5 Y	NA	NA	NA	Transit...	Educational Science Programs	Socio-structural	Mental Health S	In Person Wo	Museum Staff	Social Support	Quality of Life	Social connection and engagement was positive
JK	1026	Cumming	2022	UK	fair	Mixed-methods	195	Youth experiencing homeless accommodation (e.g., 6–12 m EET (as opposed to NEET yo in work due to disability or he of EET, or identified by frontli from a group-based program	Intervention is co-developed with population of interest		Comm...	13-18 Y	Not specified	Not specified	not specified	Transit...	My Strengths Training for Life™ (MST4Life™), experiential psychoeducational program based	Behavioral	Mental Health S	In Person	Social Worker	Social Support	Mental Health	Youth significantly improved their resilience and well-being, and mental skills development, were better mental as well as engage in wider services such as to address alcohol misuse. Evidence in support of improved longer-term outcomes includes MST4Life™ participants being two times more likely to transition into EET and independent living as compared to standard care by the Housing Service (pg 5) ; Fidelity assessment mainly focused on delivery (e.g., number of sessions delivered) and
SL	1026	Cumming	2022	UK	Fair	Qualit...	195	young people experiencing h aged 16–24 years old and livi accommodation (e.g., 6–12 m EET (as opposed to NEET yo in work due to disability or he of EET, or identified by frontli from a group-based program	- Children under 18 years-old or families of such childre - Experiencing homelessness, a lack of stable, safe, ar		Comm...	13-18 Y	NA	NA	NA	Transit...	My Strengths Training for Life™ (MST4Life™), experiential psychoeducational program	Behavioral Sc	Mental Health S	In Person	Social Worker	Social Support	Mental Health	Engagement in sessions tended to be high, with mean facilitator ratings of 8.24 (SD = 1.16). The average score on Interest/Enjoyment subscale was 4.34 (n = 75; SD = 0.82), based on a scale ranging from 1 (not true at all) to 5 (very true). Overall, MST4Life™ was found to have been deliv high fidelity across a range of facilitators (i.e., high levels of needs supportive behaviors and low levels of needs thwarting behaviors). University staff with a psychology background and greater experience in delivering the program adhered more to the desired delivery style than frontline staff from the Service who had a mixture of backgrounds and educational levels, as well as less experience in delivering the program. Facilitators experienced challenges and enablers to delivering this intervention with fidelity, including high support needs, language barriers, drug use which the program is delivered, such as communication with staff and availability of training spaces.
JK	474	Gewirtz O'Brien	2022	USA	Fair	Qualit...	17	Youth and youth parents livin	Intervention was co-devel	68.8% of youth were adults (	Shelters	13-18 Y	76% female iden	Not specified	not specified	Transit...	Empowering Parents for Wellness in S of youth parents and their children, an unplanned pregnancies. It consisted c erment program for youth based on th of youth, 2) training and ongoing supp regarding adolescent health, and 3) sl services	Behavioral	Healthcare Acc	In Person	Healthcare Pro	Physical Health	Mental Health	"The application of frameworks from implementation science allowed us to select of the youth and the partnering community organization"
SL	474	Gewirtz O'Brien	2022	USA	Fair	Qualit...	17	Pregnant youth experiencing	- Children under 18 years-old or families of such childre - Experiencing homelessness, a lack of stable, safe, ar		Comm...	13-18 Y	76.5% F; 17.6%	NA	NA	Transit...	e delivery of adolescent health curricula	Behavioral	Healthcare Acc	In Person	Healthcare Pro	Physical Health	Mental Health	suggestions for a multi-pronged approach, program staff engagement, and the need for health care access; Rights, Respect, and Responsibility curriculum from Advocates for Youth as the basis for the group-based intervention for youth and tailored the intervention to address identif
JK	395	Marziale	2024	UK	Poor	not reported	124	Adult women from migrant ba	Intervention is co-developed with population of interest		Comm...	Unspe...	100% women id	Not specified	not specified	Transit...	#NoticeUs campaign to bring awarene	Socio-structural	Mental Health S	In Person Wo	Family Advocat	Social Support	Quality of Life	Through the training and leadership activities, all the women felt more con- dent and empowered in applying skills developed together for community-led solutions. The women felt motivated to continue to participate in their communities on key social issues and to mobilise community members into community action for change.(pg 323)
SL	395	Marziale	2024	UK, London	Poor	Qualit...	124	Migrant women who have bee	- Children under 18 years - Experiencing homelessr	- Original Research	Comm...	Unspe...	100% Women	NA	NA	Transit...	Art and Theatre based intervention to	Socio-structural	Mental Health S	In Person Wo	Family Advocat	Social Support	Quality of Life	Intervention already tested; main outcome reported was policy initiative and partic
JK	475	McKay	2010	USA	Poor	RCT	MFG: 190, HOP	MFG: inner city youth and the	Interventions are co-developed with population of inter		Comm...	11-14	Not specified	Not specified, but emp	Not specified	Transit...	MFG: mental health service interventi	Behavioral	Mental Health S	In Person Wo	Family Advocat	Mental Health	Quality of Life	Preliminary results for each project indicate significant reductions in youth mental health symptoms and illustrate that these interventions are a feasible alter traditional individualized outpatient psychotherapy. Furthermore, these projects illu that effective services can be delivered not only in urban outpatient child mental h clinics, where the MFG program has been integrated into standard care, but also i of settings such as schools and shelters serving homeless families. The results also support the notion that collaborative efforts lead to "building bridg link vulnerable youth and their families to much needed mental health care pg 6
SL	475	McKay	2010	USA	Poor	Qualit...	MFG: 190, HOP	Youth and caretakers experie	Interventions are co-deve	NA	Shelters	11-14	NA	NA	NA	Transit...	eight-session's curriculum in which pa separately and also together to discus monitoring and supervision, self-respe Concepts such as self-efficacy and co communication are integrated into ea	Behavioral	Mental Health S	In Person Wo	Family Advocat	Mental Health	Quality of Life	In addition, there is emerging preliminary evidence that HOPE is associated with s reductions in youth mental health symptoms
JK	68	Cumming 2022 ((MST4Life™) Pr Experiencing Ho	2022	UK	Poor	Mixed-methods	15	Current youth experiencing h	Intervention is co-developed with population of interest		Homes	16-24	60% male, 40%	Black (e.g., African, C British) 46.7 (7) White (e.g., British, Iri Traveler, or other Whi Mixed (i.e., multiple et	NA	Chronic	MST4Life™ is an experiential and strengths-ba tended to promote the use of mental skills in yo by better self-regulating their thoughts, feelings these skills to other life domains (e.g., educatio	Behavioral	Mental Health S	In Person	Social Worker	Social Support	Quality of Life	Feasibility was indicated via the themes of attendance, engagement, and reaction. The findir young people enjoyed and perceived a need for the program, that they considered its evaluation methods to be acceptable, and that both the community-based and residential phases could be implemented as planned. Minor modifications are nee strategies before it is more widely rolled out and evaluated
SL	68	Cumming 2022 ((MST4Life™) Pr Experiencing Ho	2022	UK	Poor	Qualit...	15: 15 young pe	Young people were invited to participate in the pilot MST (i.e., where young people typi by their support worker due to training opportunities (e.g., w employment, or training oppo	Interventionns are co-dev	NA	Homes	16-24	Of the young pe	Black (e.g., African, C British) 46.7 (7) White (e.g., British, Iri Traveler, or other Whi Mixed (i.e., multiple et	NA	Chronic	My Strengths Training for Life™ (MST improving wellbeing and social inclusi addresses a gap in strengths-based p in vulnerable older adolescents/emerg apply, further develop and transfer us different contexts	Behavioral	Mental Health S	In Person	Social Worker	Social Support	Quality of Life	High level of attendance; Positive engagement; Feasibility was indicated via the themes of attendance, engagement, and reaction. The findir young people enjoyed and perceived a need for the program, that they considered its evaluation methods to be acceptable, and that both the community-based and residential phases could be implemented as planned. Minor modifications are nee strategies before it is more widely rolled out and evaluated logic model by demons
DMR	521	Holtrop	2015	USA, North Florida	Good/Fair	Qualitati ve	40	parents and primary caregivers residing in transitional housing "typically with their children"	required to: (a) reside at the transitional housing community at the time of the interview, (b) be a parent or primary caregiver, and (c) have a child under the age of 18.	While no additional exclusion criteria were applied for this study, individuals with a severe mental illness (e.g., schizophrenia), active substance abuse problem, or history of sexual offense are typically referred elsewhere to more appropriate services by the housing program.	Commun ity	0-18 Y	31 biological mothers, eight biological fathers, and one nonbiological male caregiver from a total of 33 different families	50.0% White and 47.5% African American (with one additional participant self-identifying as "other")	Not specified	Transitio nal	No intervention delivered yet; intervention components informed by qualitative interviews with parents and caregivers; seven categories describe specific content areas that could inform the curriculum of a parenting intervention for parents in transitional housing.	Combination	Multiple co...	In Person	Program S...			NA-intervention not delivered yet
JK	521	Holtrop	2015	USA (North Flori	Good/fair	Qualit...	40	Parents with children under 1	Participants must: (a) resi tional housing community or primary caregiver, and	While no additional exclusion criter with a severe mental illness abuse problem, or history of elsewhere to more appropria	Comm...	0-18 Y	31 biological mo biological fathers of 33 different fa	The racial compositior White and 47.5% Afric self-identifying as "oth	Not specified	Transit...	** 15 total total components into 3 cat Topics distilled from participant intervi curriculum of a parenting intervention housing: child compliance, parenting	Combination	Multiple compo	In Person	faculty members riage and family			n/a
DMR	655	Kerker	2024	USA, NYC	Good/Fair	Mixed Methods	DAP 1 = 18 from focus groups; 53 eligible for intervention and 21 enrolled; DAP 2 =70 eligible and 31 enrolled; DAP 3= 37 eligible and 10	Pregnant women and mothers of children age 1 yr or younger residing in a family shelter; and two-thirds reported having 1 (33%) or 2 (33%) children. The majority were single (69%) and had earned a High School education (54%). Three-quarters of participants used Medicaid	Eligible women were English speaking residents of a family shelter who were 18 years of age or older, did not have an untreated mental illness (including substance abuse), and were pregnant at the time of the study (during the study, eligibility	NR	Shelters	0-5 Y	NR with socio-demogra phics, but all women (based on researchers' identification)	s Black (47%) or Hispanic/Latina (41%),	NR	Episodic	The Dynamic Adaptation Process (DAP) includes identifying core ele- ments and adaptable characteristics of an EBI, and sup- porting implementation through training and fidelity monitoring (Aarons et al., 2012). The Exploration, Pre- paration, Implementation and Sustainment (EPIS) model consists of four stages (Aarons et	Combination	Multiple co...	Hybrid	Shelter Staff	Mental He...	Social Sup...	"Satisfaction/Feedback Similar to the previous DAP, all 6 staff who completed the post measures reported being very satisfied with SIS and indicated that SIS was helpful in enhancing their ability to help clients manage or reduce their stress." Also presented the number for each DAP e.g., 9/10 staff reported benefits for staff (see annotations). "Ratings on the 21 participant surveys were high, although slightly lower than in previous DAPs. In the participant debrief sessions, some participants expressed a preference for meeting in person, but others appreciated the convenience of the virtual format." Clients felt more comfortable talking about PPD; participants also reportedly benefits including increased socialization; no quantifiable outcome measures in health and wellbeing; In DAP 1 "Participants were highly satisfie with the intervention, with 7 out of 7
JK	655	Kerker	2024	USA	Good/fair	Mixed ...	DAP 1: 53 eligibl dents, 31 (44%) those, 18 (58%) completed at lea (27%) consented (26 or 37%) of th of those, 21 (81	Mothers with children 1 or you	Eligible women were English speaking resident years of age or older, did illness (including substan the time of the study (duri were expanded to include age or younger)	not specified	Shelters	0-5 Y	Women (gender	Black (47%) or Hispar (41%), 11% not specif	not specified, alt	Episodic	"ROSE is a 4-8 session, manualized i prevent postpartum depression by inc decreasing stress and increasing effe version of ROSE that had previously b natal women within the first year of th adapt the intervention name, adapted the four EPIS stages, and feedback in	Combination	Multiple compo	Hybrid	Shelter Staff	Mental Health	Social Support	DAP 1: "Participants were highly satisfied with the intervention, with 7 out of 7 participants indicating that SIS helped them with the stress of a new baby, with effectively asking for help, and with identifying a support system a little or a great deal; participants responded positively to all other satisfaction questions as well." DAP 2: "Similar to DAP 1, rat satisfaction surveys were high. In the debrief sessions
DMR	381	Williams-Arya	2021	USA, Cincinnati	Fair	Qualitati ve	53	Parents of children experiencing homelessness and living in shelters (recruited from 4 shelters)	NR	NR	Shelters	Unspecif ied	51 were female; 2 male	66% African American, 30% white, 3% multiracial, and 1% other. (consistent with city demographics)	NR	Episodic	Across the 5 GLA sessions, family and child needs and priorities while in shelter focused on 4 major themes: (1) job and housing stability, (2) education and skill development, (3) emotional support, and (4) improving shelter life. ****informed intervention and policy action (see outcome results)	Combination	Multiple co...	Hybrid	More than ...	Housing St...	Quality of ...	"the Task Force developed a streamlined process for families to access childcare via 4C for Children in Cincinnati, an agency that coordinates childcare referrals for Cincinnati families, so that parents could attend job and housing interviews without having to bring their children. To enhance child emotional and learning supports as well as improve shelter life, the Play & Learn parenting support program, delivered by 4C, was piloted in 3 family shelters. Play & Learn groups, which are attended by parents and their young (birth to 5 years old) children, promote positive family interactions by teaching parents about developmental milestones, how to prepare their children for school, and how children learn through play via hands-on, experiential activities. To improve shelter life and support education and skills development for sheltered children, the Task Force also facilitated the hiring of staff to coordinate children's programming at the family shelters to increase children's activities and services. Furthermore, the Task Force has done impressive work with the local school districts to ensure children can stay in their home schools"
JK	381	Williams-Arya	2021	USA (Ohio)	Fair	Qualit...	53	Parents residing in 4 family h	not specified	Not specified	Shelters	Unspe...	51 female, 2 not	The sample was cons with the demographic homeless shelters,wh 30% white, 3% multira	not specified	Episodic	Participants participated in a collabora and priorities while in shelter focused themes: (1) job and housing stability, (and skill development, (3) emotional s (4) improving shelter life.	Combination	Multiple compo	Hybrid	More than one	Housing Stabili	Quality of Life	The GLA-generated recommendations described in this study drove both policy and practice changes at the Cincinnati family homeless shelters. Find- ings contributed to Cincinnati Strategies to End Homelessness convening a "Cincinnati Solutions for Family Homelessness Children's Task Force" in fall 2015, and provided the blueprint for the task force work.... Task Force developed a streamlined process for families to ac- cess childcare via 4C for Children in Cincinnati, an agency that coordinates childcare referrals for Cincinnati families, so that parents could attend job and housing interviews without having to bring their children. To enhance child emotional and

Barriers to Implementation	Facilitators of Success	Sustainability	Definition of Homelessness Used	Study Limitations	Notes			
transportation and the multi-hour time commitment of visiting<br>with family made visits to the museum challenging aside from special occasions	Active listening with families, flexibility in working hours (i.e. late evenings and weekends, accomm	Science Together have visibility, input, and buy-in from various levels<br>genuine investment beyond a federal grant will continue to fund acti<br>ter" (p. 306) does not specify how this program will be funded after fe	Families staying in temporary shelters for 3-4 months while working, saving mo<br>into more permanent housing situations. Article acknowledges there is no one	Families only stayed 3-4 months which made transition difficult, challenges of safely providing hands-on activities during the coronavirus pandemic made strategy shift to making engagements shorter in duration and more accessible				
Transportation issues and high turnover at shelter	Iterative feedback loop of participant feedback	Does not believe in sustainability at a federal level, but rather throug	Families stay temporarily at the FMF shelter while working, saving money, and<br>into more permanent housing situations.	No outcomes listed; no methodology detailed; no validated measures used				
Facilitators experienced challenges and enablers to delivering this<br>intervention with fidelity, including high support needs, language barriers,<br>drug use of participants, and challenges related to the context in<br>which the program is delivered, such as communication with staff and<br>availability of training spaces (pg 8), non-attendance included a lack of external incentives,<br>lack of housing service availability to support young people to<br>attend session, and individual characteristics of the young people (pg 5)	high levels of commitment to the partnership, mutual<br>respect for the unique skills, expertise, and perspectives provided by<br>the different stakeholders, and building and maintaining trust, logic models were iterative and fluid	Long-term sustainability plans were built into the<br>partnership from the outset with a focus on capacity building in frontl<br>staff, including support workers and specialist learning, skills, and wo<br>(LSW) coaches.	Youth were living in a temporary shelter accommodation , paper does not defin<br>of structural barriers and societal factors including a shortage of<br>affordable housing, family breakdown, inadequate education and health<br>care, lack of jobs, low paying jobs, poverty, and socioeconomic status" pg 1	not specified				
Facilitators experienced challenges and enablers to delivering this<br>intervention with fidelity, including high support needs, language barriers, drug use of participants, and challenges re<br>which the program is delivered, such as communication with staff and<br>availability of training spaces.	MST4Life™ was designed with a flexible delivery model, we were<br>able to adjust to meet changing needs and priorities of the Service<br>over time. Having open communication between project partners<br>about who the program was intended for and how it is linked to other<br>services/programs offered by the housing service also helped to<br>ensure MST4Life™ led to unexpected opportunities, such as participation in the program counting<br>skills training award.	Long-term sustainability plans were built into the<br>partnership from the outset with a focus on capacity building in frontl<br>staff, including support workers and specialist learning, skills, and wo<br>(LSW) coaches	NA	Did not have any validated measures for mental skills and strengths	No health outcomes?			
although many evidence-informed health programs address-<br>ing parent–child health or family planning among pregnant<br>and parenting youth exist,24–29 few have been designed,<br>implemented, or evaluated for historically marginalized<br>communities, such as YEH. Implementation of an existing program with fidelity is a challenge because all stakeholde<br>emphasized the need for real-time program adaptability based<br>on the particular and fluid needs of youth in the program (pg 487)	Sustained relationships between youth and program staff, availability of childcare during groups, in	Sustained relationships between youth and staff, otherwise not speci	running away, being thrown out of the<br>home, couch surfing, rough sleeping (sleeping on the street)<br>and/or staying in shelter (pg 1)	Study reported on the process and<br>findings from stakeholders in a single community organiza-<br>tion in a particular geographic location; findings regarding<br>programmatic preferences may not be generalizable to other<br>settings and populations, process we outline here is time intensive and required sig-<br>nificant trust building between partners which may not be<br>feasible for all programs (pg 487)	5 youth had a child living with them in shelter. Their ages are not specified, but this may mitigate the exclusion c			
First,<br>although many evidence-informed health programs addressing parent–child health or family planning among pregna<br>and parenting youth exist,24–29 few have been designed,<br>implemented, or evaluated for historically marginalized<br>communities, such as YEH.21–23 Additionally, all stakeholders<br>emphasized the need for real-time program adaptability based<br>on the particular and fluid needs of youth in the program.<br>Thus, implementation of an existing program with fidelity<br>could pose a challenge in this unique population	We used multiple forms of triangulation and drew on existing conceptual frameworks to guide analyses. Aligning with<br>the tenets of CBPR, community stakeholders were engaged<br>throughout the process from planning the needs assessment,<br>conducting analyses, program implementation, and sharing<br>and disseminating findings. We reported on the process and<br>findings from stakeholders in a single community organization in a particular geographic location; findings regarding<br>programmatic preferences may not be generalizable to other<br>settings and populations							
COVID-19 Pandemic caused meetings to be held virtually, however, they were able to adapt to these conditions and	Inclusive and diverse community, leadership through a feminist lens, community buy-in	Reports receiving funding from University of Greenwich and Trust Lo	References temporary accomodations system in UK, does not specify further	not specified				
COVID-19 Pandemic	Community ties	Dependent on local politics– does not note on sustainability other tha	Does not specify	Does not detail methodoloy or outcome measures use. No mention of validated measures or structured analyses	Wondering if we should exclude as there are no health outcomes detailed			
Further, when research and service endeavors are<br>brought together, there may be some inherent clashes of values that need to be dealt with<br>prior to collaborative partnerships moving forward. Finally, for many provider staff,<br>research activities often have an evaluative feel that is feared. Trust between research staff<br>and providers is frequently an issue. Providers may feel protective of clients and question<br>competency and sensitivity of researchers. For example, concerns may be raised that<br>research activities will interfere with the clinical relationship or that clients may experience<br>anxiety or discomfort as a result of participating in research interviews pg 3	application of service into "real world" settings rather than a tightly controlled trial, involvement of f	Collaborative research promotes inclusion, maximizes<br>community resources and strengths, and creates opportunities for ne<br>(Jacobsen & Rugeley, 2007) that can endure beyond the life of a giv	Youth homelessness characterized as<br>"disruption in housing associated with youth mental health difficulties, educational<br>underachievement, substance abuse, and numerous other health-related problems" pg 2					
ignificant								
However, they believed that<br>improvements could be made to the recruitment plan by advertising the program via posters,<br>timetables, and leaflets. One young person believed that better advertising would lead to<br>Int. J. Environ. Res. Public Health 2022, 19, 3320 9 of 17<br>more young people taking part: "If they advertised it a lot more then people would attenintervention with fidelity, inclu<br>which the program is delivered, such as communication with staff and<br>availability of training spaces.	Mutually beneficial community partnerships, high attendance levels	NA	1,2]. It is a complex issue, defined as the lack<br>of a "fixed, regular, and an adequate nighttime residence"	no validated measures	Intervention delivered in residential facility (dormitory setting), not their own homes			
However, they believed that<br>improvements could be made to the recruitment plan by advertising the program via posters,<br>timetables, and leaflets. One young person believed that better advertising would lead to<br>Int. J. Environ. Res. Public Health 2022, 19, 3320 9 of 17<br>more young people taking part: "If they advertised it a lot more then people would attenintervention with fidelity, inclu<br>which the program is delivered such as communication with staff and	High level of attendance and feasibility; mutually beneficial partnerships	NA	Young people were invited to participate in the pilot MST4Life™ program if the	Qualitative methods not described				
Extra support needed---"interventionists should realize that many common emotion regulation strategies (e.g., walking away to cool down, spending alone time in one's room) are more challenging to implement in a transitional housing context" "A commonly cited challenge was that parents and their children could be negatively impacted by the par- enting practices and behaviors of other families in the housing community."	Conducting a needs assessment first; evidence-based parenting interventions with fidelity does not necessarily conflict with delivering relevant parent training to homeless families in transitional housing; importance of including lived experience	NA	"Transitional housing is a temporary, service-intensive form of supportive housing where homeless persons are provided access to resources and given the opportunity for stabilization with the goal of moving into permanent housing (Burt, 2006; HUD, 2008). These programs allow a stay of up to 24 months, though this may vary across programs. Common transitional housing services include case management, tenant education, and provision of basic needs like food and clothing (Burt, 2006). While such services may also include access to parent education, it is unlikely these will be programs with an evidence base for their relevance and efficacy"	Non-representative sample (but it was a qualitative study); did not explore other subgroup differences race/ethnicity; did not elaborate of which participants had children living with them (although they briefly stated that most typically did);Subsequent research<br>could develop a quantitative assessment based on the findings of<br>this study and ask participants to rate their level of endorsement for<br>each suggested component. It should also be noted that the inter-<br>rater reliability achieved during the coding process (i.e., k= 0.71)<br>was less than perfect, and so interpretation of the findings should<br>reflect the level of confidence appropriate for this rating. Challenges of CBPR: "CBPR approach by enhancing collaboration with homeless par-<br>ents. Yet, families in transitional housing face numerous obliga-<br>tions and may not wish to participate equitably in all phases of the<br>research process. While achieving greater participation is a valu-<br>able goal, researchers must also take care not to impose participa-<br>tion on a research project (Greenwood, Whyte, & Harkavy, 1993).<br>Importantly, while this study focused on understanding the unique<br>and valuable perspective of homeless parents, future research may	preassessment is Qualitative of a larger<br>mixed methods study			
Constraints due to rules of transitional housing: Service providers "need to remain aware of the unique<br>context of transitional housing and work with parents to find<br>realistic and acceptable ways of using the parenting skills in this<br>environment; some emotional regulation strategies are difficult to implement e.g, walk away/cooling down due to size	Balanced approach to addressing the tension between intervention fidelity and adaptation, CBPR	N/a	"Transitional housing is a temporary, service-intensive form of supportive housing where homeless persons are provided access to resources and given the opportunity for stabilization with the goal of moving into permanent housing (Burt, 2006; HUD, 2008). These programs allow a stay of up to 24 months, though this may vary across programs. Common transitional housing services include case management, tenant education, and provision of basic needs like food and clothing"	Qualitative study w/ non-representative sample, be cautious w/ generalizing findings; semi structured interview approach left room fo	Limitation is that child "typically" lives with parent but that is not explained, intervention is specifically for parents			
COVID-19 pandemic disrupted study- had to change delivery mode; time-intensive; challenges with recruitment and completion of sessions	Iterative process, e.g., changing language in the intervention to better suit the population and their circumstances; feedback loop and high fidelity; included "organizational stakeholders in identifying appropriate implementation strategies, and outlined the steps to do so."	created scalable plans in the sustainment phase; "Based on knowledge gained over the 3 DAP cycles, we have worked with the shelter system to develop a plan to scale and sustain SIS throughout the shelters. Our current plan is to train staff from across the system 3 times per year, and to have our team psychologist be available upon request; the prenatal and postnatal manuals and workbooks have been translated into Spanish to expand access for those with limited English proficienc living in shelters. This plan is in early stages, and we will monitor our progress and make modification as necessary."	Not outright defined; implied through use of "homeless shelters"	very complicated study with multiple stages and refinements- would have been beneficial to have a visual representation e.g., flow diagram; no quantitative measures reported on health and wellbeing outcomes (either anecdoctal or an overall summary of the staff or client population ratings)- it would be nice to see what the satisfication surveys looked like and what questions were asked as well as presented the results in a table; intervention only delivered in English, which excluded a significant proportion the family homelessness population; The SIS adaptation was limited by a relatively small number of sites in a very large system; challenges with recruiting and retaining participants; they did not look at technology literacy and how that could be a barrier for participants in the virtual format; the study didn't show how well it could be scaled across the system (according to authors)	" "Engaging partners early and continually and understanding the potential participant population were essential components to our Exploration Stage. Specifically, this stage included (1) engaging senior leadership and social work staff and (2) understanding the needs of shelter staff and shelter residents, as well as the acceptability of a PPD intervention like ROSE."" 4<br>intervention cycles with iterative process-			
Adapted intervention to virtual format b/c of COVID lockdown: it was difficult for some participants to find a private sp	"Engaging partners early and continually and understanding<br>the potential participant population were essential compo-<br>nents to our Exploration Stage"; shelter staff were given comprehensive trauma-informed training:<br>included didactics regarding the risks and protective fac-<br>tors for postpartum depression, the aims of the research<br>study, step-by-step teaching of the manualized interven-<br>tion , and live role plays to practice administration of the	"we have worked with the shelter system to develop a plan to<br>scale and sustain SIS throughout the shelters. Our current<br>plan is to train staff from across the system 3 times per year,<br>and to have our team psychologist be available upon<br>request; the prenatal and postnatal manuals and workbooks<br>have been translated into Spanish to expand access for those<br>with limited English pro ciency living in shelters	not specified	SIS adaptation was limited by small number of sites in large system, did not describe how SIS can be scaled, not translated in Spanish at time of the study, challenges with recru		iting participants		
Not reported; only context of barriers was the barriers faced by participants in using services; mode of delivery could be a barrier if the aforementioned barriers by participants are not considered	GLA approach; shelter staff provided sensitivity training for participants with trauma although type of training not specified or cited; data collected at individual and group levels; "The strengths of this study include that GLA methodology allowed for gathering of data at both the individual and group levels, with the parents themselves processing the information and creating the results, as opposed to the researcher- directed analytic approach used for standard focus groups. Detailed, descriptive information was able to be gathered about parents' experiences of homelessness, conveyed in their own words. Benefits of GLA include the ability to collect information from a large number of parents in a more expeditious and time-efficient manner than is possible with in-dividual interviews. The GLA process is also highly interactive and allows parents more opportunity for clarification than do paper surveys. " the GLA<br>process allows parents 3 platforms to communicate<br>their feedback: in written form on the posters, in<br>small group discussion, and in whole group dis-<br>cussion.	Not reported	McKinney-Vento	eligibility criteria was unclear and ages of children- more demographics even if a descriptive range would help (e.g., children were between ages x and y); only meets inclusion criteria because the results thematically discuss child development; no quantifying of individual vs. group-level data- presence or absence of themes, so any outlier data may have been lost in group-level data analysis; sample was self-selected (as parents residing in the shelter chose whether or not to attend the GLA sessions), and so may not be fully representative of families residing at the shelters. hetergeneous because of 3/4 shelters were different types so responses could vary by shelter type	Study does not report age of participants' children, but can be inferred from results and developed one intervention which targets ages 0-5 yrs			

not specified	GLA methodology allowed for gathering of data at both the individual and group levels, with the parents themselves processing the information and creating the results, as opposed to the researcher-directed analytic approach used for standard focus groups. Detailed, descriptive information was able to be gathered about parents' experiences of homelessness, conveyed in their own words. Benefits of GLA include the ability to collect information from a large number of parents in a more expeditious and time-efficient manner than is possible with individual interviews.The GLA process is also highly interactiveandallowsparentsmoreopportunityfor clarification than do paper surveys Furthermore	Not specified	Not specified but McKinney-Vento Act is IMPLIED in community action section	The study was conducted in one city so findings may not be generalizable, the sample was self selected so it may not be representative of all families in shelter, individual outlier themes may have gotten lost in group analysis, some responses m

Table S5. Study Definitions of Homelessness

Author	Year	Homelessness Definition
Cawley[50]	2022	• Temporary shelter where families reside for about 3-4 months while working, saving money, and preparing to move into permanent housing<br>• Acknowledges that no single definition of homelessness exists, and that this was the term families preferred to describe their situation
Cumming[51]	2022	• Temporary shelter accommodation<br>• Does not define homelessness further but includes factors that lead to youth homelessness: "…a complex set of structural barriers and societal factors including a shortage of affordable housing, family breakdown, inadequate education and healthcare, lack of jobs, low paying jobs, poverty, and socioeconomic status"
Cumming [52]	2022	• The lack of a "fixed, regular, and adequate nighttime residence" – they cited definition from the U.S. 2017 Annual Homeless Assessment Report (AHAR) to Congress. It's also the definition from McKinney-Vento Act.
Gewirtz O'Brien[53]	2022	• "Running away, being thrown out of the home, couch surfing, rough sleeping (sleeping on the street) and/or staying in shelter"<br>• "Transitional housing programs provide interim, longer-term stability (in this case, for up to 18 months) and supports to prepare individuals to transition to permanent housing."<br>• In this study, the program was specifically for pregnant and/or parenting homeless youth.
Marziale[54]	2024	• No definition or use of term *homeless*, but uses alternative terms without further detail<br>• "Temporary shelter accommodation"<br>• "NRPF children"<br>• Does not explain other terms further (e.g., no mention of the Homelessness Act 2002 and Homeless Reduction Act 2017)
McKay[55]	2010	• No definition, but characterize youth homelessness as: "...disruption in housing associated with youth mental health difficulties, educational underachievement, substance abuse, and numerous other health-related problems"<br>• Referred to participants as those "who reside in family homeless shelters"
Holtrop[56]	2015	• Not specified<br>• "Transitional housing is a temporary, service-intensive form of supportive housing where homeless persons are provided access to resources and given the opportunity for stabilization with the goal of moving into permanent housing. These programs allow a stay of up to 24 months, although this may vary across programs. Common transitional housing services include case management, tenant education, and provision of basic needs like food and clothing."
Kerker[57]	2024	• Not specified, but implied through use of "homeless shelters" and "residents of a family shelter"

Williams-Arya[58]	2021	• Not specified<br>• Cited statistic: “7028 people slept in an emergency shelter or in a place not meant for human habitation.”<br>• McKinney-Vento Act is implied in one section

Table S6. Study Quality Assessment Ratings and Study Limitations

Author	NHLBI*	MMAT**	Study Limitations
Cawley[50]	Poor	Doesn’t pass screening questions	• No formal evaluation; detailed findings from the iterative co-development interviews were not reported<br>• Programs built for sustainability and accessibility, but no measures reported<br>• Sample size and demographics of target population not reported<br>• No actual data reported (e.g., quotations) to support research question; study was narrative<br>• An assets- and needs-based assessment of the museum was reportedly conducted; however, there is no available documentation regarding the methodology employed or the results obtained
Cumming[51]	Fair	Poor	• Participant retention rate was less than 50%<br>• Socio-demographics not reported (e.g., gender, ethnicity)<br>• Cannot fully determine whether eligible participants who met all the prespecified entry criteria were enrolled<br>• Results from the fidelity assessment (27 rating items) were not presented<br>• Rationale for study design not presented<br>• Some interpretations of results not substantiated by the data
Cumming [52]	Poor	Poor	• No evaluation with quantitative statistical analyses except for program attendance<br>• Small sample size; 19.2% enrollment<br>• Cohort design, non-randomized into intervention<br>• No validated measures reported<br>• Data source is unclear when referencing diary room entries and focus groups<br>• Unclear if there were differences among the two sites in Phase 1 (important to know in terms of enrollment and pilot delivery)
Gewirtz O'Brien[53]	Fair	Fair	• No stated eligibility criteria<br>• Small sample size<br>• Although a qualitative study, there were results or analyses missing that could have added more context, e.g., quantifying key content areas by group (youth, staff, and community experts)<br>• Questionnaire data not reported and triangulation methodology not clearly reported<br>• Did not consistently label quotes from participants
Marziale[54]	Poor	Doesn’t pass screening questions	• No stated study question or objective<br>• No stated eligibility criteria<br>• No validated measures reported<br>• No pre/post scientific analysis

			• No study design or formal evaluation<br>• No detailed methods (e.g., data analysis) or strategies for the co-development process
McKay[55]	Poor	Doesn't pass screening questions	• No stated eligibility criteria<br>• No outcome measures prespecified, clearly defined, valid, reliable, or assessed consistently across all study participants<br>• No evidence presented that matched the resulting primary outcome (i.e., reduction in youth mental health symptoms)<br>• Could not determine several criteria in quality assessment expected for study design<br>• Co-development of the intervention itself not described in detail
Holtrop[56]	Good/Fair	Good	• Qualitative study with non-representative sample<br>• Inter-rater reliability (0.71) did not indicate perfect agreement<br>• Did not explore subgroups in racial/ethnic demographics<br>• Stated children "typically" lived in shelter with parents, but did not elaborate on which participants had children living with them<br>• Intervention provider is unclear
Kerker[57]	Good/Fair	Fair	• No quantitative measures reported on health and well-being outcomes or satisfaction with the intervention<br>• Delivered intervention only in English<br>• Intervention adaptation delivered in a small number of sites in a very large system<br>• Did not describe how intervention could be scaled across systems<br>• No visual representation of intricate adaptation processes or data<br>• Challenges with recruiting and retaining participants<br>• Did not address technological barriers in virtual format<br>• Study aim could be stated more clearly<br>• Rationale for study design not provided
Williams-Arya[58]	Fair	Fair	• No stated eligibility criteria<br>• Self-selected sample<br>• Did not report ages of children<br>• Unclear whether racial demographics reflect sample or general population of family shelters<br>• Individual- vs. group-level data not quantified<br>• Outlier themes may have gotten lost in group-level analysis<br>• Heterogeneity due to shelter type; Some responses may have been particular to a specific shelter

*NHLBI (National Heart, Lung, and Blood Institute)
** MMAT (The Mixed Methods Appraisal Tool): DMR and JK assessed the articles with the MMAT for comparison. Results were similar to those of the NHLBI, except for the MMAT, which excluded 3 articles during screening.

Table S7. Quality Assessments

Quality Assessment Tool for Before-After (Pre-Post) Studies With No Control Group—Cawley 2022

Criteria	SL	SL	SL	JK	JK	JK
	**Yes**	**No**	**Other (CD, NR, NA)***	**Yes**	**No**	**Other (CD, NR, NA)***
1. Was the study question or objective clearly stated?	Y			yes		
2. Were eligibility/selection criteria for the study population prespecified and clearly described?	Y			yes		

3. Were the participants in the study representative of those who would be eligible for the test/service/intervention in the general or clinical population of interest?	Y			Yes		
4. Were all eligible participants that met the prespecified entry criteria enrolled?			CD			CD
5. Was the sample size sufficiently large to provide confidence in the findings?			NA			NA
6. Was the test/service/intervention clearly described and delivered	Y			Y		

consistently across the study population?						
7. Were the outcome measures prespecified, clearly defined, valid, reliable, and assessed consistently across all study participants?			CD			CD
8. Were the people assessing the outcomes blinded to the participants' exposures/interventions?			NA			NA
9. Was the loss to follow-up after baseline 20% or less? Were those lost to follow-up			NA			NA

accounted for in the analysis?						
10. Did the statistical methods examine changes in outcome measures from before to after the intervention? Were statistical tests done that provided p values for the pre-to-post changes?			NA			NA
11. Were outcome measures of interest taken multiple times before the intervention and multiple times after the intervention (i.e., did they use an interrupted			NA			NA

time-series design)?						
12. If the intervention was conducted at a group level (e.g., a whole hospital, a community, etc.) did the statistical analysis take into account the use of individual-level data to determine effects at the group level?			NA			NA

Quality Rating (Good, Fair, or Poor) (see guidance)

Rater #1 Initials: SL- Poor

Rater #2 Initials: JK - Poor

Additional Comments (If POOR, please state why): No methodology described. Sample size and demographics not reported.

Quality Assessment Tool for Before-After (Pre-Post) Studies With No Control Group—Cumming 2022 (#1026)

Criteria	SL	SL	SL	JK	JK	JK
	**Yes**	**No**	**Other (CD, NR, NA)***	**Yes**	**No**	**Other (CD, NR, NA)***
1. Was the study question or objective clearly stated?	Y			yes		
2. Were eligibility/selection criteria for the study population prespecified and clearly described?	Y			yes		
3. Were the participants in the study representative of those who would be eligible for the test/service/intervention in the general or	Y			Yes		

clinical population of interest?						
4. Were all eligible participants that met the prespecified entry criteria enrolled?			CD			CD
5. Was the sample size sufficiently large to provide confidence in the findings?			NA			NA
6. Was the test/service/intervent ion clearly described and delivered consistently across the study population?	Y			Yes		
7. Were the outcome measures prespecified, clearly defined, valid, reliable, and assessed consistently across all study participants?			NA			NA
8. Were the people assessing the outcomes blinded to the participants'			NA			NA

exposures/interventions?						
9. Was the loss to follow-up after baseline 20% or less? Were those lost to follow-up accounted for in the analysis?			NA			NA
10. Did the statistical methods examine changes in outcome measures from before to after the intervention? Were statistical tests done that provided p values for the pre-to-post changes?			NA			NA
11. Were outcome measures of interest taken multiple times before the intervention and multiple times after the intervention (i.e., did they use an interrupted time-series design)?			NA			NA
12. If the intervention was conducted at a group level (e.g., a whole hospital, a			NA			NA

community, etc.) did the statistical analysis take into account the use of individual-level data to determine effects at the group level?						

Quality Rating (Good, Fair, or Poor) (see guidance)

Rater #1 Initials: SL- Fair

Rater #2 Initials: JK- Fair

Additional Comments (If POOR, please state why):

Socio-demographics not reported. Cannot fully determine whether eligible participants who met all the prespecified entry criteria were enrolled.

Quality Assessment Tool for Before-After (Pre-Post) Studies With No Control Group—Cumming 2022 (#68)

Criteria	JK	JK	JK	SL	SL	SL
	**Yes**	**No**	**Other (CD, NR, NA)***	**Yes**	**No**	**Other (CD, NR, NA)***
1. Was the study question or objective clearly stated?	Y			Y		
2. Were eligibility/selection criteria for the study population prespecified and clearly described?	Y			Y		
3. Were the participants in the study representative of those who would be eligible for the test/service/intervention in the general or clinical population of interest?			CD			CD
4. Were all eligible participants that met the		N			N	

prespecified entry criteria enrolled?						
5. Was the sample size sufficiently large to provide confidence in the findings?			CD			CD
6. Was the test/service/intervention clearly described and delivered consistently across the study population?	Y			Y		
7. Were the outcome measures prespecified, clearly defined, valid, reliable, and assessed consistently across all study participants?		N			N	
8. Were the people assessing the outcomes blinded to the participants' exposures/interventions?			CD			CD
9. Was the loss to follow-up after baseline 20% or less? Were those lost to follow-up accounted for in the analysis?			CD			CD
10. Did the statistical methods examine changes in outcome measures from before to after the			NA			NA

intervention? Were statistical tests done that provided p values for the pre-to-post changes?						
11. Were outcome measures of interest taken multiple times before the intervention and multiple times after the intervention (i.e., did they use an interrupted time-series design)?		N			N	
12. If the intervention was conducted at a group level (e.g., a whole hospital, a community, etc.) did the statistical analysis take into account the use of individual-level data to determine effects at the group level?			NA			NA

Quality Rating (Good, Fair, or Poor) (see guidance)

Rater #1 Initials: SL Poor

Rater #2 Initials: JK Poor

Additional Comments (If POOR, please state why): No evaluation with quantitative analyses, no validated measures reported.

Quality Assessment Tool for Before-After (Pre-Post) Studies With No Control Group—Gewirtz O'Brien 2022

Criteria	SL	SL	SL	JK	JK	JK
	**Yes**	**No**	**Other (CD, NR, NA)***	**Yes**	**No**	**Other (CD, NR, NA)***
1. Was the study question or objective clearly stated?	Y			yes		
2. Were eligibility/selection criteria for the study population prespecified and clearly described?			CD			CD
3. Were the participants in the study representative of those who would be eligible for the test/service/intervention in the general or	Y			Yes		

clinical population of interest?						
4. Were all eligible participants that met the prespecified entry criteria enrolled?			NA			NA
5. Was the sample size sufficiently large to provide confidence in the findings?			CD			CD
6. Was the test/service/intervention clearly described and delivered consistently across the study population?			NA			NA
7. Were the outcome measures prespecified, clearly defined, valid, reliable, and assessed consistently across all study participants?		N		N		
8. Were the people assessing the outcomes blinded to the participants' exposures/interventions?			NA			NA

9. Was the loss to follow-up after baseline 20% or less? Were those lost to follow-up accounted for in the analysis?			NA			NA
10. Did the statistical methods examine changes in outcome measures from before to after the intervention? Were statistical tests done that provided p values for the pre-to-post changes?			NA			NA
11. Were outcome measures of interest taken multiple times before the intervention and multiple times after the intervention (i.e., did they use an interrupted time-series design)?			NA			NA
12. If the intervention was conducted at a group level (e.g., a whole hospital, a community, etc.) did the statistical analysis take into account the use of individual-level data to determine			NA			NA

effects at the group level?						

Quality Rating (Good, Fair, or Poor) (see guidance)

Rater #1 Initials: SL - Fair

Rater #2 Initials: JK - Fair

Additional Comments (If POOR, please state why):

No eligibility criteria described. Could not determine some of the criteria.

Quality Assessment Tool for Before-After (Pre-Post) Studies With No Control Group—Marziale 2024

Criteria	SL			JK		
	Yes	No	Other (CD, NR, NA)*	Yes	No	Other (CD, NR, NA)*
1. Was the study question or objective clearly stated?	Y			yes		
2. Were eligibility/selection criteria for the study population prespecified and clearly described?		N			No	
3. Were the participants in the study representative of those who would be eligible for the test/service/intervention in the general or clinical population of interest?			CD			CD
4. Were all eligible participants that met the prespecified entry criteria enrolled?			NA			NA

5. Was the sample size sufficiently large to provide confidence in the findings?			NA			NA
6. Was the test/service/intervention clearly described and delivered consistently across the study population?			CD			CD
7. Were the outcome measures prespecified, clearly defined, valid, reliable, and assessed consistently across all study participants?		N			No	
8. Were the people assessing the outcomes blinded to the participants' exposures/interventions?			CD			CD
9. Was the loss to follow-up after baseline 20% or less? Were those lost to follow-up accounted for in the analysis?			CD			CD
10. Did the statistical methods examine changes in outcome measures from before to after the intervention? Were statistical tests done that provided p values for the pre-to-post changes?			CD			CD
11. Were outcome measures of interest taken multiple times before the intervention and multiple times after the intervention (i.e., did they use an interrupted time-series design)?			CD			CD

12. If the intervention was conducted at a group level (e.g., a whole hospital, a community, etc.) did the statistical analysis take into account the use of individual-level data to determine effects at the group level?			CD			CD

Quality Rating (Good, Fair, or Poor) (see guidance)

Rater #1 Initials: SL– Poor

Rater #2 Initials: JK– Poor

Article describes co-developed research projects that were underway at the time of publication but does not include specific information about their methodology, participant recruitment, etc.

Additional Comments (If POOR, please state why): Did not have clear methodology and results section listing the analysis

Quality Assessment Tool for Before-After (Pre-Post) Studies With No Control Group—McKay 2010

Criteria	SL			JK		
	**Yes**	**No**	**Other (CD, NR, NA)***	**Yes**	**No**	**Other (CD, NR, NA)***
1. Was the study question or objective clearly stated?	Y			yes		
2. Were eligibility/selection criteria for the study population prespecified and clearly described?		N			No	
3. Were the participants in the study representative of those who would be eligible for the test/service/intervention in the general or clinical population of interest?			CD			CD
4. Were all eligible participants that met the prespecified entry criteria enrolled?			NA			NA

5. Was the sample size sufficiently large to provide confidence in the findings?			NA			NA
6. Was the test/service/intervention clearly described and delivered consistently across the study population?			CD			CD
7. Were the outcome measures prespecified, clearly defined, valid, reliable, and assessed consistently across all study participants?		N			No	
8. Were the people assessing the outcomes blinded to the participants' exposures/interventions?			CD			CD
9. Was the loss to follow-up after baseline 20% or less? Were those lost to follow-up accounted for in the analysis?			CD			CD
10. Did the statistical methods examine changes in outcome measures from before to after the intervention? Were statistical tests done that provided p values for the pre-to-post changes?			CD			CD
11. Were outcome measures of interest taken multiple times before the intervention and multiple times after the intervention (i.e., did they			CD			CD

use an interrupted time-series design)?						
12. If the intervention was conducted at a group level (e.g., a whole hospital, a community, etc.) did the statistical analysis take into account the use of individual-level data to determine effects at the group level?			CD			CD

Quality Rating (Good, Fair, or Poor) (see guidance)

Rater #1 Initials: SL– Poor

Rater #2 Initials: JK– Poor

Article describes co-developed research projects that were underway at the time of publication but does not include specific information about their methodology, participant recruitment, etc.

Additional Comments (If POOR, please state why): Was not written as a scientific manuscript and did not have a clear methodology and results section listing the analysis

Quality Assessment Tool for Before-After (Pre-Post) Studies With No Control Group—Holtrop 2015

**Criteria**	**DMR**			**JK**		
	**Yes**	**No**	**Other (CD, NR, NA)***	**Yes**	**No**	**Other (CD, NR, NA)***
1. Was the study question or objective clearly stated?	Yes			yes		
2. Were eligibility/selection criteria for the study population prespecified and clearly described?	Yes			yes		
3. Were the participants in the study representative of those who would be eligible for the test/service/intervention in the general or clinical population of interest?		N			N	
4. Were all eligible participants that met the prespecified entry criteria enrolled?			CD			CD

5. Was the sample size sufficiently large to provide confidence in the findings?			CD			CD
6. Was the test/service/intervention clearly described and delivered consistently across the study population?			NA			NA
7. Were the outcome measures prespecified, clearly defined, valid, reliable, and assessed consistently across all study participants?	Y			Y		
8. Were the people assessing the outcomes blinded to the participants' exposures/interventions?			NA			NA
9. Was the loss to follow-up after baseline 20% or less? Were those lost to follow-up accounted for in the analysis?			NA			NA
10. Did the statistical methods examine changes in outcome measures from before to after the intervention? Were statistical tests done that provided p values for the pre-to-post changes?			NA			NA
11. Were outcome measures of interest taken multiple times before the intervention and multiple times after the intervention (i.e., did they			NA			NA

use an interrupted time-series design)?						
12. If the intervention was conducted at a group level (e.g., a whole hospital, a community, etc.) did the statistical analysis take into account the use of individual-level data to determine effects at the group level?			NA			NA

Quality Rating (Good, Fair, or Poor) (see guidance)

Rater #1 Initials: DMR—Good/Fair

Rater #2 Initials: JK–Good/Fair

Additional Comments (If POOR, please state why):

Data saturation not discussed; demographics of children not presented, including how many lived with them in transitional housing.

No intervention delivered yet.

Some eligible families declined to participate in the study and were still offered treatment as usual.

Quality Assessment Tool for Before-After (Pre-Post) Studies With No Control Group—Kerker 2024

Criteria	DMR			JK		
	**Yes**	**No**	**Other (CD, NR, NA)***	**Yes**	**No**	**Other (CD, NR, NA)***
1. Was the study question or objective clearly stated?	Y			Y		
2. Were eligibility/selection criteria for the study population prespecified and clearly described?			NR (exclusion criteria)			NR
3. Were the participants in the study representative of those who would be eligible for the test/service/intervention in the general or clinical population of interest?			CD			CD
4. Were all eligible participants that met the prespecified entry criteria enrolled?		N			N	

5. Was the sample size sufficiently large to provide confidence in the findings?			CD			CD
6. Was the test/service/intervention clearly described and delivered consistently across the study population?	Y			Y		
7. Were the outcome measures prespecified, clearly defined, valid, reliable, and assessed consistently across all study participants?			CD			CD
8. Were the people assessing the outcomes blinded to the participants' exposures/interventions?			NR			CD
9. Was the loss to follow-up after baseline 20% or less? Were those lost to follow-up accounted for in the analysis?	Y			Y		
10. Did the statistical methods examine changes in outcome measures from before to after the intervention? Were statistical tests done that provided p values for the pre-to-post changes?			CD			NA
11. Were outcome measures of interest taken multiple times before the intervention and multiple times after the intervention (i.e., did they			CD			CD

use an interrupted time-series design)?						
12. If the intervention was conducted at a group level (e.g., a whole hospital, a community, etc.) did the statistical analysis take into account the use of individual-level data to determine effects at the group level?			CD			CD

Quality Rating (Good, Fair, or Poor) (see guidance)

Rater #1 Initials: DMR– Good/Fair

Rater #2 Initials: JK–Good/Fair

Additional Comments (If POOR, please state why):

Impressive article- would have been rated “good” if certain details were reported, such as quantitative analysis, exclusion criteria, etc.

Quality Assessment Tool for Before-After (Pre-Post) Studies With No Control Group—Williams-Arya 2021

Criteria	DMR			JK		
	Yes	No	Other (CD, NR, NA)*	Yes	No	Other (CD, NR, NA)*
1. Was the study question or objective clearly stated?	Y			Y		
2. Were eligibility/selection criteria for the study population prespecified and clearly described?		N			N	
3. Were the participants in the study representative of those who would be eligible for the test/service/intervention in the general or clinical population of interest?			CD			CD
4. Were all eligible participants that met the prespecified entry criteria enrolled?			CD			CD

5. Was the sample size sufficiently large to provide confidence in the findings?			CD			CD
6. Was the test/service/intervention clearly described and delivered consistently across the study population?			CD			CD
7. Were the outcome measures prespecified, clearly defined, valid, reliable, and assessed consistently across all study participants?			NA			NA
8. Were the people assessing the outcomes blinded to the participants' exposures/interventions?			NA			NA
9. Was the loss to follow-up after baseline 20% or less? Were those lost to follow-up accounted for in the analysis?			NA			NA
10. Did the statistical methods examine changes in outcome measures from before to after the intervention? Were statistical tests done that provided p values for the pre-to-post changes?			NA			NA
11. Were outcome measures of interest taken multiple times before the intervention and multiple times after the intervention (i.e., did they			NA			NA

use an interrupted time-series design)?						
12. If the intervention was conducted at a group level (e.g., a whole hospital, a community, etc.) did the statistical analysis take into account the use of individual-level data to determine effects at the group level?			NA			NA

Quality Rating (Good, Fair, or Poor) (see guidance)

Rater #1 Initials: DMR– Fair

Rater #2 Initials: JK– Fair

Additional Comments (If POOR, please state why):

No intervention was developed (although findings informed intervention later on)

No stated eligibility criteria or characteristics of children recruited; no quantified qualitative data (see extraction sheet)

However, they described their qualitative methodologies and analysis in detail.
